# Generation of tumor-initiating cells by exogenous delivery of *OCT4 *transcription factor

**DOI:** 10.1186/bcr3019

**Published:** 2011-09-27

**Authors:** Adriana S Beltran, Ashley G Rivenbark, Bryan T Richardson, Xinni Yuan, Haili Quian, John P Hunt, Eric Zimmerman, Lee M Graves, Pilar Blancafort

**Affiliations:** 1 Department of Pharmacology, The University of North Carolina at Chapel Hill, 120 Mason Farm Road, Chapel Hill, NC 27599, USA; 2 UNC Lineberger Comprehensive Cancer Center, The University of North Carolina at Chapel Hill, 450 West Drive, Chapel Hill, NC 27599, USA; 3 Department of Biochemistry and Biophysics, The University of North Carolina at Chapel Hill, 120 Mason Farm Road, Chapel Hill, NC 27599, USA; 4 State Key Laboratory of Molecular Oncology, Cancer Hospital/Institute, Chinese Academy of Medical Sciences, Pan Jia Yuan Nan Li 17, Chaoyang District, Beijing, 100021, P.R. China; 5 Department of Pathology and Laboratory Medicine, The University of North Carolina at Chapel Hill, 515 Brinkhous-Bullitt Building, Chapel Hill, NC 27599-7365, USA

## Abstract

**Introduction:**

Tumor-initiating cells (TIC) are being extensively studied for their role in tumor etiology, maintenance and resistance to treatment. The isolation of TICs has been limited by the scarcity of this population in the tissue of origin and because the molecular signatures that characterize these cells are not well understood. Herein, we describe the generation of TIC-like cell lines by ectopic expression of the *OCT4 *transcription factor (TF) in primary breast cell preparations.

**Methods:**

*OCT4 *cDNA was over-expressed in four different primary human mammary epithelial (HMEC) breast cell preparations from reduction mammoplasty donors. *OCT4*-transduced breast cells (OTBCs) generated colonies (frequency ~0.01%) in self-renewal conditions (feeder cultures in human embryonic stem cell media). Differentiation assays, immunofluorescence, immunohistochemistry, and flow cytometry were performed to investigate the cell of origin of OTBCs. Serial dilutions of OTBCs were injected in nude mice to address their tumorigenic capabilities. Gene expression microarrays were performed in OTBCs, and the role of downstream targets of OCT4 in maintaining self-renewal was investigated by knock-down experiments.

**Results:**

OTBCs overcame senescence, overexpressed telomerase, and down-regulated *p16^INK4A^*. In differentiation conditions, OTBCs generated populations of both myoepithelial and luminal cells at low frequency, suggesting that the cell of origin of some OTBCs was a bi-potent stem cell. Injection of OTBCs in nude mice generated poorly differentiated breast carcinomas with colonization capabilities. Gene expression microarrays of OTBC lines revealed a gene signature that was over-represented in the claudin-low molecular subtype of breast cancer. Lastly, siRNA-mediated knockdown of OCT4 or downstream embryonic targets of OCT4, such as NANOG and ZIC1, suppressed the ability of OTBCs to self-renew.

**Conclusions:**

Transduction of *OCT4 *in normal breast preparations led to the generation of cell lines possessing tumor-initiating and colonization capabilities. These cells developed high-grade, poorly differentiated breast carcinomas in nude mice. Genome-wide analysis of OTBCs outlined an embryonic TF circuitry that could be operative in TICs, resulting in up-regulation of oncogenes and loss of tumor suppressive functions. These OTBCs represent a patient-specific model system for the discovery of novel oncogenic targets in claudin-low tumors.

## Introduction

Much evidence supports the hypothesis that tumor specimens and tumor cell lines are heterogeneous cell populations comprising a hierarchical organization of cell types [1-3]. Within this hierarchy, a rare population of undifferentiated cells is able to self-renew, proliferate, and develop into more differentiated tumor cells. The population of tumor cells that retain the ability to self-renew and generate tumors is commonly referred to as tumor-initiating cells (TICs) or cancer stem cells [4]. The properties and molecular hallmarks of these cells are not well understood, despite their pivotal role in cancer etiology and resistance to treatment. In breast cancer, prospective TICs have been isolated by flow cytometry by using cell surface antigens, such as CD44 and CD24 [5]. However, the isolation of TICs has been hampered because these cells represent a rare population within the tumor, making it difficult to study their role in tumor biology. Thus, there is a need to develop novel approaches for the isolation and molecular characterization of TICs. These approaches ultimately will facilitate the potential discovery of targeted therapeutics that are specific for tumor cell initiation.

Recent advances in the field suggest that breast tumors belonging to the claudin-low and basal-like intrinsic subtypes are particularly enriched in TIC cell signatures [6,7]. It has been proposed, on the basis of genome-wide gene expression microarray studies, that the more undifferentiated claudin-low and basal-like tumors may originate from stem and early progenitor cells, whereas luminal A and B tumors are possibly generated from more differentiated cell types [8,9]. Claudin-low carcinomas are mostly triple-negative - negative for progesterone receptor (PR), estrogen receptor (ER), and epidermal growth factor receptor 2 (HER2) - and presumably originated from more primitive stem cells [10]. Hallmarks of these tumors include a high enrichment for a CD44^+^/CD24^-/low ^TIC signature, a downregulation of cell junction proteins such as cadherins and claudins, an enrichment in mesenchymal markers, high lymphocyte infiltrations, and high phenotypic resistance to chemotherapy [6,11]. The relationship between breast stem cells and gain of mesenchymal markers is further supported by a recent report that demonstrated that the ectopic expression of transcription factors (TFs) known to promote epithelial-to-mesenchymal transition (EMT) resulted in the generation of breast cells with stem cell properties [12].

Little is known regarding the factors maintaining self-renewal gene programs and tumor initiation in claudin-low and basal-like breast cancers. Evidence suggests that networks of TFs play an essential role in activating self-renewal gene programs, both in human embryonic stem cells (hESCs) and in adult stem cells [13]. Furthermore, some self-renewal TFs are found overexpressed in poorly differentiated and high-grade tumors [13], suggesting that some carcinomas could hijack underlying self-renewal TF machinery to support aberrant proliferation and tumor initiation. Among these TFs, the *OCT4 *gene (also named *POU5F1*) encodes a homeodomain-containing TF, which plays a pivotal role as master regulator or gatekeeper of self-renewal and pluripotency [14]. Importantly, overexpression of *OCT4 *cDNA in a mouse model led to the generation of dysplastic lesions in epithelial tissues, such as the skin and colon, by aberrant expansion of early progenitor cells [15]. Recent papers suggest that OCT4 is expressed in a subpopulation of breast and ovarian cancer cells possessing self-renewal ability [16,17]. Specifically, RNA interference (RNAi)-mediated knockdown of *OCT4 *in tumor lines, such as the breast MCF-7 line, and, most recently, in poorly differentiated epithelial ovarian cancer cell lines resulted in decreased survival [16] and inhibition of tumorigenic potential [17]. Overall, these observations point to an important role for OCT4 in maintaining aberrant tumor cell self-renewal and, possibly, tumor initiation in epithelial tissues.

In this paper, we have investigated the role of OCT4 as a potential driver of self-renewal in the human mammary gland. Lentiviral delivery of *OCT4 *in primary breast human epithelial cell preparations led to the isolation of colonies (frequency of 0.01%) with infinite self-renewal ability. These cells exhibited tumor-initiating properties and metastasized in nude mice. Gene expression microarray analysis of OCT4-overexpressing cells revealed a gene signature that was over-represented in the claudin-low molecular subtype of breast cancer. RNAi-mediated knockdown of OCT4 and embryonic targets of OCT4 in these cells resulted in suppression of the self-renewal ability, outlining the dependence of OCT4 in the self-renewal phenotype. These results suggest that *OCT4*-transduced breast colonies (OTBCs) represent model cell lines to study self-renewal gene programs dysregulated in poorly differentiated breast tumors.

## Materials and methods

### Preparation of primary breast cells from fresh tissue

The first primary breast cell preparation was purchased from a commercially available source (lot 7F3286, abbreviated mammoplasty p86; Lonza, Walkersville, MD, USA). Subsequently, we generated three more primary cultures (p48, p52, and p78) derived from normal breast tissue procured from the Tissue Procurement Core Facility of the University of North Carolina Lineberger Comprehensive Cancer Center in accordance with approved institutional review board (IRB) protocols (Biomedical IRB, study 09-0777). Immediately after surgery, tissues were collected in transport medium composed of Dulbecco's modified Eagle's medium/F-12 (DMEM/F-12) and 10% fetal bovine serum (FBS) supplemented with antibiotics (penicillin, streptomycin, gentamycin, and amphotericin B). Next, the tissue was gently minced after removal of stromal matrix and fatty areas. The epithelial-containing tissue was enzymatically dissociated by using digestion medium containing the following reagents: Ham's F-12/DMEM (1:1) supplemented with 10% FBS, 5 g/mL insulin (Sigma-Aldrich, St. Louis, MO, USA), 0.5 g/mL hydrocortisone (StemCell Tech, Vancouver, BC, Canada), 10 ng/mL cholera toxin (Sigma-Aldrich), collagenase/hyaluronidase 10× (StemCell Tech), and antibiotics. For complete digestion, the samples were placed at 37°C for 16 to 18 hours with gentle rotation. Breast organoids were separated by differential centrifugation at 80 *g *for 30 seconds. The epithelial fraction was obtained by centrifugation at 160 *g *for 2 minutes. For transduction experiments, cells were used at first or second passage.

### Cell lines

Immortalized human dermal fibroblasts and the human neural teratoma cell line NT2 (American Type Culture Collection, Manassas, VA, USA) were grown in DMEM supplemented with 10% FBS and antibiotics. Breast cell preparations (parental lines) were grown in mammary epithelial basal media (MEBM) (Lonza) supplemented with bovine pituitary extract, hydrocortisone, insulin, human epithelial growth factor (hEGF), and antibiotics. OTBCs were grown in mammosphere medium, MEBM (Lonza) containing 1 ng/mL hEGF (BD Biosciences, San Diego, CA, USA), 1 μg/mL hydrocortisone (StemCell Tech), 10 μg/mL insulin (Sigma-Aldrich), 4 ng/mL heparin (StemCell Tech), B27 (Invitrogen Corporation, Carlsbad, CA, USA), and antibiotics. All cells were grown in 5% CO_2 _at 37°C in a humidified incubator.

### Lentivirus preparation and transduction of primary breast cells

Lentiviral particles were prepared from HEK 293T packaging cells. Briefly, plasmids containing pSinOCT4 (Addgene, Cambridge, MA, USA), Gagpol, VSVG, and RSV-REV were transfected in HEK 293T cells by using Lipofectamine and Plus Reagent cationic lipids (Invitrogen Corporation). An empty lentiviral vector was used as a negative control. Culture media containing viral particles were collected 36 hours after transfection and filtered through a 22-m filter. Virus was concentrated by ultra-centrifugation at 2.8 × 10^4 ^revolutions per minute for 2 hours at 4°C. Virus was resuspended in MEBM plus bullet kit culture media (Lonza) supplemented with 8 g/mL polybrene (Sigma-Aldrich). Primary breast cells were seeded 24 hours prior to transduction in 100-mm dishes at 1 × 10^5 ^cells per plate. To maximize transduction efficiency, primary breast cells were transduced four times in 48 hours.

### Derivation of *OCT4*-transduced breast cells

Inactivated mouse embryonic fibroblasts (MEFs) (C57BL/6; IRR #SCRC-1008; American Type Culture Collection) were seeded on 0.1% gelatin-coated six-well plates at a density of 1.5 × 10^4 ^cells/cm^2^. Immediately after the fourth transduction, breast cells were trypsinized and seeded on MEF-coated plates in hESC media [18]. Typically, control-transduced and non-transduced cells formed transient colonies that lasted 3 weeks. The OTBCs were highly proliferative and mesenchymal-appearing. OTBCs were picked between 21 and 28 days after seeding on MEFs and transferred to secondary and tertiary feeder cultures. After the third passage, colonies were mechanically dissociated and transferred to low-attachment plates (Corning, Corning, NY, USA) and mammosphere medium. OTBCs were maintained in self-renewal conditions as spheroids and were passaged every 5 days.

### Senescence assays

For β-galactosidase senescence assays, 5 × 10^4 ^OTBCs and parental lines were seeded in a six-well plate. Staining was done with the senescence β-galactosidase staining kit in accordance with the instructions of the manufacturer (Cell Signaling Technology, Inc., Danvers, MA, USA), β-galactosidase-positive cells from parental and OTBC lines were counted, and the average of four different fields was plotted.

### Quantitative real-time polymerase chain reaction

Total RNA from all cell lines was extracted by using the RNeasy extraction kit (Qiagen, Valencia, CA, USA) in accordance with the instructions of the manufacturer. Five grams of total RNA was reverse-transcribed by using the high-capacity cDNA archive kit (Applied Biosystems, now known as Life Technologies, Carlsbad, CA, USA) plus RNase inhibitor (Applied Biosystems). Gene transcription was quantified by qRT-PCR by using hydrolytic probes (Taqman; Applied Biosystems) or Absolute Blue QPCR SYBR low Rox mix (Thermo Scientific, Rockford, IL, USA). Fold change in gene expression for each sample and experimental condition was calculated as 2^Ct(control) - Ct(sample) ^± standard deviation. Primers and probes are listed in Table S1 in Additional file 1.

### Differentiation culture conditions

Cells were resuspended in 20 μL of Matrigel™ (BD Biosciences, San Diego, CA, USA). The Matrigel™ cell mixture was placed at the bottom of the well (eight-well Lab-Tek; Thermo Scientific, Rochester, NY, USA) and allowed to sit at 37°C for 30 minutes. The well was filled with 300 μL of differentiation medium: Ham's F-12 medium with 5% FBS, 5 μg/mL insulin (Sigma-Aldrich), 1 μg/mL hydrocortisone (StemCell Tech), 10 μg/mL cholera toxin (Sigma-Aldrich), 10 ng/mL epithelial growth factor (BD Biosciences), and 1 μg/mL prolactin (Sigma-Aldrich). Cells were cultured for 3 weeks in 5% CO_2_. Cells were fed with medium every other day. Cells were fixed with 4% paraformaldehyde and permeabilized with 0.3% triton-X100 before being processed for immunostaining [19].

### Differentiating culture for terminal ductal lobular unit assay

Cells were grown in three-dimensional (3D) basement membrane culture. Growth factor-reduced Matrigel™ (BD Biosciences) was combined in a 1:1 ratio with differentiation medium; 100 μL was added to each well of an eight-well glass slide chamber (Lab-Tek; Thermo Scientific) and allowed to solidify for 2 hours in a 37°C incubator. Cells were trypsinized, counted, and diluted to 500 cells per well. A 30% Matrigel™ solution was prepared in differentiation medium. The cell suspension was combined in a 1:1 ratio with the 30% Matrigel™ solution, and 200 μL of this mixture was added to each well. Prolactin was added to the media at a concentration of 1 μg/mL for the alveolar differentiation assays only. Cells were fed with differentiation medium containing 5% Matrigel™ every 4 days.

### Short interfering RNA target gene knockdown

OTBCs were reverse-transfected with 50 nM short interfering RNA (siRNA) smart pools (4 siRNAs per pool; Thermo Fisher Scientific, Lafayette, CO, USA), complexed with dharmaFECT reagent (Thermo Fisher Scientific). Transfection conditions were optimized by using a cytotoxic siRNA targeted against human ubiquitin B (Thermo Fisher Scientific). Spheroids or mammospheres from single cells were allowed to form in mammosphere medium as described above. For knockdown validation, cells were transfected in low-adherence six-well plates at 2.5 × 10^5 ^cells per well. Cells were left in transfection media for 48 hours for cell viability assays and 72 to 96 hours for Western blot analysis of target gene knockdown.

### Cell viability assays

Cells were reverse-transfected with siRNAs in 96-well low-adherence plates at 5 × 10^3 ^cells per well with four replicates. The Cell Titer Glo (CTG) assay (Promega Corporation, Madison, WI, USA) was used to determine the number of viable cells in low-adherence plates (mammosphere conditions) after siRNA transfection. Plates were allowed to cool down to room temperature, and 10 μL of CTG reagent was added per well and incubated for 15 minutes on an orbital shaker. Plates were assayed for luminescence in a plate reader (Pherastar, Ortenberg, Germany).

### Mouse tumor studies

Female athymic nude mice (3 weeks old) were purchased from Harlan Laboratories **} **(San Diego, CA, USA) for fat pad xenografts. The Institutional Animal Care and Use Committee at the University of North Carolina at Chapel Hill approved all of the following described experiments (protocol 08-311.0). Female nude/severe combined immunodeficient (nude/SCID) mice (3 weeks old) were purchased from Taconic Farms (Hudson, NY, USA) for subcutaneous xenografts and the spontaneous metastasis assays. Fat pad xenografts were generated with 1 × 10^5 ^OTBCs and a mixture of irradiated and non-irradiated immortalized fibroblasts (1:1, 1 × 10^5 ^cells total) in a final volume of 50 μL. The inguinal fat pad number four of 3-week-old mice was cleared and injected with the cell mix. Estrogen pellets were implanted subcutaneously at the time of the injection. All animals were euthanized when the tumors were approximately 1.2 cm in the largest length. Tumors were collected and processed for histology and inmmunohistochemistry. Subcutaneous xenografts were generated by injecting animals in the flank with 1 × 10^6 ^OTBCs diluted with Matrigel™ (1:1). Fluorescence imaging was performed with a highly sensitive cooled CCD (charge-coupled device) camera mounted in a light-tight specimen box (IVIS™; Xenogen, Taconic Farms (Hudson, NY, USA)). Signal quantification was obtained by Living Image (Xenogen, Taconic Farms (Hudson, NY, USA)) software. For *in vivo *imaging, animals were anesthetized (1% to 3% isoflurane) and imaged from dorsal and ventral sides once a week. Spontaneous metastases were evaluated by injecting four female nude/SCID mice (3 weeks old) with 1 × 10^5 ^OTBCs-86-L1-Ds-Red cells in 50 μL of sterile PBS in the left ventricle of the heart. Imaging of the metastatic lesions was performed *in vivo *once a week. For TIC experiments, animals were injected in the flank with OTBCs-86-L1-Ds-Red cells diluted to 1, 50, 1, 000, 100, 000, and 1, 000, 000 cells in a 100 μL PBS/Matrigel™ (1:1) mixture. Tumor growth was monitored by caliper measurement and fluorescence imaging as described above.

### Gene expression microarrays

A total of 11 cell lines were used for gene expression analyses: 4 parental cell lines (p48, p52, p78, and p86), 6 OTBCs (OTBCs48-L1, OTBCs52-L1, OTBCs78-L1, OTBCs86-L6, OTBCs86-L4, and OTBCs86-L1), and 1 OCT4 siRNA cell line (with 1 technical replicate). In addition, one tumor sample generated from the OTBCs86-L1 cell line was used for gene expression analysis. From each sample, total RNA was purified, amplified, labeled, and hybridized by using Agilent (Agilent Technologies, Santa Clara, CA, USA) 4 × 44 K oligo microarrays (platform GPL7504). All microarray data have been deposited in the Gene Expression Omnibus under accession number GEO:GSE26539. The probes/genes were filtered by requiring the lowest normalized intensity values to be greater than 10 in both samples and controls. The normalized log_2 _ratios (Cy5 sample/Cy3 control) of probes mapping to the same gene were averaged to generate independent expression estimates. We also used available microarrays from the UNC337 dataset [6]. For the UNC337 dataset, genes were median-centered, and samples were standardized to zero mean and unit variance before other analyses were performed. All microarray cluster analyses were displayed by using Java Treeview version 1.1.3. Average-linkage hierarchical clustering was performed by using Cluster version 2.12 [20]. Analysis-of-variance tests for gene expression data were performed using R.

### *OCT4*-transduced breast cell gene signatures

To build an OTBC signature, we first selected those genes that were significantly and differentially expressed between six OTBCs (OTBCs48-L1, OTBCs52-L1, OTBCs78-L1, OTBCs86-L6, OTBCs86-L4, and OTBCs86-L1) and their four respective parental cell lines (p48, p52, p78, and p86) by using two-class paired SAMs (significance analysis of microarrays) and a less than 1% false-discovery rate. The resulting upregulated (*n *= 534) and downregulated (*n *= 1, 144) gene lists are shown in supplemental data (Tables S4 and S5, respectively). To estimate the expression of the OTBC signature across the intrinsic molecular subtypes of breast cancer, we calculated the mean expression of both gene lists (that is, up- and downregulated) in the entire median-centered UNC337 dataset (*n *= 327) by using the subtype calls described in [6]. Among the entire gene list of the OTBC signature (*n *= 1, 678), only three genes were found missing in the UNC337 dataset.

Immunofluorescence, flow cytometry, Western blotting, immunohistochemistry, and small-molecule epigenetic inhibitor treatments are described in supplementary methods in Additional file 2. Primary and secondary antibodies were used in accordance with the recommendations of the manufacturer and are listed in Table S2 in Additional file 3.

## Results

### Isolation of *OCT4*-overexpressing clones from normal breast preparations with persistent self-renewal ability

**Figure 1:**
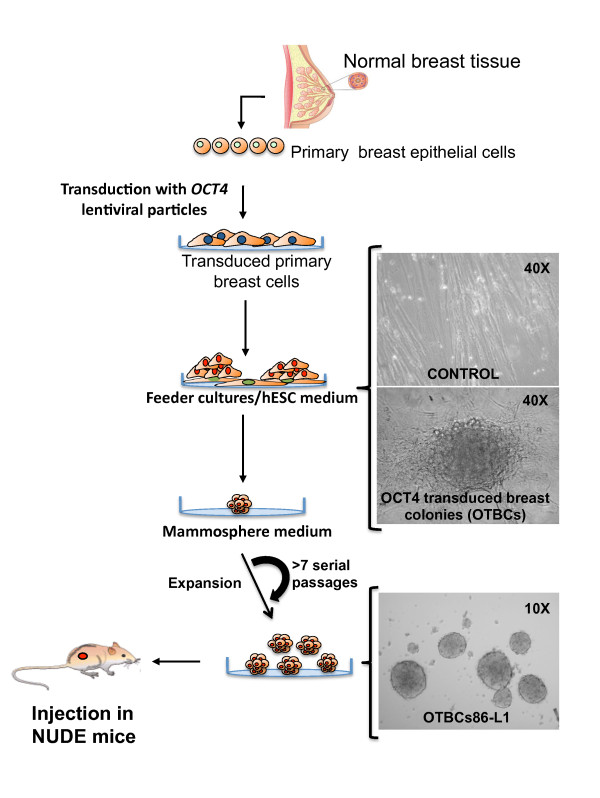
**Generation of *OCT4*-transduced breast cells (OTBCs) from primary cultures of mammary cells**. Graphical representation of the method used to generate OTBCs from normal breast tissue. Representative images of control cells (empty lentiviral vector) and OTBCs are shown 2 weeks after transduction of *OCT4 *and seeding in feeder cultures of mouse embryonic fibroblasts in human embryonic stem cell (hESC) media. The OTBCs-86L1 image is a representative example of cells (passage 25) grown in suspension in mammosphere media after cell expansion.

To isolate clonal populations of *OCT4*-overexpressing cells with sustained self-renewal ability, approximately 1 × 10^5 ^human mammary epithelial cells were lentivirally transduced and seeded in feeder cultures of irradiated fibroblasts in hESC media, conditions known to facilitate the expansion of adult and embryonic stem cells (Figure 1). Control and mock-infected cells generated transient colonies that survived approximately 2 to 3 weeks. However, *OCT4 *cDNA transduction produced 1 to 10 colonies per 1 × 10^5 ^transduced cells. In contrast to control colonies, these *OCT4*-transduced breast colonies (abbreviated as OTBCs) were visible 2 weeks after seeding in MEF conditions (Figure 1). OTBCs appeared non-encapsulated in feeder cultures and exhibited a distinctive transparent mesenchymal-like morphology with multiple visible secretion vesicles. These colonies could be propagated in secondary feeder cultures and thus sustained self-renewal ability. We recovered a total of six OTBCs from the commercially available source (mammoplasty p86), two colonies from p48, two colonies from p52, and five colonies from p78. In all cases, no colonies were recovered in control cells (un-transduced cells or cells transduced with an empty vector). To maintain the OTBCs in self-renewal conditions in feeder-free cultures, colonies from secondary passages of feeder cultures were then propagated in mammosphere media in low non-adherent plates, which are culture conditions known to expand mammary stem and progenitor cells [8]. Because of technical limitations in obtaining a sufficient number of cells for biological assays, we chose the six OTBCs derived from p86 and one line derived from p48 for future studies. These seven lines displayed the highest proliferation rates among all the clones analyzed. Unlike the parental primary breast preparations used for the transductions, which could not be further propagated for more than three to seven serial passages in spheroid conditions (mammospheres), these OTBCs could be propagated for more than 40 passages. Importantly, identical mesenchymal colonies could be isolated when the transduced cells were seeded in feeder-free conditions (cells grown in gelatin-coated plates in MEF-conditioned media), indicating that the isolated OTBCs were not a contamination from the feeder cultures. Overall, these observations suggested that a rare population of epithelial cells (up to 0.01% of mammary epithelial cells) could be immortalized and infinitely propagated in self-renewal spheroids by expression of *OCT4 *cDNA.

### *OCT4*-transduced breast cells defeat the cellular senescence process

**Figure 2:**
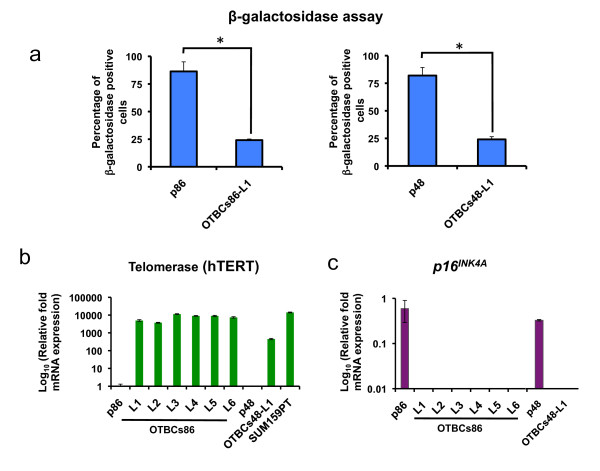
***OCT4*-transduced breast cells (OTBCs) underwent an immortalization process**. **(a) **OTBCs overcame cellular senescence. The histogram quantifies β-galactosidase-positive cells in the OTBCs-86L1 and OTBCs-48L1 lines. Data were normalized to the parental cell lines p86 and p48 and represent the mean ± standard deviation (SD) of three independent experiments (*P ≤0.01). **(b) **Telomerase mRNA levels are upregulated in OTBCs relative to the parental p86 and p48 lines. The SUM159PT breast cancer cell line was used as a positive telomerase gene expression control. Samples were normalized to the parental p86 and p48 lines. Bar graphs represent the mean ± SD of three independent experiments. **(c) **OTBCs downregulate *p16^Ink4a ^*mRNA levels as assessed by quantitative real-time polymerase chain reaction. The *p16^Ink4a ^*levels in OTBCs were normalized to the parental lines. Bar graphs represent the mean ± SD of three independent experiments. *hTERT*, human telomerase reverse transcriptase.

We first measured β-galactosidase activity, which is a marker of cellular senescence, in both the parental lines and their derived OTBCs. Most of the cells in the parental lines at passage seven stained strongly positive for β-galactosidase, whereas only a few cells in the OTBCs were positive in the same assay, suggesting that OTBCs bypassed the cellular senescence process (Figure 2a). Both shortening of telomeres and proteins regulating cell cycle, such as p16^INK4A^, are known to play an important role in cell aging [21,22]. The maintenance of telomere ends by the telomerase reverse transcriptase (hTERT) and the downregulation of regulator p16^INK4A ^are two of the mechanisms for the self-renewal of stem cells [23]. Therefore, we next examined *hTERT *and *p16^INK4A ^*mRNA levels by qRT-PCR in OTBCs and parental lines. As shown in Figure 2b, OTBCs upregulated *hTERT *mRNA levels by more than 1, 000-fold relative to the parental lines. The *hTERT *levels in OTBCs were similar to those of the SUM159PT breast cancer cell line [1]. In contrast to *hTERT *levels, *p16^INK4A ^*mRNA levels were completely downregulated in OTBCs when compared with their respective parental lines (Figure 2c). These results indicated that OTBCs overcame cellular senescence and underwent an immortalization process.

### OCT4-transduced breast cells maintain aberrant self-renewal and are able differentiate into breast epithelial cell lineages

**Figure 3:**
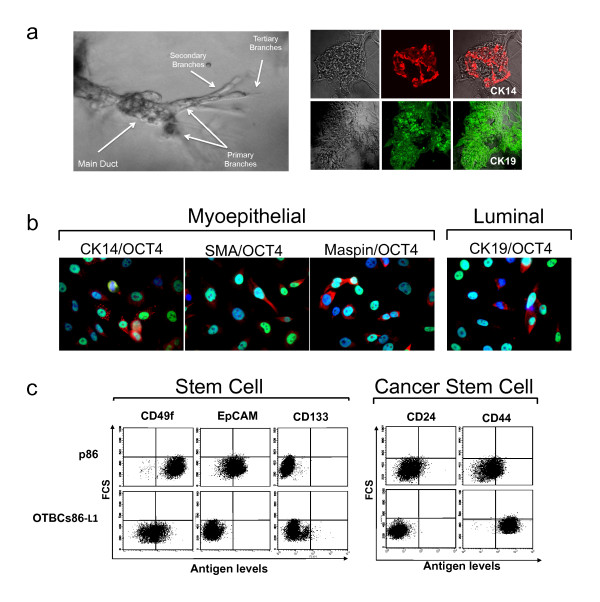
**Breast cancer stem cell-like properties of OCT4-transduced breast cells (OTBCs) *in vitro***. **(a) **Left: Formation of terminal ductal lobular units in three-dimensional (3D) cultures in the presence of Matrigel™ and prolactin. OTBCs were seeded in a 3D culture; 3 weeks later, primary, secondary, and tertiary branching structures were visible. Right: Immunostaining of 3D structures with anti-CK19 (luminal, green) and anti-CK14 (myoepithelial, red) antibodies. **(b) **Immunostaining of myoepithelial and luminal markers in OTBCs86-L1 cells. Cells were stained for OCT4 (green) and CK14, SMA, Maspin, or CK19 (red) markers. **(c) **Flow cytometry analysis of antigenic phenotypes characteristic of bi-potent mammary stem cells (CD133^low^CD49f^+^EpCAM^-^) and cancer stem cells (CD44^+^CD24^-^) in the parental line p86 and the clone OTBCs86-L1. The forward scatter channel was plotted in the y-axis, and the fluorescence of the cell surface antigens was plotted in the x-axis. Virtually identical flow cytometry results were obtained from all analyzed clones (Figure S2 in Additional file 5). Experiments were repeated at least three times with similar results. Data shown are representative of one experiment. EpCAM, epithelial cell adhesion molecule.

We next examined the phenotypic properties of the cell of origin of OTBCs by performing differentiation assays. When single-cell suspensions of OTBCs were placed in 3D cultures of Matrigel™ and prolactin, terminal ductal lobular-like units (TDLUs) were formed with primary, secondary, and tertiary branching structures (Figure 3a). These TDLU-like structures were very similar morphologically to those reported for bi-potent stem cells and cancer stem cell lines [24]. The formation of these structures suggested that the cell of origin that gave rise to OTBCs was possibly a primitive stem cell or early progenitor cell. To verify this, we assessed the differentiation potential of OTBCs by seeding single-cell suspensions of OTBCs in fibronectin-coated wells for 4 days. Lineage specification was followed by immunofluorescence by using specific antibodies. As shown in Figure 3b, all OTBCs analyzed were able to generate myoepithelial-specific (CK14^+^, SMA^+^, and Maspin^+^) cell populations. However, only a population of cells within the colony (usually appearing as clusters of cells possibly arising from a single cell) was able to differentiate, and most cells still maintained strong OCT4 nuclear staining. In addition to myoepithelial-positive cells, some OTBCs generated luminal-specific cell populations, such as cytokeratin (CK) 19^+ ^cells and epithelial cell adhesion molecule-positive (EpCAM^+^) cells (Figure S1 in Additional file 4). CK14^+ ^and CK19^+ ^colonies were detected when OTBCs were allowed to differentiate in Matrigel™ (Figure 3a, right panel). The fact that only a relatively small population of cells was able to differentiate and that OCT4 was still highly expressed in OTBCs suggested that these cells maintained aberrant self-renewal and were limited from undergoing downstream differentiation gene programs. We concluded, on the basis of these differentiation assays *in vitro*, that OCT4 transformed a stem or early progenitor cell. Some OTBCs (for example, OTBCs86-L1) were bi-potent cells, able to generate both lineages, whereas other OTBCs (for example, OTBCs78-L1) appeared to be myoepithelial-restricted. Nevertheless, the fact that all OTBCs were able to give rise to CK^+ ^cells was consistent with an epithelial origin and excluded the possibility that these cells were generated from minor stromal contaminants present in the epithelial preparations.

### Stem and tumor-initiating cell-like antigenic properties of OCT4-transduced breast cells *in vitro*

We first investigated the antigenic signatures of OTBCs and their parental lines by flow cytometry by using cell surface marker panels used to identify prospective breast stem and progenitor cells. As shown in Figure 3c and in Figure S2 in Additional file 5, all OTBCs were EpCAM^-^, CD49f^+^, and CD133^low^. These markers are consistent with reported signatures characterizing putative stem/progenitor cells of the breast [8]. Levels of CD49f were more variable among OTBCs, but all clones consistently stained positive for this marker (Figure S2 in Additional file 5). The finding that all OTBCs were EpCAM^- ^suggest that the cell of origin of OTBCs is possibly not a luminal-restricted progenitor but rather a breast stem cell, a bi-potent progenitor, or a myoepithelial-restricted progenitor cell, and this is in agreement with the results of our differentiation assays. Next, we examined the prevalence of the CD44^high^/CD24^- ^signature, which has been used to isolate prospective breast TIC populations in tumor specimens and cell lines [2,5]. As shown in Figure 3c and in Figure S2 in Additional file 5, all of the OTBCs analyzed acquired the tumorigenic, TIC-like signature, CD44^high^/CD24^-^.

### Tumorigenic capabilities of OCT4-transduced breast cells in immunodeficient mice

The aberrant self-renewal ability of OTBCs and the prevalence of the CD44^high^/CD24^- ^TIC signature in all of the OTBCs suggested that these cell lines could have tumorigenic potential *in vivo*. High CD44^high^/CD24^- ^ratios have been associated with the claudin-low breast cancer subtype [6,11]. To explore the potential of OTBCs to generate tumors, we first developed orthotopic models. Cells (1 × 10^5^) from OTBCs86-L1 were injected in the fat pad of nude mice in the presence of human fibroblasts (Table S3 in Additional file 6), which are commonly used to support the growth of mammary stem cells and other TIC lines [25]. We additionally injected 1 × 10^5 ^cells from OTBCs86-L1 in the absence of fibroblasts with Matrigel™ in the flank of nude mice (Table S3 in Additional file 6). We found that the fat pad injection in the presence of stromal fibroblasts highly facilitated the growth of these cells and that all of the animals developed fast-growing tumors in less than 2 weeks after injection. The same cells injected subcutaneously in the absence of fibroblasts developed tumors at day 16 after injection. We next performed a cell dilution experiment to address whether OTBCs acquired tumor-initiating potential. As shown in Table 1, 50 cells from OTBC-86-L1 were sufficient to generate subcutaneous tumors in five out of eight injected animals. Thus, these results indicated that OTBCs acquired tumor-initiating capabilities.

**Table 1:** Tumor-initiating cell capabilities of OTBCs86-L1

Cell line	Number of cells injected subcutaneously	Number of tumors	Days
OTBCs86-L1-DsRed	1, 000, 000	9/9	9
	100, 000	6/6	16
	1, 000	8/8	25
	50	5/8	45
	1	0	> 90

**Figure 4:**
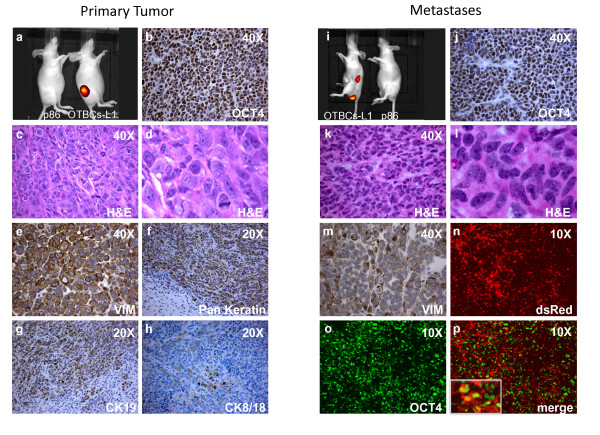
***OCT4*-transduced breast cells (OTBCs) generate primary tumors and experimental metastases upon injection in immunodeficient mice**. **(a) **Representative image of a prototype subcutaneous primary tumor generated by OTBCs86-L1-DsRed cells. Control mice were injected with the parental p86 line. Animals were injected with 1 × 10^6 ^cells; for simplicity, only one animal is shown. The OTBCs86-L1-DsRed was engineered with a lentiviral vector expressing the DsRed gene, which facilitated tumor measurement by fluorescence imaging (IVIS-kinetic; Xenogen). Shown is a representative paraffin-embedded tumor section stained with **(b) **OCT4 (40×), **(c) **hematoxylin and eosin (H&E) (40×), **(d) **H&E magnification from (c), **(e) **vimentin (VIM) (20×), **(f) **pan-keratin (AE1/AE3, 20×), **(g) **keratin 19 (20×), and **(h) **keratin 8/18 (20×). **(i) **Representative image of spontaneous metastatic lesions. Immunodeficient mice were injected in the left ventricle of the heart with OTBCs86-L1-DsRed cells, and imaging was performed at day 90 after injection. Shown is a representative paraffin-embedded tumor section stained with **(j) **OCT4 (40×), **(k) **H&E (40×), **(l) **H&E magnification from (k), and **(m) **VIM (40×). Immunofluorescence detection of **(n) **DsRed-labeled OTBCs (10×), **(o) **OCT4 (20×), and **(p) **merged image of DsRed and OCT4 (10×). The square on the left corner shows a detail of DsRed-positive tumor cells expressing OCT4.

To image these tumors *in vivo*, non-invasive fluorescence imaging was performed by using OTBC-86-L1 cells expressing DsRed (Figure 4a, i). Immunohistological examination of these primary tumors revealed histological features reminiscent of high-grade, poorly differentiated breast carcinomas. The majority of primary tumor cells retained high OCT4 nuclear expression (Figure 4b) and comprised high-grade atypical cells with high nuclear-to-cytoplasmic ratio, prominent nucleoli and a high mitotic index, the last of which is another hallmark of poorly differentiated human breast cancers [13] (Figure 4c, d). The vast majority of subcutaneous and orthotopic tumors were strongly positive for the mesenchymal marker vimentin (VIM) (Figure 4e). Pathological examination of the tumors suggested that OTBCs generated poorly differentiated epithelial breast carcinomas, which were negative for PR, ER, and HER2. Importantly, a subset of tumor cells stained positive for CKs, such as CK19 (Figure 4g), CK8/CK18 (Figure 4h), and pan-keratin (AE1/AE3) (Figure 4f). In conclusion, analysis of tumor pathology supports the classification of these tumors as carcinoma of epithelial origin.

To determine the metastatic potential of OTBCs, OTBC-86-L1-DsRed cells were injected in the left heart ventricle of nude mice (Table S3 in Additional file 6). The red fluorescent protein allowed the detection of metastatic lesions by using a Xenogen fluorescence-imaging camera in living animals (Figure 4i) and in tumor sections (Figure 4n). Metastases were evident in two out of four animals 2 months after injection, with multiple lesions, including ovarian metastases (Figure 4i-p). The immunohistochemical analysis of the metastases revealed poorly differentiated high-grade tumor cells (Figure 4k, l) and strong OCT4 staining in most of the cells (Figure 4o) and weak positive staining for VIM (Figure 4m), and this was similar to what was observed in primary tumors.

Overall, these *in vivo *assays demonstrated that OTBCs were able to generate subcutaneous and orthotopic tumors that were reminiscent of high-grade, triple-negative, and poorly differentiated breast carcinomas. Similar tumors were obtained with independent injection of three additional OTBC clones (see Table S3 in Additional file 6 for all mouse experiments). Collectively, our data show that the OTBC lines acquired TIC properties.

### OCT4-transduced breast cells exhibit a loss of epithelial and gain of mesenchymal markers

**Figure 5:**
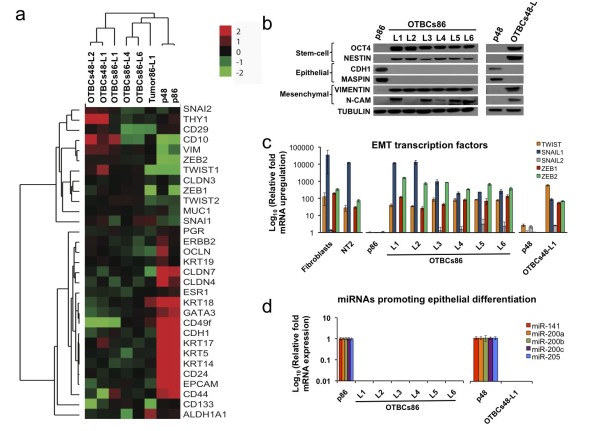
***OCT4*-transduced breast cells (OTBCs) exhibit an epithelial-to-mesenchymal transition (EMT) gene signature**. **(a) **Microarray expression analysis of genes selected across parental (p48 and p86), OTBC lines (OTBCs48-L1 and L2, OTBCs86-L1, L4, and L6), and a subcutaneous tumor derived from OTBCs86-L1 (tumor 86-L1). Array trees were derived by unsupervised hierarchical clustering using markers of EMT, basal, luminal stem cell, cancer stem cell, and epithelial differentiation. Each colored square on the upper right represents the relative mean transcript abundance (in log_2 _space); highest expression is red, average expression is black, and lowest expression is green. **(b) **Western blot detection of molecular markers in the parental lines (p86 and p48), OTBCs86-L1 through L6, and OTBCs48-L1. The following markers were analyzed: OCT4, NESTIN, CDH1, Maspin, vimentin, and N-CAM. Tubulin was used as a loading control. Results are representative examples of three independent experiments. **(c) **Detection of EMT transcription factor expression by quantitative real-time polymerase chain reaction (qRT-PCR). Gene expression levels were normalized to those of the parental cell lines (p86 and p48). Control cell lines used for gene expression analyses include human dermal fibroblasts and the human teratoma cell line NT2. **(d) **MicroRNAs (miRNAs) known to promote epithelial differentiation are detected by qRT-PCR. Levels of miRNA expression in OTBCs of miR-141, miR-200a, miR-200b, miR-200c, and miR-205 were normalized to those of the parental lines (p86 and p48). Analysis was performed by using miR-U6 as an internal control. Bar graphs represent the mean ± standard deviation of three independent experiments. CDH1, E-cadherin; N-CAM, neural cell adhesion molecule.

To gain mechanistic insight into how OTBCs developed aberrant self-renewal and gain of TIC features, we investigated the molecular targets of OCT4. We performed gene expression microarray analysis on four parental normal breast preparations (designed as p52, p78, p86, and p48 in Figure 5a) and their corresponding OTBC-derived lines (three OTBC lines derived from p86 and one line from each of the remaining parental lines). The genome-wide transcriptional analysis revealed that all OTBCs maintained a poorly differentiated state as reflected by the weak expression of epithelial markers (CKs 5, 14, 17, 18, and 19), loss of TFs specifying lineage commitment such as *GATA3*, and the concomitant gain of self-renewal TFs, such as *OCT4 *and *NANOG*. Furthermore, all OTBC lines examined exhibited a complete loss of epithelial junction markers, such as *E-cadherin *and members of the claudin gene family, and a gain of mesenchymal markers, such as VIM. Interestingly, a sample derived from a subcutaneous tumor injected with OTBCsp86-L1 (tumor 86-L1) (Figure 5a) revealed a small reactivation of CKs relative to the original OTBC line used to generate the tumor. This finding supports the histological data of Figure 4, suggesting that possibly only a fraction of cells within the tumors are able to differentiate in CK^+ ^cells.

The gain of stem cell markers and loss of epithelial proteins were also validated by Western blot analysis and qRT-PCR (Figure 5b, c). OTBCs retained high expression of the stem cell marker Nestin, which has been shown to be overexpressed in isolated mammary stem cells [8,26] (Figure 5b). All OTBCs exhibited a complete loss of epithelial markers associated with tumor suppressive functions, such as E-cadherin (CDH1) and Maspin, and a gain of mesenchymal markers, such as VIM and neural cell adhesion molecule (N-CAM). N-CAM is essential for EMT induction [27] and for the maintenance of a mesenchymal state [28]. Other TFs known to facilitate EMT, such as *TWIST*, *SNAIL1*, *SNAIL2*, *ZEB1*, and *ZEB2*, were upregulated in OTBCs (Figure 5c). Furthermore, microRNAs that promote epithelial differentiation by targeting the EMT TFs ZEB1/2 (members of the miR-200 family [29,30] and miR-205 [31]) were all downregulated in OTBCs (Figure 5d). Overall, these results demonstrate that OTBCs maintained stem cell/progenitor characteristics and gained mesenchymal markers relative to their parental lines.

### *OCT4*-transduced breast cells resemble the claudin-low molecular subtype of breast cancer

**Figure 6:**
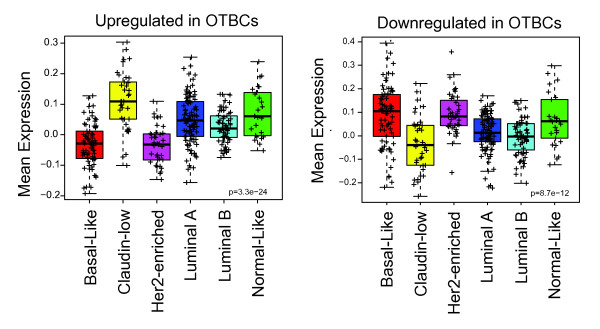
***OCT4*-transduced breast cells (OTBCs) up- and downregulate genes clustered with the claudin-low molecular subtype of breast cancer**. Box-and-whisker plot for the mean expression of the upregulated (534 genes) and downregulated (1, 144 genes) gene signatures (Tables S4 and S5 in Additional files 7 and 8, respectively) in OTBCs compared with the parental cell lines across the intrinsic molecular subtypes of breast cancer by using the published breast cancer patient database (UNC337) [6]. *P *values have been calculated by comparing gene expression means across all subtypes. *HER2*, epidermal growth factor receptor 2.

A hallmark of the claudin-low subtype of breast cancer is the enrichment of mesenchymal markers along with the downregulation of epithelial junction proteins, including E-cadherin and claudins [6]. Indeed, EMT has been associated with stemness and mammary gland tumorigenesis [12]. We next examined overlapping gene signatures between OTBCs and claudin-low carcinomas. OTBCs from four different mammoplasty donors revealed very similar genome-wide transcriptional profiles, which facilitated the generation of two robust signatures of genes significantly up- and downregulated, respectively, in all OTBCs samples relative to their parental lines. These upregulated (*n *= 534) and downregulated (*n *= 1, 144) gene signatures were examined across the intrinsic molecular subtypes of breast cancers by using a published cohort of 337 samples [6]. Our analysis shows that the up- and downregulated gene signatures were significantly over-represented or under-represented in the claudin-low subtype, respectively (Figure 6; Tables S4 and S5 in Additional files 7 and 8). Thus, our genome-wide analysis supported the finding that OCT4 overexpression in OTBCs strongly correlated with a subset of breast carcinomas enriched in cancer stem cell gene signatures and mesenchymal markers.

### Activation of targets of NANOG, OCT4, and SOX2 in *OCT4*-transduced breast cells

**Figure 7:**
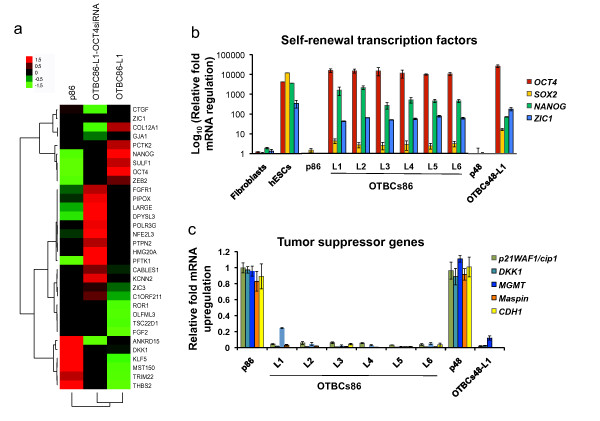
**Differential regulation of the human embryonic targets of NANOG, OCT4, and SOX2 (NOS targets) in *OCT4*-transduced breast cells (OTBCs)**. **(a) **Expression of selected NOS targets across the parental cell line p86, the OTBCs86-L1 cell line, and the same line OTBCs86-L1 transfected with a short interfering RNA specific for *OCT4*. Efficient knockdown of OCT4 was validated by Western blot. Each colored square on the left panels represents the mean relative transcript abundance (in log_2 _space); highest expression is red, average expression is black, and lowest expression is green. Data represent the average of three independent experiments. **(b) **Detection of self-renewal transcription factors *OCT4*, *NANOG*, *SOX2*, and *ZIC1 *expression by quantitative real-time polymerase chain reaction (qRT-PCR). Control cell lines used for gene expression analyses include human dermal fibroblasts and human embryonic stem cells (hESCs). The mRNA levels were normalized to those of the parental cell lines (p86 and p48). Bar graph represents the mean ± standard deviation (SD) of three independent experiments. **(c) **Detection of tumor suppressor gene expression by qRT-PCR. Genes analyzed include *p21WAF1/cip1*, *Dickkopf-related protein 1 *(*DKK1*), *methylguanine-DNA methyltransferase *(*MGMT*), *Maspin (SERPINB5*), and *E-cadherin *(*CDH1*). The mRNA levels were normalized to those of the parental cell lines (p86 and p48). Bar graph represents the mean ± SD of three independent experiments. NOS, NANOG, OCT4, and SOX2 targets.

To investigate the molecular mechanisms mediating the tumor-initiating capabilities of OTBCs, we examined the expression of OCT4 and its downstream targets by gene expression microarrays and qRT-PCR. In hESCs, OCT4, SOX2, and NANOG TFs comprise the core of an autoregulatory feedback loop that activates self-renewal and inhibits differentiation gene programs [32]. Common targets of NANOG, OCT4, and SOX2 have been characterized by ChIP-chip and ChIP-seq in hESCs and mouse ESCs [32,33]. In hESCs, these TFs co-occupy and co-regulate a subset of 179 targets (known as the NANOG, OCT4, and SOX2 (NOS) signature [13]). Our gene expression microarrays revealed that multiple hESC NOS targets were differentially regulated in the OTBCs relative to the parental lines (Figure 7a). Furthermore, the expression of these targets was significantly perturbed in OTBCs depleted of OCT4 by RNAi-mediated knockdown (Figure 7a). These results suggested that OTBCs regulated direct embryonic targets of OCT4.

Interestingly, NOS targets are found over-represented in poorly differentiated breast cancers and gliomas [13]. As expected, our microarray analysis has shown that *OCT4 *mRNA was particularly enriched in the claudin-low and basal-like intrinsic subtype of breast cancers and also shows some expression in normal-like cancers (Figure S3 in Additional file 9). Consistently, NOS targets are also over-represented in the same subtypes (Figure S4 in Additional file 10).

### Upregulation of self-renewal transcription factors

NOS targets differentially upregulated in OTBCs relative to the parental lines comprised multiple self-renewal TFs. Of particular interest were *OCT4*, *SOX2*, *NANOG*, and the EMT TFs *ZEB*1 and *ZEB2*, which are transcriptional repressors of E-cadherin. Importantly, the endogenous levels of expression of *OCT4 *in OTBCs were comparable to or even higher than those detected in hESCs grown in self-renewal conditions. However, *SOX2 *levels in OTBCs were lower than those observed in hESCs. The downstream embryonic target of OCT4 *NANOG*, which is known to block differentiation gene programs in hESCs, was found partially reactivated in all of the OTBCs. In addition, we found that the NOS target gene *ZIC1 *was differentially regulated in all of the OTBC lines (Figure 7b). ZIC1 is a zinc finger TF expressed in hESCs [32] and has been shown to be necessary for the maintenance of the self-renewal phenotype in neural progenitors [34]. Furthermore, our upregulated gene signature (*n *= 534 genes) was enriched in TFs, particularly embryonic targets of *OCT4 *that specify pattern formation, such as homeobox-containing proteins (for example, the *SIX1 *TF) (Table S4 in Additional file 7). Whereas homeobox TFs specifying differentiation gene programs are repressed in hESCs, these targets were found upregulated in OTBCs. Thus, our analysis indicated that embryonic TF targets of OCT4 are upregulated in OTBCs. Importantly, we found that OCT4 targets exhibited different expression patterns in OTBCs relative to hESCs.

### Downregulation of tumor suppressor genes

**Figure 8:**
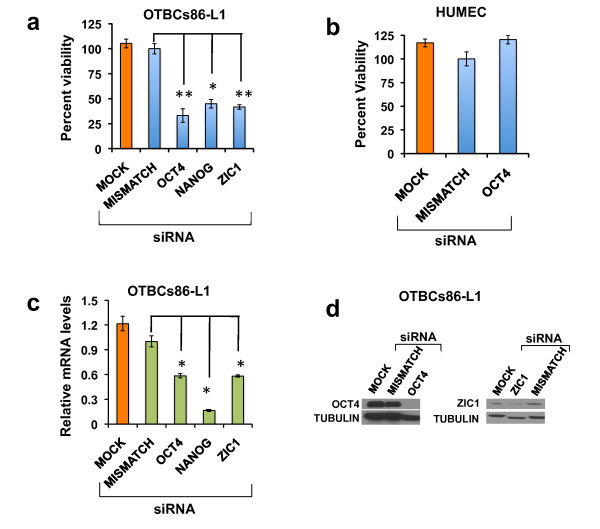
**Knockdown of OCT4, NANOG, and ZIC1 in *OCT4*-transduced breast cells (OTBCs) affects self-renewal**. **(a) **Cell viability was assessed in OTBCs86-L1 cells upon transfection of short interfering RNAs (siRNAs) designed to knock down *OCT4*, *NANOG*, and ZIC1. Data were normalized to cells transfected with a mismatch siRNA pool. Mock refers to untransfected OTBCs86-L1 cells. Cells were transfected with the corresponding siRNAs and placed in self-renewal conditions for 72 hours. Data represent the mean ± standard deviation (SD) of three independent experiments and were analyzed with student *t *test with *P *values set at *P ≤0.01 and **P ≤0.001. **(b) **Knockdown of OCT4 in immortalized human mammary epithelial cells (HUMECs). Transfection of siRNAs was performed as described above. **(c) **Real-time polymerase chain reaction quantification of *OCT4*, *NANOG*, and *ZIC1 *mRNA expression after knockdown in OTBCs86-L1 cells; data were normalized to cells transfected with a mismatch siRNA pool. **(d) **Protein expression levels of OCT4 and ZIC1 assessed by Western blot in OTBCs86-L1 (MOCK), knockdown OTBCs86-L1 (OCT4 or ZIC1), and mismatch siRNA pool cell population (MISMATCH). One of three independent experiments is shown.

NOS targets differentially downregulated in OTBCs relative to the parental lines comprised tumor suppressor genes, including *DKK1*, an antagonist of the Wnt signaling pathway. *DKK1 *is an NOS target abundantly expressed in hESCs [32]. In contrast, we found that *DKK1 *was downregulated in all OTBC lines. Indeed, *DKK1 *has been shown to be a secreted tumor suppressor in multiple breast cancer cell lines [35,36] and is epigenetically silenced in some breast cancer cell lines and primary tumors [37]. Similarly, multiple tumor suppressor genes known to be methylated in breast cancer, such as *Maspin *[38], *CDH1 *[39], *MGMT *[40], and *p21^WAF1/Cip1 ^*[41], were downregulated in OTBCs relative to the parental lines (Figure 8c).

We next investigated whether epigenetic mechanisms could account for the silencing of tumor suppressors in OTBCs. Tumor suppressor gene reactivation was examined in OTBCs treated with the methyltransferase inhibitor 5-aza-2'-deoxycytidine (5-aza-2'dC) or the histone deacetylase inhibitors (HDACis) suberoylanilide hydroxamic acid and trichostatin-A. As shown in Figure S5 in Additional file 11, *Maspin *and *CDH1 *were both reactivated upon treatment with 5-aza-2'dC as well as HDACis, whereas *DKK1 *and *MGMT *were significantly reactivated upon treatment with HDACis only. These results suggested that epigenetic silencing mechanisms involving histone methylation and de-acetylation could be responsible for the inactivation of a variety of tumor suppressors in OTBCs.

### RNA interference-mediated knockdown of self-renewal NOS targets in OCT4-transduced breast cells

The role of OCT4 and potential oncogenic targets of OCT4 in mediating the self-renewal phenotype in OTBCs was investigated by loss-of-function experiments. OTBCs were transfected with siRNAs specific for OCT4 and the OCT4 targets NANOG and ZIC1 (Figure 8). siRNA-transfected cells were allowed to form spheroids in a tumorsphere formation assay. The viability of the resulting tumorspheres was monitored by a Cell Titer Glo (CTG) assay, which measures cell viability by the release of ATP as a luminescent signal. As expected, the knockdown of *OCT4 *had the strongest effect in reducing the ability of OTBCs to form spheroids (Figure 8a). This drastic downregulation of cell viability promoted by OCT4 knockdown was observed only in OTBCs; no effect was seen in immortalized mammary epithelial cells, which do not express *OCT4 *(Figure 8b). This experiment demonstrates the pivotal role of OCT4 in maintaining the self-renewal characteristics of these cells. Likewise, siRNA-mediated knockdown of NANOG and ZIC1 significantly suppressed spheroid formation (Figure 8a-c). Collectively, our data suggest that OTBCs could be used as a claudin-low breast cancer model to potentially identify novel therapeutic targets.

**Figure 9:**
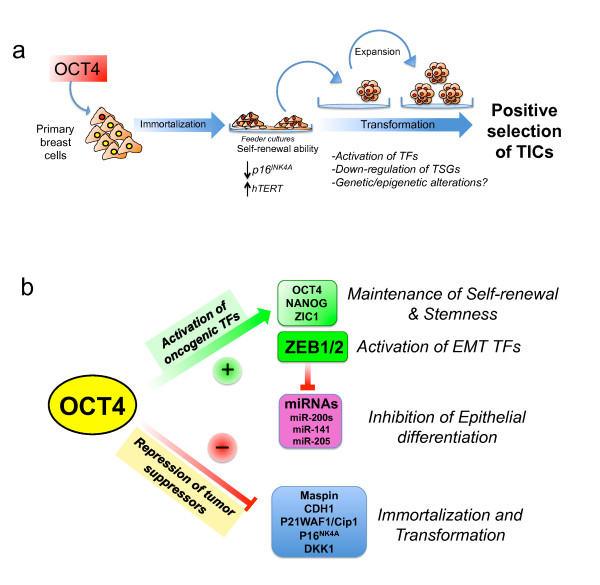
**Graphical model of the generation of tumor-initiating cells (TICs) and the OCT4 molecular targets regulated in *OCT4*-transduced breast cells (OTBCs)**. **(a) **Illustration of the proposed model for the generation of OTBCs endowed with tumor-initiating ability. **(b) **Endogenous expression of *OCT4 *in normal breast tissue results in aberrant self-renewal and expansion of an undifferentiated early stem/progenitor population. OCT4 expression results in an activation of self-renewal transcription factors (TFs) and a concomitant repression of tumor suppressor genes that allow cells to immortalize and gain stem cell-like features. CDH1, E-cadherin; EMT, epithelial-to-mesenchymal transition; hTERT, human telomerase reverse transcriptase; miRNA, microRNA; TSG, tumor suppressor gene.

A putative model summarizing the above molecular events is integrated in Figure 9. Our data suggest that a rare subpopulation of cells (approximately 0.01% to 0.1%) within the human mammary epithelial cell population is a target of OCT4. Overexpression of *OCT4 *cDNA resulted in a subpopulation of cells that activated self-renewal gene programs. These cells generated mesenchymal-appearing colonies on feeder cultures and could be propagated in feeder-free (mammosphere) conditions. Serial expansion of spheroids in multiple (more than seven passages) mediated the progressive selection for TIC-like cells (Figure 9a). At the molecular level, we propose that OTBCs gained and sustained self-renewal by activation of a TF network involving the embryonic targets of OCT4, such as NANOG, ZIC1, and EMT TFs (Figure 9b). Activation of EMT TFs was accompanied by the suppression of miRNAs involved in epithelial differentiation. Concomitantly with this activation of potential oncogenic TFs, tumor suppressor gene panels were found downregulated in OTBCs. A compromised tumor suppressor repertoire could result in the subsequent selection of clones possessing tumorigenic ability.

## Discussion

The isolation and characterization of TICs from human tumors and cell lines have been limited because these cells represent a rare population of cells within the tumor and also because of our lack of understanding of their molecular signatures. In this paper, we have described the isolation of TIC-like cells by exogenous expression of the *OCT4 *TF in primary breast cell preparations. We have also shown that OTBCs exhibit an overlapping gene signature with claudin-low carcinomas.

The relatively low (0.1% to 0.01%) frequency of mesenchymal colonies in the transduced samples suggests that a subpopulation of cells is the target of OCT4. It is possible that, in addition to inducing an expansion of a relatively undifferentiated and rare subpopulation of cells in the mammary gland (possibly a self-renewal stem or an early progenitor cell or both), OCT4 induces global epigenetic reprogramming in an epithelial target cell type of the breast. It is well documented that OCT4 is an essential reprogramming factor [42] and is sufficient to reprogram neural stem cells toward an induced pluripotent state [43]. In epithelial and other tissues, it is generally accepted that stem/progenitor cells reprogram at a higher frequency than more differentiated somatic cells, and this also suggests that the target cells mediating the OCT4 phenotype are not fully differentiated. However, to study whether OCT4 induces genome-wide epigenetic remodeling, global changes in DNA and histone methylation need to be evaluated in OTBCs.

To confirm the epithelial origin of OTBCs, we evaluated their differentiation potential by placing the OTBCs in differentiation conditions and performing a detection of specific CKs, which are a hallmark of epithelial cells. In 3D culture conditions, OTBCs formed TDLUs, which were morphologically very similar to those reported for breast stem and cancer stem cells [24]. When OTBCs were placed in 2D cultures, small populations of cells stained positive for myoepithelial markers (CK14, SMA, and Maspin) or luminal CKs (CK19) or both. These experiments demonstrated that OTBCs had an epithelial origin, and the cell target of OCT4 was possibly a primitive stem/progenitor cell. In self-renewal conditions, OTBCs exhibited antigenic signatures characteristic of prospective stem cells of the breast, such as low levels of CD133, high CD49f, and an absence of EpCAM expression. Given the current understanding of prospective signatures in the mammary gland hierarchy [8], these antigenic signatures are consistent with a putative breast stem/early progenitor cell identity. The finding that all OTBCs analyzed were EpCAM^- ^suggested that OTBCs do not originate from prospective luminal-restricted progenitor cells, which are presumably EpCAM^+^. However, it is also possible that OTBCs originate from myoepithelial CD10^+ ^restricted progenitors. In addition to being enriched in prospective stem cell signatures, OTBCs were enriched in the tumorigenic, cancer stem cell CD44^+^/CD24^- ^signature. Consistently, we found that as few as 50 cells derived from our OTBC lines was sufficient to generate tumors with metastatic colonization abilities in nude mice.

Histopathological analysis of the tumors in nude mice confirmed the epithelial origin of OTBCs. All OTBCs analyzed generated poorly differentiated carcinomas of the breast and revealed an epithelial morphology with a relatively high nuclear-to-cytoplasmic ratio and brisk mitotic activity. These tumors were negative for ER, PR, and HER2 and were positive for both OCT4 and the mesenchymal marker VIM. Examination of CK staining also revealed that a subset of tumor cells was immunoreactive for CK/pan-keratin, which further supports their classification as a poorly differentiated carcinoma.

To gain a mechanistic understanding of how OCT4 immortalized and transformed the target cells, we performed gene expression microarray experiments. The comparison of genome-wide transcriptional profiles of OTBCs with their parental lines revealed a gene signature that was over-represented in the newly discovered claudin-low intrinsic subtype of breast cancer. Claudin-low carcinomas were recently identified by Herschkowitz and colleagues [44] and further characterized by using a large database of human breast tumors [6,44] and cell lines [6]. Although claudin-low tumors are relatively rare (representing up to 11% of all breast cancers), they are associated with poor patient survival [6,10]. Claudin-low carcinomas uniquely express low levels of tight and adherent junction genes, including claudins and E-cadherin [6,12]. Hallmarks of these tumors include enrichment in EMT markers (VIM and Twist) and putative TIC markers (CD44^+^/CD24^- ^ratios) [10].

Recent genome-wide analysis suggests that this newly discovered intrinsic subtype of breast cancer is closely related to putative EpCAM^- ^mammary stem cells [8,9]. Basal-like breast cancer, which is associated with mutations in the tumor suppressor gene *BRCA1*, appears to be more closely related to an EpCAM^+ ^luminal-restricted progenitor cell population [8]. Further support for the hypothesis that claudin-low carcinomas may arise from primitive stem/progenitor cells is provided by clinical data, which show that TICs are enriched in patients with breast cancer after neo-adjuvant therapy [45]. Recent gene expression microarray analyses of these TICs revealed enrichment in EMT gene signatures [11]. Similarly, OTBCs exhibited enrichment in mesenchymal markers and TIC features. Compared with their parental lines, OTBCs upregulated the EMT TFs *SNAIL*, *TWIST*, and *ZEB1/2 *as well as microRNAs associated with EMT, such as miR-200s family members and miR-205. EMT has been associated with stemness. The forced expression of EMT TFs in immortalized breast epithelial cells led to stem cell-like characteristics and induction of TIC surface antigens [12].

Recently, ectopic expression of *OCT4 *and *NANOG *was shown to enhance malignancy and induce EMT in lung adenocarcinoma cell lines [46]. This finding confirms our results that link *OCT4 *and *NANOG *as potential oncogenes, which drive EMT processes in the mammary tissue. OCT4 expression was recently demonstrated in the MMTV-Wnt1 mouse models of breast cancer [47]. Recent work on epithelial ovarian cancer has shown that pluripotency TFs, such as OCT4 and NANOG, are overexpressed in poorly differentiated epithelial ovarian cancers. Furthermore, the RNAi knockdown of OCT4 in these cells prevented or blocked their ability to generate spheroids [16,48]. Likewise, a similar report in the MCF-7 breast cancer cell line demonstrated that the knockdown of OCT4 induced tumor cell death [17]. Our loss-of-function studies also outlined the crucial role of OCT4 and its downstream targets in maintaining self-renewal and EMT in our OTBC lines. We found that the hESC NOS target ZIC1 was upregulated in all OTBCs. Recent reports have suggested that ZIC1 is overexpressed in brain [49] and lung [50] tumors. Analysis of transcriptional profiles of large cohorts of human tumors revealed that *ZIC1 *mRNA is overexpressed in poorly differentiated carcinomas, including breast cancers [13]. We found that siRNA-mediated knockdown of ZIC1 suppressed the ability of OTBCs to form spheroids *in vitro*, outlining an important role of ZIC1 as a potential oncogene in claudin-low carcinomas. These data suggest that OTBCs can be used as model systems to identify oncogenic targets in claudin-low carcinomas.

In hESCs, OCT4 acts as a gatekeeper of self-renewal and master regulator of a TF network [33,51]. Indeed, knockdown of OCT4 in hESCs or epigenetic silencing of its promoter irreversibly blocks self-renewal and pluripotency and triggers differentiation gene programs. Consistent with the ability of OTBCs to maintain self-renewal, we found that these lines also activated the endogenous hESC TF network. We speculate that overexpression of OCT4 in a subpopulation of cells in the mammary gland was able to maintain these cells in a 'locked in' and undifferentiated state, limiting them from undergoing downstream lineage specification gene programs. This explanation is consistent with a mouse model of *OCT4 *cDNA overexpression, which demonstrates that OCT4 generates hyperplasia of the skin and colon by possibly targeting progenitor cells [15].

Although the exact mechanism by which OCT4 triggers the TIC-like phenotype needs further investigation, we speculate that gain of self-renewal ability is a complex genome-wide phenomenon that requires endogenous reactivation of a TIC self-renewal TF network. This model is consistent with our microarray data, which show that direct targets of NANOG, OCT4, and SOX2 (NOS targets), which are reasonably well characterized in hESCs, are also differentially regulated in OTBCs relative to their parental lines. Thus, OTBCs could mimic or even corrupt a basic hESC self-renewal TF network, which involves protein-protein associations acting in a combinatorial manner at specific promoter sites [50-54]. The characterization of this TIC-like TF network and specifically how this protein network differs from hESCs will require further study. In a TIC, this network may similarly involve associations between TFs, such as OCT4 and NANOG, and co-activator or co-repressor complexes as well as chromatin remodelers. This combinatorial occupancy of factors at specific promoters could result in the activation of potential oncogenes and self-renewal gene programs as well as the repression of selected tumor suppressor genes.

Importantly, our data suggest that NOS targets are regulated differently in TICs relative to hESCs. DKK1, an antagonist of the Wnt signaling pathway, is abundantly expressed in hESCs. In contrast, this target was found downregulated in OTBCs. Indeed, DKK1 is a secreted tumor suppressor in breast cancer [35,36]. Wnt signaling in breast cancer has been linked to EMT through stabilization of Snail [55] and upregulation of the EMT TFs SLUG and TWIST [56,57]. Overexpression of *DKK1 *in a breast cancer cell line resulted in an inhibition of self-renewal ability. Thus, downregulation of the NOS target *DKK1 *in OTBCs is consistent with gain of self-renewal ability and mesenchymal characteristics via the upregulation of EMT TFs.

In hESCs, OCT4 represses TFs involved in pattern specification, such as homeobox-containing proteins. In contrast, homeobox-containing TFs were highly enriched in our OTBC upregulated gene signature. The homeobox TF *SIX1 *was found upregulated in the OTBCs relative to parental lines. Overexpression of *SIX1 *in the mouse mammary gland promoted an expansion of stem/progenitor cells and subsequent tumor development [58]. In a parallel study, Micalizzi and colleagues [59] reported that overexpression of *SIX1 *also facilitated breast cancer metastasis by induction of EMT.

In conclusion, our data support a mechanism by which differential regulation of downstream targets of *OCT4 *led to activation of oncogenes and downregulation of tumor suppressors. Since OTBCs sustained aberrant self-renewal, it is possible that these cells gained TIC features by selective amplification of spheroids. A compromised tumor suppressor repertoire could result in subsequent selection of clones possessing tumorigenic ability. The molecular events leading to upregulation of TF genes and downregulation of multiple tumor suppressor genes are unknown at present; however, genetic and epigenetic events might be involved. In hESCs, activation of TF networks is associated with promoter de-methylation and gene reactivation. Loss of tumor suppressor functions in OTBCs could also involve genetic and epigenetic silencing mechanisms. Downstream epigenetic regulators of OCT4, such as DNMT3a/b, could be involved in this concerted silencing of tumor suppressor genes [60]. Alternatively, OCT4 and other self-renewal TFs could be associated with large silencing complexes involving HDACs, such as NODE [61] and NuRD [62], which have been well described in hESCs. Consistent with the idea of epigenetic modulation triggered by OCT4, we found that methyltransferase inhibitors and HDACis were able to partially reactivate tumor suppressor genes in OTBCs. In summary, our data describe the generation of novel claudin-low cell lines that could be used by breast cancer investigators to analyze genetic and epigenetic determinants of tumor initiation.

## Conclusions

In this article, we have shown that overexpression of *OCT4 *cDNA into normal primary breast epithelial preparations generated clonal populations of cells with aberrant self-renewal that developed tumor initiation ability. When injected in nude mice, these cells developed poorly differentiated, mesenchymal-enriched, and triple-negative breast carcinomas. *OCT4*-transduced breast colonies exhibited genome-wide signatures that are over-represented in the claudin-low intrinsic subtype of breast cancer. Our data suggest that OCT4 expands an early stem/progenitor cell and activates an embryonic-like TF network. Using siRNAs, we have validated the role of OCT4 and embryonic targets of OCT4, such as NANOG and ZIC1, in mediating the self-renewal phenotype. Our experimental approach provides a novel model system that can be used to identify therapeutic targets involved in breast cancer self-renewal and tumor initiation in a patient-specific manner.

## Abbreviations

3D: three-dimensional; 5-aza-2'dC: 5-aza-2'-deoxycytidine; CDH1: E-cadherin; CK: cytokeratin; CTG: Cell Titer Glo; DMEM: Dulbecco's modified Eagle's medium; EMT: epithelial-to-mesenchymal transition; EpCAM: epithelial cell adhesion molecule; ER: estrogen receptor; FBS: fetal bovine serum; HDACi: histone deacetylase inhibitor; hEGF: human epidermal growth factor; HER2: epidermal growth factor receptor 2; hESC: human embryonic stem cell; hTERT: human telomerase reverse transcriptase; IRB: institutional review board; MEBM: mammary epithelial basal media; MEF: mouse embryonic fibroblast; N-CAM: neural cell adhesion molecule; NOS: NANOG: OCT4 and SOX2; OTBC: OCT4-transduced breast cell; PBS: phosphate-buffered saline; PR: progesterone receptor; qRT-PCR: quantitative real-time polymerase chain reaction; RNAi: RNA interference; SCID: severe combined immunodeficient; siRNA: short interfering RNA; TDLU: terminal ductal lobular unit; TF: transcription factor; TIC: tumor-initiating cell; VIM: vimentin.

## Competing interests

The authors declare that they have no competing interests.

## Authors' contributions

ASB participated in generation of OTBCs; conducted fluorescence-activated cell sorting, immunofluorescence, and animal experiments and gene expression analyses; and helped to prepare the manuscript. AGR conducted tumor histology and gene expression analyses and helped to prepare the manuscript. BTR conducted siRNA knockdown and tumor histology. XY performed Western blots. HQ participated in generation of OTBCs. JPH conducted histopathological analysis of the tumors and CK8/18 immunohistochemistry of tumor sections. EZ and LMG conducted miRNA detection. PB participated in generation of OTBCs, helped to prepare the manuscript, and provided project direction. All authors read and approved the final manuscript.

## Supplementary Material

Additional file 1 **Table S1**. Primer sequences. Click here for file

Additional file 2 **Supplementary methods**. Click here for file

Additional file 3 **Table S2**. Antibodies used in this study. Click here for file

Additional file 4 **Figure S1. Flow cytometry analysis of EpCAM expression in the parental line p86 and the OTBCs86-L1 and L2 in self-renewal and differentiation conditions**. The forward Scatter (FCS) channel was plotted in the y-axis and EpCAM fluorescence was plotted in the x-axis. Experiments were repeated at least three times with similar results. Click here for file

Additional file 5 **Figure S2**. **Flow cytometry analysis of antigenic phenotypes characteristic of bipotent Mammary Stem Cells**. (CD133^low^CD49f^+^EpCAM^-^) and CSCs (CD44^+^CD24^-^) in the parental line p86 and the OTBC clones. The forward Scatter (FCS) channel was plotted in the y-axis and the fluorescence of the cell surface antigens (CD44 or CD24) was plotted in the x-axis. Experiments were repeated at least three times with similar results. Click here for file

Additional file 6 **Table S3**. Summary of animal experiments. Click here for file

Additional file 7 **Table S4**. Genes Up-regulated in OTBCs. Click here for file

Additional file 8 **Table S5**. Genes Down-regulated in OTBCs. Click here for file

Additional file 9 **Figure S3. *OCT4 *mRNA is found particularly enriched in the basal and claudin-low subtype of breast cancer**. *OCT4 *expression analysis in 336 breast tumors from the UNC database. Each column represents the relative transcript abundance (in log2 space) for each gene, and each row represents each molecular subtype. Mean expression of *OCT4 *across subtypes. Click here for file

Additional file 10 **Figure S4. NOS targets are over-represented across breast cancers**. NOS signature comprehensive analysis in 336 breast tumors from the UNC database. Each column represents the relative transcript abundance (in log2 space) for each gene, and each row represents each molecular subtype. Mean expression of the NOS signature across subtypes. Click here for file

Additional file 11 **Figure S5. Tumor suppressor genes in OTBCs are re-expressed upon treatment with DNA methyltransferase and histone deacetylase inhibitors**. OTBCs86-L1 and OTBCs48-1 lines were treated with 5 M of 5-aza-2'-deoxycytodine (5-Aza-2'dC), 0.5 M Suberoylanilide Hydroxamic Acid (SAHA), and 100 nM Trichostatin-A (TSA) for 48 hours and gene expression was assessed by RT-PCR. Bar graphs represent the mean SD of three independent experiments. Data was analyzed with student t test and p-values were set at p 0.05. **(a) **Expression levels of *Maspin *and *CDH1 *were normalized to vehicle control **(b) **Expression levels of *MGMT *and *DKK1 *normalized to vehicle control. Click here for file
