# Supplementary material for: Generation of tumor-initiating cells by exogenous delivery of OCT4 transcription factor
Source: Breast Cancer Res. 2011 Sep 27;13(5):R94. doi: 10.1186/bcr3019 (PMC3262206; doi:10.1186/bcr3019)
Supplement: Additional file 1 — Table S1. Primer sequences. [file bcr3019-S1.DOCX]

**Table S1. Primer sequences**

| **Genes** | **Probe or**  **Applied Biosystems Ref. #** | **Forward primer** | **Reverse primer** |
| --- | --- | --- | --- |
| **GAPDH** | *FAM/^#^MGB #4333764F | - | - |
| **GAPDH** | ^&^SYBR | CCATGTTCGTCATGGGTGTGA | CATGGACTGTGGTCATGAGT |
| **OCT4 (*POU5F1*)** | Hs03005111_g1 | - | - |
| **SOX2** | Hs01053049_s1 | - | - |
| **P16 (*CDKN2A*)** | Hs99999189_m1 | - | - |
| **NANOG** | Hs02387400_g1 | - | - |
| **Maspin (*SERPIN B5*)** | *FAM-CAACAAGACAGACACCAA ACCAGTGCAG-MGB | CGACCAGACCAAAATCCTTG | GAACGTGGACTCCATGGTC |
| **E-Cadherin (*CDH1*)** | ^@^TAM-TGCCACATACACTCTC-MGB | AGGTGACAGAGCCTCTGGATAGA | TGGATGACACAGCGTGAGAGA |
| **TWIST** | Hs00361186_m1 | - | - |
| **SNAIL1** | Hs00195591_m1 | - | - |
| **SNAIL2** | Hs00950344_m1 | - | - |
| **ZEB1** | SYBR | AAGAAAGTGTTACAGATGCAGCTG | CCCTGGTAACACTGTCTGGTC |
| **ZEB2** | SYBR | AGGCATATGGTGACGCACAA | CTTGAACTTGCGGTTACCTGC |
| **hTERT (human telomerase reverse transcriptase gene)** | SYBR | CATGGGCACGTCCGCAA | GGCGTGGTGGCACATGAA |
| **P21^WAF1^** | SYBR | TGGAGACTCTCAGGGTCGAAA | GGCGTTTGGAGTAGAAATC |
| **MGMT** | SYBR | CAGCCCGAGGCTATCGAA | AACGACTCTTGCTGGAAAACG |
| **DKK1** | SYBR | TTGAGTCCTTCTGAGATG | TTGATAGCGTTGGAATTG |
| **ZIC1** | SYBR | CCGCAAGAAGCACATGCA | CGTGTAGGACTTGTCGCACATC |
| **miR-200a** | 000502 | - | - |
| **miR-200b** | 002251 | - | - |
| **miR-200c** | 002300 | - | - |
| **miR-141** | 000463 | - | - |
| **miR-205** | 002218 | - | - |
| **NR-U6** | 002752 | - | - |

*FAM (6-carboxy-fluorescein)

^@^TAM (TAMRA, dihydrocyclopyrroloindole tripeptide)

#MGB (Minor Groove Binder)

&SYBR (N',N'-dimethyl-N-[4-[(E)-(3-methyl-1,3-benzothiazol-2-ylidene)methyl]-1-phenylquinolin-1-ium-2-yl]-N-propylpropane-1,3-diamine)
