# Supplementary material for: Generation of tumor-initiating cells by exogenous delivery of OCT4 transcription factor
Source: Breast Cancer Res. 2011 Sep 27;13(5):R94. doi: 10.1186/bcr3019 (PMC3262206; doi:10.1186/bcr3019)
Supplement: Additional file 2 — Supplementary methods. [file bcr3019-S2.DOCX]

**Supplementary methods**

**Immunofluorescence**

Cells were seeded in fibronectin-coated 8-well chambers (LabTEK, Thermo Scientific, Rochester, NY) for 24 hours, fixed with 4% formaldehyde, and permeabilized with 0.5% Triton-X100 in phosphate buffered saline (PBS) and blocked with 5% bovine serum albumin (BSA) for 2 hours. Next, the cells were incubated with primary antibodies at 4°C overnight and with secondary antibodies for one hour at room temperature. Primary and secondary antibodies were used according to the manufacturer’s recommendations and are listed in Table S2.

**Flow Cytometry**

Cells were labeled with an un-conjugated anti-CD133 (Abcam, Cambridge, MA, USA) and fluorophore conjugated primary antibodies anti-CD24 (phycoerythrin (PE)), anti-EpCAM (PE), anti-CD44 (fluorescein isothiocyanate (FITC)), and anti-CD49f (Cyanine 5 (Cy5)). Incubation was performed for 60 min on ice in PBS with 2% FBS, followed by two washes in PBS with 2% FBS. Cells labeled with CD133 were detected by a PE-conjugated goat anti-rabbit secondary antibody. All the conjugated antibodies were purchased from BD, Becton Dickinson, Palo Alto, CA, USA. Data was acquired with a FACScalibur (Becton Dickinson, San Jose, CA, USA) flow cytometer. Flow data was analyzed using BD CellQuest™(Becton Dickinson, San Jose, CA, USA) .

**Western blotting**

Cells were lysed with 1x RIPA buffer (Invitrogen, Carlsbad, CA, USA) in the presence of a protease inhibitor cocktail (Roche, Indianapolis, IN, USA). 25 g of total extract was loaded per well of a 10% polyacrylamide gel and run under SDS/PAGE denaturing conditions. Proteins were transferred to a nitrocellulose membrane and blocked for 1 hour in 5% milk. Primary and secondary antibodies from respective companies were applied at the concentrations listed in Table S2. Membranes were then developed (ECL-Plus, GE Healthcare, Piscataway, NJ, USA) and visualized by autoradiography.

**Immunohistochemistry**

Tissue sections were fixed in 10% formalin for 24 hours and paraffin embedded. Slides were cut in 5 m serial sections by the UNC Histology Core Facility. Sections were de-paraffinized with xylene and rehydrated with ethanol and water. Each tumor sample was stained with hematoxylin and eosin (H&E). Sections were blocked in 5% BSA for 1 hour. Immunohistochemistry was performed with primary antibodies shown in Table S2. The primary antibodies were detected with secondary biotin-conjugated antibodies (Table S2) and streptavidin-conjugated with horseradish peroxidase (HRP). All primary antibodies were incubated overnight at 4C and secondary antibodies were applied for 2 hours the following day at room temperature. Streptavidin-HRP was applied for 30 minutes. The reaction was visualized with Diaminobenzidine (DAB), and all sections were counterstained with hematoxylin (Sigma, St. Louis, MO, USA).

**Small molecule epigenetic inhibitors**

Trichostatin-A (TSA, Sigma, St. Louis, MO, USA) and 5-aza-2’deoxycytodine (5-Aza-2’dC, Sigma, St. Louis, MO, USA) were dissolved in DMSO. Suberoylanilide Hydroxamic Acid (SAHA, BioVision, Mountain View, Ca, USA) was dissolved in ethanol. Drugs were diluted at final concentrations in growth medium, and cells were exposed to the drugs for 48 hours and processed by real-time quantification.
