# Supplementary material for: Generation of tumor-initiating cells by exogenous delivery of OCT4 transcription factor
Source: Breast Cancer Res. 2011 Sep 27;13(5):R94. doi: 10.1186/bcr3019 (PMC3262206; doi:10.1186/bcr3019)
Supplement: Additional file 3 — Table S2. Antibodies used in this study. [file bcr3019-S3.DOC]

**Table S2. Antibodies used in this study**

| **Antibody** | **Company** | **Dilution and application** |
| --- | --- | --- |
| Nestin | BD (#611659) | WB 1:500, |
| CD10 | Abcam (#ab951) | WB 1:1000 |
| OCT-4 | Abcam (#ab18976) | WB 1:500, IH 1:250 |
| E-Cadherin | BD (#610181) | WB 1:2500 |
| Maspin | BD (#554292) | WB 1:500 |
| Vimentin | BD (#550513) | WB 1:5000, 1:100 |
| N-CAM | Santa Cruz (#sc106) | WB 1:500 |
| ER | Abcam (#ab16660-250) | WB 1:200, IH 1:100 |
| PR | Abcam (#ab32085) | WB 1:1000, IH 1:200 |
| ZIC1 | Novus Biologicals (#NBP1-02874) | WB 1:1,000 |
| TUBULIN | Sigma (#T5168) | WB 1:10,000 |
| P16 | Abcam (#ab50282) | WB 1:1,000 |
| Cytokeratin 8/18 | Leica (PA0067) | IH 1:100 |
| Pankeratin Ae1/Ae3 | Abcam (ab27988) | IH 1:40 |
| Mouse IgG (Biotin) | Amersham (rpn1001v1) | IH 1:200 |
| Rabbit IgG (Biotin) | Abcam (ab6720-1) | IH 1:200 |
| Streptavidin HRP | Amersham (rpn1231v) | IH 1:300 |
| Rabbit IgG | Jackson ImmunoResearch (#111-035-144) | WB 1:10000 |
| Mouse IgG | Cell Signaling (#7076) | WB 1:5000 |
| Goat Anti-Mouse (Alexa 488) | Invitrogen (A11001) | IF 1:1000 |
| Goat Anti-Mouse (Alexa 594) | Invitrogen (A11032) | IF 1:1000 |
| Goat Anti-rabbit (Alexa 555) | Invitrogen (A21428) | IF 1:1000 |
| CD24-PE | BD (# 555426) | FC 1:100 |
| CD44-FITC | BD (# 555478) | FC 1:100 |
| CD49f-PE-CY5 | BD (# 551129) | FC 1:100 |
| EpCAM-PE | BD (# 347199) | FC 1:100 |
| CD133 | Abcam (#ab19898) | FC 1:100 |
| Cytokeratin 14 | Abcam (ab49747) | IF 1:250 |
| Cytokeratin 19 | Abcam (ab15463) | IF 1:250 |

- WB, Western Blot
- IH, Immunohistochemistry
- IF, Immunofluorescence
- FC, Flow Cytometry
