# Supplementary figures and images for: Generation of tumor-initiating cells by exogenous delivery of OCT4 transcription factor
Source: Breast Cancer Res. 2011 Sep 27;13(5):R94. doi: 10.1186/bcr3019 (PMC3262206; doi:10.1186/bcr3019)

Figure S1

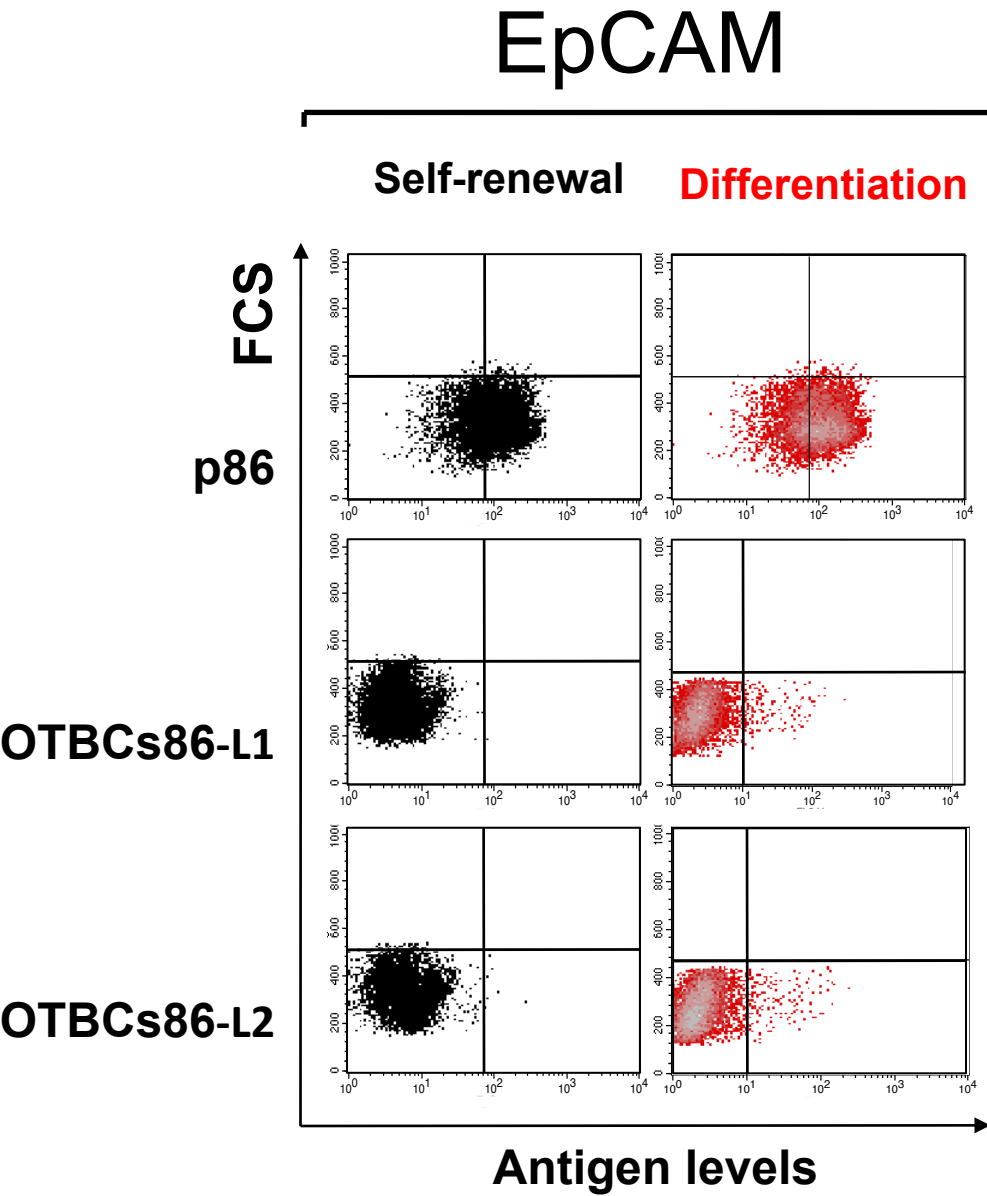

Supplement: Additional file 4 — Figure S1. Flow cytometry analysis of EpCAM expression in the parental line p86 and the OTBCs86-L1 and L2 in self-renewal and differentiation conditions. The forward Scatter (FCS) channel was plotted in the y-axis and EpCAM fluorescence was plotted in the x-axis. Experiments were repeated at least three times with similar results. [file bcr3019-S4.PDF]

Figure S2

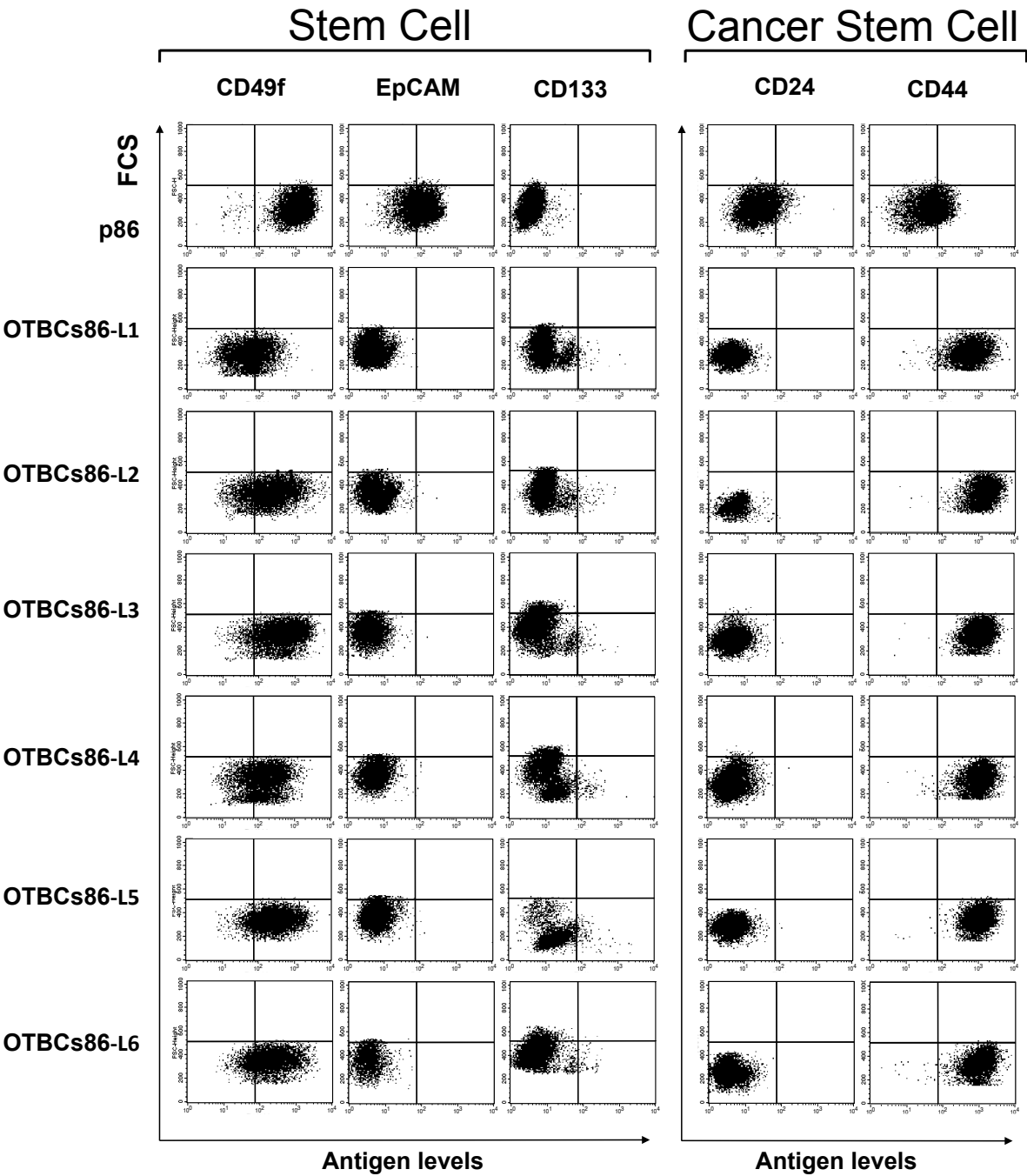

Supplement: Additional file 5 — Figure S2. Flow cytometry analysis of antigenic phenotypes characteristic of bipotent Mammary Stem Cells. (CD133lowCD49f+EpCAM-) and CSCs (CD44+CD24-) in the parental line p86 and the OTBC clones. The forward Scatter (FCS) channel was plotted in the y-axis and the fluorescence of the cell surface antigens (CD44 or CD24) was plotted in the x-axis. Experiments were repeated at least three times with similar results. [file bcr3019-S5.PDF]

Figure S3

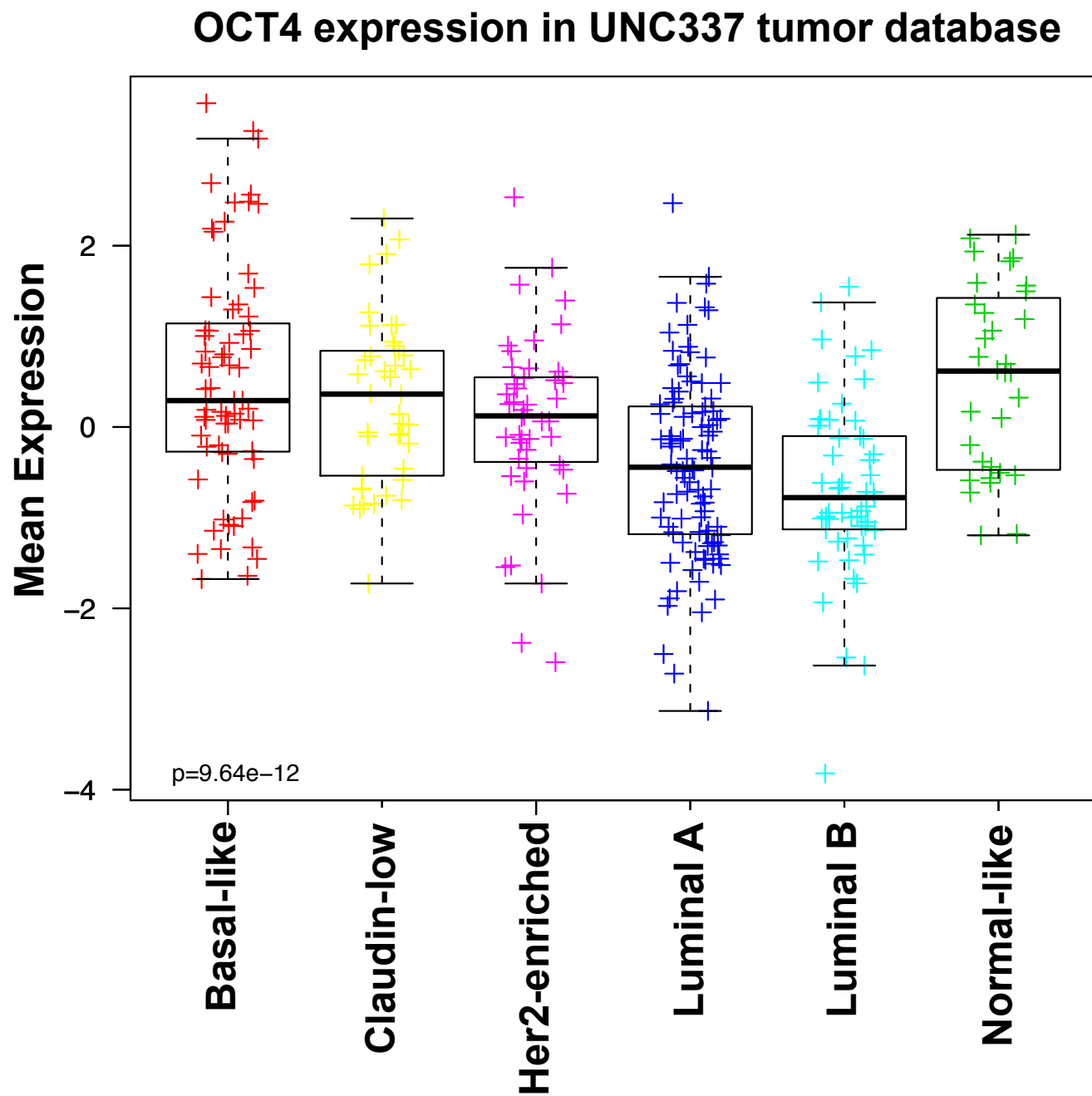

Supplement: Additional file 9 — Figure S3. OCT4 mRNA is found particularly enriched in the basal and claudin-low subtype of breast cancer. OCT4 expression analysis in 336 breast tumors from the UNC database. Each column represents the relative transcript abundance (in log2 space) for each gene, and each row represents each molecular subtype. Mean expression of OCT4 across subtypes. [file bcr3019-S9.PDF]

Figure S4

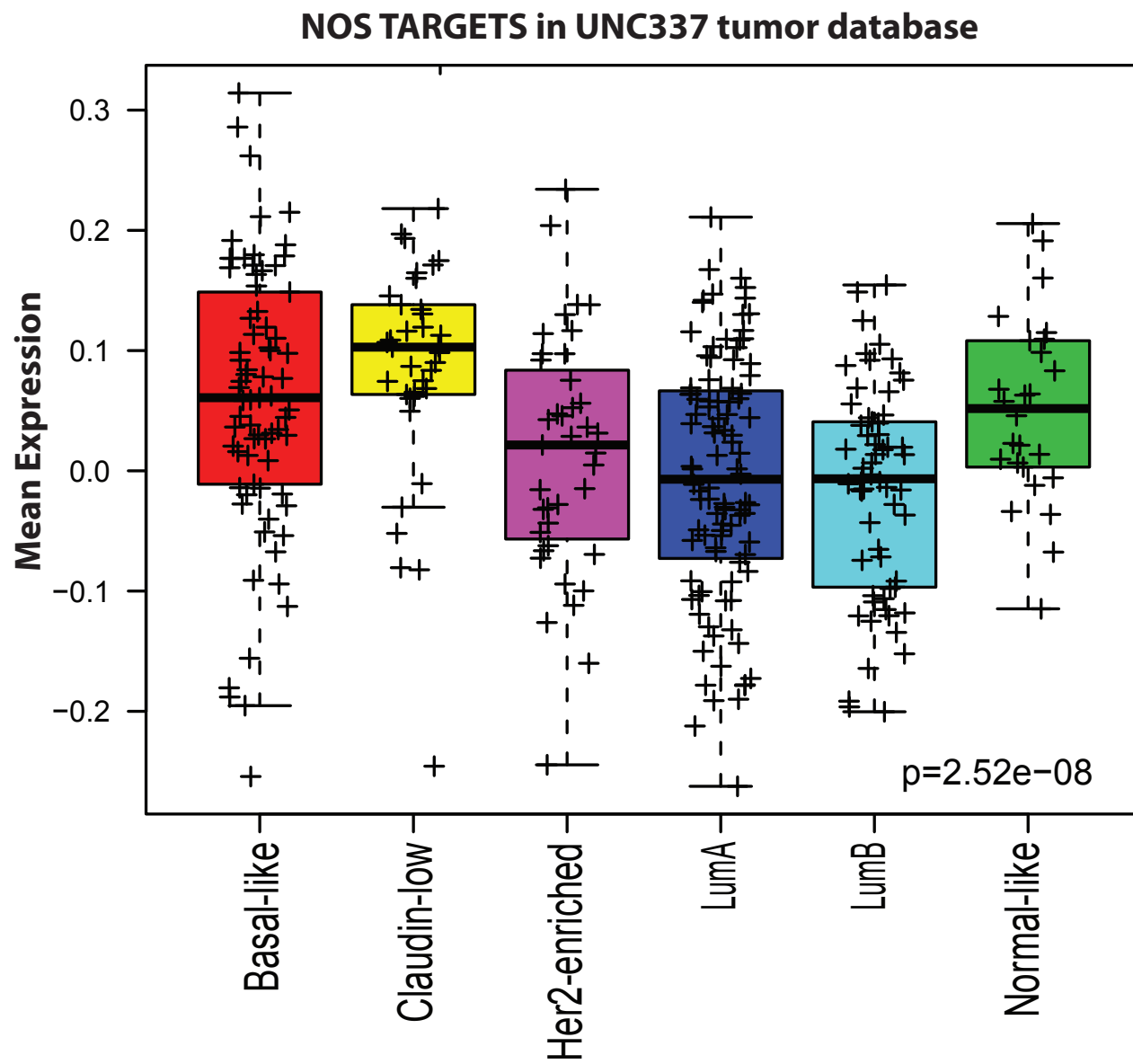

Supplement: Additional file 10 — Figure S4. NOS targets are over-represented across breast cancers. NOS signature comprehensive analysis in 336 breast tumors from the UNC database. Each column represents the relative transcript abundance (in log2 space) for each gene, and each row represents each molecular subtype. Mean expression of the NOS signature across subtypes. [file bcr3019-S10.PDF]

Figure S5

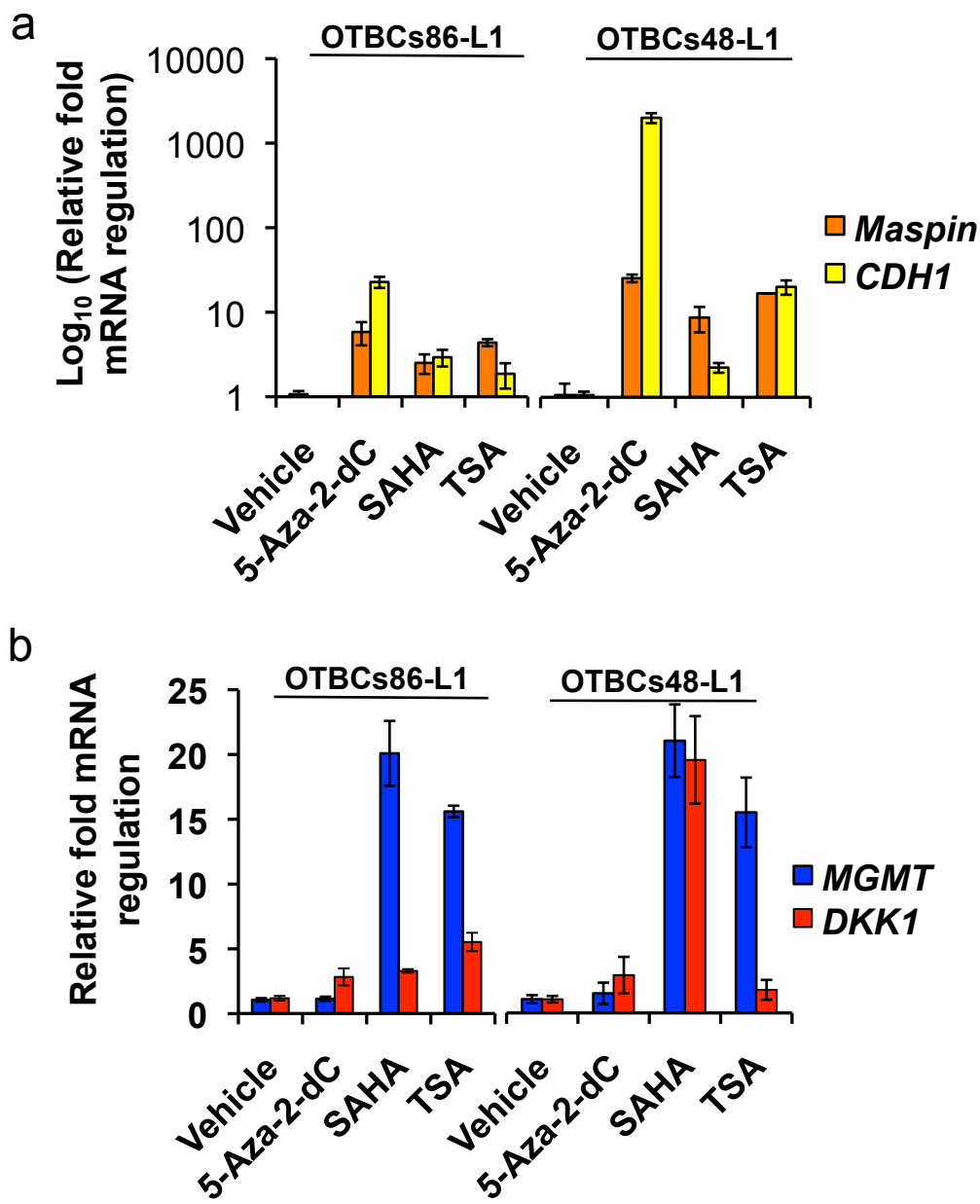

Supplement: Additional file 11 — Figure S5. Tumor suppressor genes in OTBCs are re-expressed upon treatment with DNA methyltransferase and histone deacetylase inhibitors. OTBCs86-L1 and OTBCs48-1 lines were treated with 5 M of 5-aza-2'-deoxycytodine (5-Aza-2'dC), 0.5 M Suberoylanilide Hydroxamic Acid (SAHA), and 100 nM Trichostatin-A (TSA) for 48 hours and gene expression was assessed by RT-PCR. Bar graphs represent the mean SD of three independent experiments. Data was analyzed with student t test and p-values were set at p 0.05. (a) Expression levels of Maspin and CDH1 were normalized to vehicle control (b) Expression levels of MGMT and DKK1 normalized to vehicle control. [file bcr3019-S11.PDF]
