# Supplementary material for: Generation of tumor-initiating cells by exogenous delivery of OCT4 transcription factor
Source: Breast Cancer Res. 2011 Sep 27;13(5):R94. doi: 10.1186/bcr3019 (PMC3262206; doi:10.1186/bcr3019)
Supplement: Additional file 6 — Table S3. Summary of animal experiments. [file bcr3019-S6.DOCX]

**Table S3. Summary of animal experiments**

| **Experiment** | **Cell Line** | **Number of cells injected** | **Number of Tumors** | **Days** |
| --- | --- | --- | --- | --- |
| Fat pad | OTBCs86-L1 | 1x10^5^ | 8/8 | 16 |
|  | OTBCs86-L2 | 1x10^5^ | 2/2 | 22 |
|  | OTBCs86-L4 | 1x10^5^ | 2/2 | 20 |
|  | OTBCs86-L5 | 1x10^5^ | 2/2 | 22 |
|  | OTBCs86-L6 | 1x10^5^ | 2/2 | 21 |
|  | | | | |
| Subcutaneous | OTBCs48-L1 | 1x10^6^ | 4/4 | 28 |
|  | p86 | 3x10^6^ | 0/2 | N/A |
|  | p86 | 1x10^6^ | 0/2 | N/A |
|  | OTBCs86-L1 | 1x10^6^ | 4/2 | 9 |
|  | OTBCs86-L2 | 1x10^6^ | 2/2 | 20 |
|  | OTBCs86-L5 | 1x10^6^ | 2/2 | 20 |
|  | | | | |
| Intracardiac | OTBCs86-L1 | 1x10^5^ | 3/5 | 56 |
|  | | | | |
| Subcutaneous  Dilutions | OTBCs86-L1 | 1 000 000 | 8/8 | 9 |
|  |  | 1x10^5^ | 6/6 | 16 |
|  |  | 1000 | 8/8 | 25 |
|  |  | 50 | 5/8 | 45 |
|  |  | 1 | 0 | >90 |

N/A, not applicable; No tumor growth was observed
