# Supplementary material for: Generation of tumor-initiating cells by exogenous delivery of OCT4 transcription factor
Source: Breast Cancer Res. 2011 Sep 27;13(5):R94. doi: 10.1186/bcr3019 (PMC3262206; doi:10.1186/bcr3019)
Supplement: Additional file 7 — Table S4. Genes Up-regulated in OTBCs. [file bcr3019-S7.DOCX]

**Table S4. Genes Up-regulated in OCT4-transduced breast cells (OTBCs)**

| **GeneID** | **Gene Symbol** | **Name** |
| --- | --- | --- |
| 55816 | DOK5 | Docking protein 5 |
| 7021 | TFAP2B | Transcription factor AP-2 beta (activating enhancer binding protein 2 beta) |
| 10561 | IFI44 | Interferon-induced protein 44 |
| 84969 | C20orf100 | Chromosome 20 open reading frame 100 |
| 55714 | ODZ3 | Odz, odd Oz/ten-m homolog 3 (Drosophila) |
| 642 | BLMH | Bleomycin hydrolase |
| 51529 | ANAPC11 | APC11 anaphase promoting complex subunit 11 homolog (yeast) |
| 25791 | NGEF | Neuronal guanine nucleotide exchange factor |
| 9465 | AKAP7 | A kinase (PRKA) anchor protein 7 |
| 9353 | SLIT2 | Slit homolog 2 (Drosophila) |
| 115399 | DKFZp761L1518 | Hypothetical protein DKFZp761L1518 |
| 54510 | PCDH18 | Protocadherin 18 |
| 9201 | DCAMKL1 | Doublecortin and CaM kinase-like 1 |
| 24138 | IFIT5 | Interferon-induced protein with tetratricopeptide repeats 5 |
| 9194 | SLC16A7 | Solute carrier family 16 (monocarboxylic acid transporters), member 7 |
| 64397 | SH3BP3 | SH3-domain binding protein 3 |
| 56994 | CHPT1 | Choline phosphotransferase 1 |
| 11007 | DIPA | Hepatitis delta antigen-interacting protein A |
| 3590 | IL11RA | Interleukin 11 receptor, alpha |
| 51079 | GRIM19 | Cell death-regulatory protein GRIM19 |
| 2593 | GAMT | Guanidinoacetate N-methyltransferase |
| 55068 | FLJ10094 | Hypothetical protein FLJ10094 |
| 23236 | PLCB1 | Phospholipase C, beta 1 (phosphoinositide-specific) |
| 3708 | ITPR1 | Inositol 1,4,5-triphosphate receptor, type 1 |
| 55790 | ChGn | Chondroitin beta1,4 N-acetylgalactosaminyltransferase |
| 10092 | ARPC5 | Actin related protein 2/3 complex, subunit 5, 16kDa |
| 51161 | LOC51161 | G20 protein |
| 22921 | MSRB | Methionine sulfoxide reductase B |
| 4131 | MAP1B | Microtubule-associated protein 1B |
| 79792 | GSDMDC1 | Gasdermin domain containing 1 |
| 25927 | C2orf32 | Chromosome 2 open reading frame 32 |
| 22852 | ANKRD26 | Ankyrin repeat domain 26 |
| 9653 | HS2ST1 | Heparan sulfate 2-O-sulfotransferase 1 |
| 66005 | MGC3234 | Hypothetical protein MGC3234 |
| 8270 | DXS9879E | DNA segment on chromosome X (unique) 9879 expressed sequence |
| 4329 | ALDH6A1 | Aldehyde dehydrogenase 6 family, member A1 |
| 9435 | CHST2 | Carbohydrate (N-acetylglucosamine-6-O) sulfotransferase 2 |
| 9023 | CH25H | Cholesterol 25-hydroxylase |
| 22998 | KIAA1102 | KIAA1102 protein |
| 1445 | CSK | C-src tyrosine kinase |
| 57180 | ARP3BETA | Actin-related protein 3-beta |
| 11319 | HSGT1 | Suppressor of S. cerevisiae gcr2 |
| 22841 | RAB11FIP2 | RAB11 family interacting protein 2 (class I) |
| 10194 | SDCCAG33 | Serologically defined colon cancer antigen 33 |
| 55509 | SNFT | Jun dimerization protein p21SNFT |
| 57689 | NGL-1 | Netrin-G1 ligand |
| 11041 | B3GNT6 | UDP-GlcNAc:betaGal beta-1,3-N-acetylglucosaminyltransferase 6 |
| 57134 | MAN1C1 | Mannosidase, alpha, class 1C, member 1 |
| 124790 | FLJ32384 | Hypothetical protein MGC39389 |
| 5321 | PLA2G4A | Phospholipase A2, group IVA (cytosolic, calcium-dependent) |
| 51372 | HSPC016 | Hypothetical protein HSPC016 |
| 1355 | COX15 | COX15 homolog, cytochrome c oxidase assembly protein (yeast) |
| 55728 | N4BP2 | Nedd4 binding protein 2 |
| 139818 | DOCK11 | Dedicator of cytokinesis 11 |
| 55437 | ALS2CR2 | Amyotrophic lateral sclerosis 2 (juvenile) chromosome region, candidate 2 |
| 89927 | C16orf45 | Chromosome 16 open reading frame 45 |
| 401588 | LOC401588 | Hypothetical LOC401588 |
| 255104 | LOC255104 | Hypothetical protein LOC255104 |
| 2530 | FUT8 | Fucosyltransferase 8 (alpha (1,6) fucosyltransferase) |
| 54681 | PH-4 | Hypoxia-inducible factor prolyl 4-hydroxylase |
| 6495 | SIX1 | Sine oculis homeobox homolog 1 (Drosophila) |
| 374395 | LOC374395 | Similar to RIKEN cDNA 1810059G22 |
| 54985 | HCFC1R1 | Host cell factor C1 regulator 1 (XPO1 dependant) |
| 57670 | KIAA1549 | KIAA1549 protein |
| 4485 | MST1 | Macrophage stimulating 1 (hepatocyte growth factor-like) |
| 128272 | ARHGEF19 | Rho guanine nucleotide exchange factor (GEF) 19 |
| 4208 | MEF2C | MADS box transcription enhancer factor 2, polypeptide C (myocyte enhancer factor 2C) |
| 2027 | ENO3 | Enolase 3 (beta, muscle) |
| 81854 | MGC3771 | Hypothetical protein MGC3771 |
| 79640 | FLJ23584 | Hypothetical protein FLJ23584 |
| 1745 | DLX1 | Distal-less homeo box 1 |
| 8209 | C21orf33 | Chromosome 21 open reading frame 33 |
| 84450 | ZNF512 | Zinc finger protein 512 |
| 54916 | C14orf101 | Chromosome 14 open reading frame 101 |
| 56731 | SLC2A4RG | SLC2A4 regulator |
| 4212 | MEIS2 | Meis1, myeloid ecotropic viral integration site 1 homolog 2 (mouse) |
| 7423 | VEGFB | Vascular endothelial growth factor B |
| 285368 | FLJ33674 | Hypothetical protein FLJ33674 |
| 23272 | RAP140 | Retinoblastoma-associated protein 140 |
| 10058 | ABCB6 | ATP-binding cassette, sub-family B (MDR/TAP), member 6 |
| 23129 | PLXND1 | Plexin D1 |
| 57215 | THAP11 | THAP domain containing 11 |
| 201229 | LOC201229 | Hypothetical protein LOC201229 |
| 5634 | PRPS2 | Phosphoribosyl pyrophosphate synthetase 2 |
| 79187 | FSD1 | Fibronectin type 3 and SPRY domain containing 1 |
| 123904 | UNQ2446 | MRCC2446 |
| 875 | CBS | Cystathionine-beta-synthase |
| 8733 | GPAA1 | GPAA1P anchor attachment protein 1 homolog (yeast) |
| 55116 | FLJ10315 | Hypothetical protein FLJ10315 |
| 85013 | MGC13159 | Hypothetical protein MGC13159 |
| 84446 | KIAA1811 | KIAA1811 protein |
| 54828 | BCAS3 | Breast carcinoma amplified sequence 3 |
| 2146 | EZH2 | Enhancer of zeste homolog 2 (Drosophila) |
| 1891 | ECH1 | Enoyl Coenzyme A hydratase 1, peroxisomal |
| 84153 | AYP1 | AYP1 protein |
| 55663 | FLJ20626 | Hypothetical protein FLJ20626 |
| 56848 | SPHK2 | Sphingosine kinase 2 |
| 9890 | LPPR4 | Plasticity related gene 1 |
| 23331 | KIAA1043 | KIAA1043 protein |
| 22836 | RHOBTB3 | Rho-related BTB domain containing 3 |
| 8542 | APOL1 | Apolipoprotein L, 1 |
| 23321 | TRIM2 | Tripartite motif-containing 2 |
| 23314 | SATB2 | SATB family member 2 |
| 4008 | LMO7 | LIM domain 7 |
| 55529 | DKFZp762O076 | Hypothetical protein DKFZp762O076 |
| 23179 | RGL1 | Ral guanine nucleotide dissociation stimulator-like 1 |
| 8905 | AP1S2 | Adaptor-related protein complex 1, sigma 2 subunit |
| 54495 | FLJ20793 | FLJ20793 protein |
| 3778 | KCNMA1 | Potassium large conductance calcium-activated channel, subfamily M, alpha member 1 |
| 6474 | SHOX2 | Short stature homeobox 2 |
| 8968 | HIST1H3F | Histone 1, H3f |
| 2995 | GYPC | Glycophorin C (Gerbich blood group) |
| 5063 | PAK3 | P21 (CDKN1A)-activated kinase 3 |
| 51374 | C2orf28 | Chromosome 2 open reading frame 28 |
| 79960 | PHF17 | PHD finger protein 17 |
| 5264 | PHYH | Phytanoyl-CoA hydroxylase (Refsum disease) |
| 57198 | ATP8B2 | ATPase, Class I, type 8B, member 2 |
| 3516 | RBPSUH | Recombining binding protein suppressor of hairless (Drosophila) |
| 2068 | ERCC2 | Excision repair cross-complementing rodent repair deficiency, complementation group 2 (xeroderma pigmentosum D) |
| 9694 | KIAA0103 | KIAA0103 |
| 23174 | ZCCHC14 | Zinc finger, CCHC domain containing 14 |
| 284900 | KIAA1648 | KIAA1648 protein |
| 4005 | LMO2 | LIM domain only 2 (rhombotin-like 1) |
| 8405 | SPOP | Speckle-type POZ protein |
| 2781 | GNAZ | Guanine nucleotide binding protein (G protein), alpha z polypeptide |
| 54856 | FLJ20203 | Hypothetical protein FLJ20203 |
| 7090 | TLE3 | Transducin-like enhancer of split 3 (E(sp1) homolog, Drosophila) |
| 84148 | MYST1 | MYST histone acetyltransferase 1 |
| 92070 | MGC21675 | Hypothetical protein MGC21675 |
| 5521 | PPP2R2B | Protein phosphatase 2 (formerly 2A), regulatory subunit B (PR 52), beta isoform |
| 149076 | FLJ25476 | FLJ25476 protein |
| 3248 | HPGD | Hydroxyprostaglandin dehydrogenase 15-(NAD) |
| 8809 | IL18R1 | Interleukin 18 receptor 1 |
| 66008 | ALS2CR3 | Amyotrophic lateral sclerosis 2 (juvenile) chromosome region, candidate 3 |
| 57763 | ANKRA2 | Ankyrin repeat, family A (RFXANK-like), 2 |
| 3106 | HLA-B | Major histocompatibility complex, class I, B |
| 27010 | TPK1 | Thiamin pyrophosphokinase 1 |
| 27072 | VPS41 | Vacuolar protein sorting 41 (yeast) |
| 9931 | HELZ | Hypothetical protein LOC284019 |
| 283316 | M160 | Scavenger receptor cysteine-rich type 1 protein M160 |
| 10026 | PIGK | Phosphatidylinositol glycan, class K |
| 2953 | GSTT2 | Glutathione S-transferase theta 2 |
| 79090 | MGC2650 | Hypothetical protein MGC2650 |
| 50854 | C6orf48 | Chromosome 6 open reading frame 48 |
| 2941 | GSTA4 | Glutathione S-transferase A4 |
| 6604 | SMARCD3 | SWI/SNF related, matrix associated, actin dependent regulator of chromatin, subfamily d, member 3 |
| 10735 | STAG2 | Stromal antigen 2 |
| 5754 | PTK7 | PTK7 protein tyrosine kinase 7 |
| 8402 | SLC25A11 | Solute carrier family 25 (mitochondrial carrier; oxoglutarate carrier), member 11 |
| 8499 | PPFIA2 | Protein tyrosine phosphatase, receptor type, f polypeptide (PTPRF), interacting protein (liprin), alpha 2 |
| 84883 | AMID | Apoptosis-inducing factor (AIF)-like mitochondrion-associated inducer of death |
| 9352 | TXNL1 | Thioredoxin-like 1 |
| 11168 | PSIP1 | PC4 and SFRS1 interacting protein 1 |
| 79712 | GTDC1 | Glycosyltransferase-like 1 |
| 57706 | KIAA1608 | KIAA1608 |
| 1290 | COL5A2 | Collagen, type V, alpha 2 |
| 83690 | LOC83690 | CocoaCrisp |
| 51057 | LOC51057 | Hypothetical protein LOC51057 |
| 85002 | MGC16279 | Hypothetical protein MGC16279 |
| 1112 | CHES1 | Checkpoint suppressor 1 |
| 9145 | SYNGR1 | Synaptogyrin 1 |
| 158234 | RG9MTD3 | RNA (guanine-9-) methyltransferase domain containing 3 |
| 7716 | ZNF161 | Zinc finger protein 161 |
| 9839 | ZFHX1B | Zinc finger homeobox 1b |
| 10039 | PARP3 | Poly (ADP-ribose) polymerase family, member 3 |
| 23420 | NOMO1 | NODAL modulator 3 |
| 10965 | ZAP128 | Peroxisomal long-chain acyl-coA thioesterase |
| 286336 | C9orf59 | Chromosome 9 open reading frame 59 |
| 23258 | RAB6IP1 | RAB6 interacting protein 1 |
| 5871 | MAP4K2 | Mitogen-activated protein kinase kinase kinase kinase 2 |
| 10457 | GPNMB | Glycoprotein (transmembrane) nmb |
| 51015 | CGI-111 | CGI-111 protein |
| 34 | ACADM | Acyl-Coenzyme A dehydrogenase, C-4 to C-12 straight chain |
| 9049 | AIP | Aryl hydrocarbon receptor interacting protein |
| 23127 | GLT25D2 | Glycosyltransferase 25 domain containing 2 |
| 8996 | NOL3 | Nucleolar protein 3 (apoptosis repressor with CARD domain) |
| 25956 | SEC31L2 | SEC31-like 2 (S. cerevisiae) |
| 10979 | PLEKHC1 | Pleckstrin homology domain containing, family C (with FERM domain) member 1 |
| 56255 | DJ971N18.2 | Hypothetical protein DJ971N18.2 |
| 3613 | IMPA2 | Inositol(myo)-1(or 4)-monophosphatase 2 |
| 9148 | NEURL | Neuralized-like (Drosophila) |
| 257054 | MGC25181 | Hypothetical protein MGC25181 |
| 4901 | NRL | Neural retina leucine zipper |
| 5495 | PPM1B | Protein phosphatase 1B (formerly 2C), magnesium-dependent, beta isoform |
| 113189 | D4ST1 | Dermatan 4 sulfotransferase 1 |
| 54940 | OCIAD1 | OCIA domain containing 1 |
| 84327 | ZBED3 | Zinc finger, BED domain containing 3 |
| 10234 | LRRC17 | Leucine rich repeat containing 17 |
| 26873 | OPLAH | 5-oxoprolinase (ATP-hydrolysing) |
| 6721 | SREBF2 | Sterol regulatory element binding transcription factor 2 |
| 153561 | LOC153561 | Hypothetical LOC389295 |
| 402 | ARL2 | ADP-ribosylation factor-like 2 |
| 29072 | HYPB | Huntingtin interacting protein B |
| 26580 | BSCL2 | Bernardinelli-Seip congenital lipodystrophy 2 (seipin) |
| 35 | ACADS | Acyl-Coenzyme A dehydrogenase, C-2 to C-3 short chain |
| 9308 | CD83 | CD83 antigen (activated B lymphocytes, immunoglobulin superfamily) |
| 2584 | GALK1 | Galactokinase 1 |
| 2911 | GRM1 | Glutamate receptor, metabotropic 1 |
| 7181 | NR2C1 | Nuclear receptor subfamily 2, group C, member 1 |
| 29969 | HIC | I-mfa domain-containing protein |
| 6091 | ROBO1 | Roundabout, axon guidance receptor, homolog 1 (Drosophila) |
| 197319 | FLJ12986 | Hypothetical protein FLJ12986 |
| 170463 | SSBP4 | Single stranded DNA binding protein 4 |
| 143282 | C10orf13 | Chromosome 10 open reading frame 13 |
| 4671 | BIRC1 | Baculoviral IAP repeat-containing 1 |
| 90198 | SBP1 | HBV pre-s2 binding protein 1 |
| 6727 | SRP14 | Signal recognition particle 14kDa (homologous Alu RNA binding protein) |
| 51701 | NLK | Nemo like kinase |
| 55683 | FLJ10081 | Hypothetical protein FLJ10081 |
| 55012 | C14orf10 | Chromosome 14 open reading frame 10 |
| 22826 | DNAJC8 | DnaJ (Hsp40) homolog, subfamily C, member 8 |
| 160622 | GRASP | GRP1 (general receptor for phosphoinositides 1)-associated scaffold protein |
| 10211 | FLOT1 | Flotillin 1 |
| 53918 | PELO | Integrin, alpha 1 |
| 117178 | SSX2IP | Synovial sarcoma, X breakpoint 2 interacting protein |
| 92369 | SSB4 | SPRY domain-containing SOCS box protein SSB-4 |
| 64130 | LIN7B | Lin-7 homolog B (C. elegans) |
| 54530 | FLJ20054 | Family with sequence similarity 31, member B |
| 56952 | PRTFDC1 | Phosphoribosyl transferase domain containing 1 |
| 83938 | C10orf11 | Chromosome 10 open reading frame 11 |
| 280636 | C11orf31 | Chromosome 11 open reading frame 31 |
| 84265 | MGC3200 | Hypothetical protein LOC284615 |
| 53615 | MBD3 | Methyl-CpG binding domain protein 3 |
| 23155 | MCLC | Mid-1-related chloride channel 1 |
| 9666 | DZIP3 | Zinc finger DAZ interacting protein 3 |
| 522 | ATP5J | ATP synthase, H+ transporting, mitochondrial F0 complex, subunit F6 |
| 5981 | RFC1 | Replication factor C (activator 1) 1, 145kDa |
| 3418 | IDH2 | Isocitrate dehydrogenase 2 (NADP+), mitochondrial |
| 25900 | HOM-TES-103 | HOM-TES-103 tumor antigen-like |
| 56160 | NDNL2 | Necdin-like 2 |
| 54899 | PXK | PX domain containing serine/threonine kinase |
| 22934 | RPIA | Ribose 5-phosphate isomerase A (ribose 5-phosphate epimerase) |
| 23328 | SASH1 | SAM and SH3 domain containing 1 |
| 4594 | MUT | Methylmalonyl Coenzyme A mutase |
| 126272 | FLJ38944 | Hypothetical protein FLJ38944 |
| 27244 | SESN1 | Sestrin 1 |
| 3107 | HLA-C | Major histocompatibility complex, class I, C |
| 51690 | LSM7 | LSM7 homolog, U6 small nuclear RNA associated (S. cerevisiae) |
| 79001 | VKORC1 | Vitamin K epoxide reductase complex, subunit 1 |
| 83707 | MGC11134 | TRNA splicing 2' phosphotransferase 1 |
| 25894 | DKFZP434I216 | DKFZP434I216 protein |
| 988 | CDC5L | CDC5 cell division cycle 5-like (S. pombe) |
| 5828 | PXMP3 | Peroxisomal membrane protein 3, 35kDa (Zellweger syndrome) |
| 80727 | TTYH3 | Tweety homolog 3 (Drosophila) |
| 1954 | EGFL4 | EGF-like-domain, multiple 4 |
| 54556 | ING3 | Inhibitor of growth family, member 3 |
| 10953 | TOMM34 | Translocase of outer mitochondrial membrane 34 |
| 79668 | PARP8 | Poly (ADP-ribose) polymerase family, member 8 |
| 79140 | MGC1203 | Hypothetical protein MGC1203 |
| 128977 | LOC128977 | Hypothetical protein LOC128977 |
| 7475 | WNT6 | Wingless-type MMTV integration site family, member 6 |
| 55609 | SUHW3 | Suppressor of hairy wing homolog 3 (Drosophila) |
| 3183 | HNRPC | Heterogeneous nuclear ribonucleoprotein C (C1/C2) |
| 9338 | TCEAL1 | Transcription elongation factor A (SII)-like 1 |
| 23126 | POGZ | Pogo transposable element with ZNF domain |
| 4832 | NME3 | Non-metastatic cells 3, protein expressed in |
| 259217 | HSPA12A | Heat shock 70kDa protein 12A |
| 25942 | SIN3A | SIN3 homolog A, transcriptional regulator (yeast) |
| 51293 | 8D6A | 8D6 antigen |
| 1047 | CLGN | Calmegin |
| 4154 | MBNL1 | Muscleblind-like (Drosophila) |
| 4130 | MAP1A | Microtubule-associated protein 1A |
| 142679 | DUSP19 | Dual specificity phosphatase 19 |
| 6443 | SGCB | Sarcoglycan, beta (43kDa dystrophin-associated glycoprotein) |
| 55089 | SLC38A4 | Solute carrier family 38, member 4 |
| 3105 | HLA-A | Major histocompatibility complex, class I, A |
| 25805 | BAMBI | BMP and activin membrane-bound inhibitor homolog (Xenopus laevis) |
| 1052 | CEBPD | CCAAT/enhancer binding protein (C/EBP), delta |
| 576 | BAI2 | Brain-specific angiogenesis inhibitor 2 |
| 7155 | TOP2B | Topoisomerase (DNA) II beta 180kDa |
| 3135 | HLA-G | HLA-G histocompatibility antigen, class I, G |
| 11163 | NUDT4 | Nudix (nucleoside diphosphate linked moiety X)-type motif 4 |
| 57664 | PLEKHA4 | Pleckstrin homology domain containing, family A (phosphoinositide binding specific) member 4 |
| 84541 | TA-KRP | T-cell activation kelch repeat protein |
| 5554 | PRH1 | Proline-rich protein HaeIII subfamily 1 |
| 55768 | NGLY1 | N-glycanase 1 |
| 23193 | GANAB | Glucosidase, alpha; neutral AB |
| 23168 | KIAA0252 | KIAA0252 |
| 29105 | GTL3 | Likely ortholog of mouse gene trap locus 3 |
| 1857 | DVL3 | Dishevelled, dsh homolog 3 (Drosophila) |
| 1892 | ECHS1 | Enoyl Coenzyme A hydratase, short chain, 1, mitochondrial |
| 154791 | HSPC268 | Hypothetical protein HSPC268 |
| 127396 | MGC27466 | Hypothetical protein MGC27466 |
| 112476 | LOC112476 | Similar to lymphocyte antigen 6 complex, locus G5B; G5b protein; open reading frame 31 |
| 6596 | SMARCA3 | SWI/SNF related, matrix associated, actin dependent regulator of chromatin, subfamily a, member 3 |
| 98 | ACYP2 | Acylphosphatase 2, muscle type |
| 114134 | SLC2A13 | Solute carrier family 2 (facilitated glucose transporter), member 13 |
| 3759 | KCNJ2 | Potassium inwardly-rectifying channel, subfamily J, member 2 |
| 6794 | STK11 | Serine/threonine kinase 11 (Peutz-Jeghers syndrome) |
| 9015 | TAF1A | TATA box binding protein (TBP)-associated factor, RNA polymerase I, A, 48kDa |
| 10536 | LEPREL2 | Leprecan-like 2 |
| 783 | CACNB2 | Calcium channel, voltage-dependent, beta 2 subunit |
| 26577 | PCOLCE2 | Procollagen C-endopeptidase enhancer 2 |
| 1544 | CYP1A2 | Cytochrome P450, family 1, subfamily A, polypeptide 2 |
| 3992 | FADS1 | Fatty acid desaturase 1 |
| 6878 | TAF6 | TAF6 RNA polymerase II, TATA box binding protein (TBP)-associated factor, 80kDa |
| 84166 | NOD27 | Nucleotide-binding oligomerization domains 27 |
| 83538 | DKFZP434H0115 | Hypothetical protein DKFZp434H0115 |
| 10618 | TGOLN2 | Trans-golgi network protein 2 |
| 53342 | IL17D | Interleukin 17D |
| 116540 | MRPL53 | Mitochondrial ribosomal protein L53 |
| 84809 | MGC12760 | Hypothetical protein MGC12760 |
| 8547 | FCN3 | Ficolin (collagen/fibrinogen domain containing) 3 (Hakata antigen) |
| 970 | TNFSF7 | Tumor necrosis factor (ligand) superfamily, member 7 |
| 57562 | KIAA1377 | KIAA1377 protein |
| 2812 | GP1BB | Glycoprotein Ib (platelet), beta polypeptide |
| 10073 | RNUT1 | RNA, U transporter 1 |
| 3760 | KCNJ3 | Potassium inwardly-rectifying channel, subfamily J, member 3 |
| 4281 | MID1 | Midline 1 (Opitz/BBB syndrome) |
| 23345 | SYNE1 | Spectrin repeat containing, nuclear envelope 1 |
| 401549 | C9orf110 | Chromosome 9 open reading frame 110 |
| 145553 | MGC5987 | Hypothetical protein MGC5987 |
| 149175 | FLJ31434 | Hypothetical protein FLJ31434 |
| 55565 | LOC55565 | Hypothetical protein LOC55565 |
| 55070 | DET1 | De-etiolated 1 |
| 5193 | PEX12 | Peroxisomal biogenesis factor 12 |
| 5383 | PMS2L5 | Postmeiotic segregation increased 2-like 1 |
| 57106 | KLP1 | K562 cell-derived leucine-zipper-like protein 1 |
| 113246 | GRCC10 | Likely ortholog of mouse gene rich cluster, C10 gene |
| 54880 | BCOR | BCL6 co-repressor |
| 79624 | C6orf211 | Chromosome 6 open reading frame 211 |
| 3437 | IFIT3 | Interferon-induced protein with tetratricopeptide repeats 3 |
| 5297 | PIK4CA | Phosphatidylinositol 4-kinase, catalytic, alpha polypeptide |
| 23780 | APOL2 | Apolipoprotein L, 2 |
| 84839 | MGC15631 | Hypothetical protein MGC15631 |
| 6919 | TCEA2 | Transcription elongation factor A (SII), 2 |
| 10016 | PDCD6 | Programmed cell death 6 |
| 123811 | FLJ31153 | Hypothetical protein FLJ31153 |
| 9590 | AKAP12 | A kinase (PRKA) anchor protein (gravin) 12 |
| 1738 | DLD | Dihydrolipoamide dehydrogenase (E3 component of pyruvate dehydrogenase complex, 2-oxo-glutarate complex, branched chain keto acid dehydrogenase complex) |
| 4289 | MKLN1 | Muskelin 1, intracellular mediator containing kelch motifs |
| 10522 | DEAF1 | Deformed epidermal autoregulatory factor 1 (Drosophila) |
| 253635 | FLJ38348 | Hypothetical protein FLJ38348 |
| 10811 | NOXA1 | NADPH oxidase activator 1 |
| 65250 | FLJ13231 | Hypothetical protein FLJ13231 |
| 64776 | C11orf1 | Chromosome 11 open reading frame 1 |
| 26137 | ZBTB20 | Zinc finger and BTB domain containing 20 |
| 8225 | GTPBP6 | GTP binding protein 6 (putative) |
| 4157 | MC1R | Melanocortin 1 receptor (alpha melanocyte stimulating hormone receptor) |
| 23398 | KIAA0073 | KIAA0073 protein |
| 55850 | MDS032 | Uncharacterized hematopoietic stem/progenitor cells protein MDS032 |
| 7703 | RNF110 | Ring finger protein 110 |
| 4548 | MTR | 5-methyltetrahydrofolate-homocysteine methyltransferase |
| 4354 | MPP1 | Membrane protein, palmitoylated 1, 55kDa |
| 3915 | LAMC1 | Laminin, gamma 1 (formerly LAMB2) |
| 79632 | C6orf60 | Chromosome 6 open reading frame 60 |
| 7175 | TPR | Translocated promoter region (to activated MET oncogene) |
| 11228 | C12orf2 | Chromosome 12 open reading frame 2 |
| 80755 | MGC2744 | Hypothetical protein MGC2744 |
| 376940 | ZC3HDC6 | Zinc finger CCCH type domain containing 6 |
| 94097 | SFXN5 | Sideroflexin 5 |
| 8975 | USP13 | Ubiquitin specific protease 13 (isopeptidase T-3) |
| 84140 | FLJ13305 | Hypothetical protein FLJ13305 |
| 25937 | WWTR1 | WW domain containing transcription regulator 1 |
| 7158 | TP53BP1 | Tumor protein p53 binding protein, 1 |
| 55251 | C20orf36 | Chromosome 20 open reading frame 36 |
| 23208 | SYT11 | Synaptotagmin XI |
| 1998 | ELF2 | E74-like factor 2 (ets domain transcription factor) |
| 55066 | PDPR | Pyruvate dehydrogenase phosphatase regulatory subunit |
| 1070 | CETN3 | Centrin, EF-hand protein, 3 (CDC31 homolog, yeast) |
| 51454 | GULP1 | GULP, engulfment adaptor PTB domain containing 1 |
| 266812 | NAP1L5 | Nucleosome assembly protein 1-like 5 |
| 161779 | PGBD4 | PiggyBac transposable element derived 4 |
| 51523 | CXXC5 | CXXC finger 5 |
| 92312 | LOC92312 | Hypothetical protein LOC92312 |
| 23157 | 40792 | Septin 6 |
| 23059 | CLUAP1 | Clusterin associated protein 1 |
| 80833 | APOL3 | Apolipoprotein L, 3 |
| 22982 | KIAA0934 | KIAA0934 |
| 7918 | BAT4 | HLA-B associated transcript 4 |
| 2791 | GNG11 | Guanine nucleotide binding protein (G protein), gamma 11 |
| 23464 | GCAT | Glycine C-acetyltransferase (2-amino-3-ketobutyrate coenzyme A ligase) |
| 6510 | SLC1A5 | Solute carrier family 1 (neutral amino acid transporter), member 5 |
| 58487 | ZF | HCF-binding transcription factor Zhangfei |
| 4905 | NSF | N-ethylmaleimide-sensitive factor |
| 54555 | DDX49 | DEAD (Asp-Glu-Ala-Asp) box polypeptide 49 |
| 3131 | HLF | Hepatic leukemia factor |
| 84992 | MGC14156 | Hypothetical protein MGC14156 |
| 27090 | SIAT7D | Sialyltransferase 7D ((alpha-N-acetylneuraminyl-2,3-beta-galactosyl-1,3)-N-acetyl galactosaminide alpha-2,6-sialyltransferase) |
| 8019 | BRD3 | Bromodomain containing 3 |
| 8500 | PPFIA1 | Protein tyrosine phosphatase, receptor type, f polypeptide (PTPRF), interacting protein (liprin), alpha 1 |
| 161823 | 0 | Similar to CG11994-PA |
| 7915 | ALDH5A1 | Aldehyde dehydrogenase 5 family, member A1 (succinate-semialdehyde dehydrogenase) |
| 9604 | RNF14 | Ring finger protein 14 |
| 84275 | MGC4399 | Mitochondrial carrier protein |
| 78999 | LRFN4 | Leucine rich repeat and fibronectin type III domain containing 4 |
| 210 | ALAD | Aminolevulinate, delta-, dehydratase |
| 162972 | ZNF550 | Zinc finger protein 550 |
| 9684 | LRRC14 | Leucine rich repeat containing 14 |
| 2735 | GLI | Glioma-associated oncogene homolog (zinc finger protein) |
| 51097 | CGI-49 | CGI-49 protein |
| 9403 | 40801 | 15 kDa selenoprotein |
| 22937 | SCAP | SREBP CLEAVAGE-ACTIVATING PROTEIN |
| 23390 | ZDHHC17 | Zinc finger, DHHC domain containing 17 |
| 7089 | TLE2 | Transducin-like enhancer of split 2 (E(sp1) homolog, Drosophila) |
| 1016 | CDH18 | Cadherin 18, type 2 |
| 8322 | FZD4 | Frizzled homolog 4 (Drosophila) |
| 5980 | REV3L | REV3-like, catalytic subunit of DNA polymerase zeta (yeast) |
| 4856 | NOV | Nephroblastoma overexpressed gene |
| 203197 | C9orf91 | Chromosome 9 open reading frame 91 |
| 80017 | C14orf159 | Chromosome 14 open reading frame 159 |
| 147906 | MGC15476 | Thymus expressed gene 3-like |
| 60526 | FLJ21820 | Hypothetical protein FLJ21820 |
| 171023 | ASXL1 | Additional sex combs like 1 (Drosophila) |
| 29890 | RBM15B | RNA binding motif protein 15B |
| 5198 | PFAS | Phosphoribosylformylglycinamidine synthase (FGAR amidotransferase) |
| 81578 | COL21A1 | Collagen, type XXI, alpha 1 |
| 285908 | LOC285908 | Hypothetical protein LOC285908 |
| 23412 | COMMD3 | COMM domain containing 3 |
| 8193 | DPF1 | D4, zinc and double PHD fingers family 1 |
| 56257 | FLJ20257 | Hypothetical protein FLJ20257 |
| 2686 | GGTL3 | Gamma-glutamyltransferase-like 3 |
| 83700 | JAM3 | Junctional adhesion molecule 3 |
| 8577 | TMEFF1 | Transmembrane protein with EGF-like and two follistatin-like domains 1 |
| 8706 | B3GALT3 | UDP-Gal:betaGlcNAc beta 1,3-galactosyltransferase, polypeptide 3 |
| 91694 | FLJ23749 | Hypothetical protein FLJ23749 |
| 23047 | APRIN | Androgen-induced proliferation inhibitor |
| 29952 | DPP7 | Dipeptidylpeptidase 7 |
| 51661 | FKBP7 | FK506 binding protein 7 |
| 2178 | FANCE | Fanconi anemia, complementation group E |
| 5937 | RBMS1 | RNA binding motif, single stranded interacting protein 1 |
| 1153 | CIRBP | Cold inducible RNA binding protein |
| 92521 | HCMOGT-1 | Sperm antigen HCMOGT-1 |
| 65259 | C16orf28 | Chromosome 16 open reading frame 28 |
| 6645 | SNTB2 | Syntrophin, beta 2 (dystrophin-associated protein A1, 59kDa, basic component 2) |
| 2639 | GCDH | Glutaryl-Coenzyme A dehydrogenase |
| 83871 | RAB34 | RAB34, member RAS oncogene family |
| 11336 | SEC6L1 | SEC6-like 1 (S. cerevisiae) |
| 85026 | C9orf37 | Chromosome 9 open reading frame 37 |
| 10198 | MPHOSPH9 | M-phase phosphoprotein 9 |
| 7049 | TGFBR3 | Transforming growth factor, beta receptor III (betaglycan, 300kDa) |
| 57561 | ARRDC3 | Arrestin domain containing 3 |
| 221409 | SPATS1 | Spermatogenesis associated, serine-rich 1 |
| 92483 | LDHAL6B | Lactate dehydrogenase A-like 6B |
| 596 | BCL2 | B-cell CLL/lymphoma 2 |
| 54552 | FLJ10613 | Hypothetical protein FLJ10613 |
| 84678 | FBXL10 | F-box and leucine-rich repeat protein 10 |
| 83872 | FIBL-6 | Hemicentin |
| 2512 | FTL | Ferritin, light polypeptide |
| 8559 | PRPF18 | PRP18 pre-mRNA processing factor 18 homolog (yeast) |
| 8694 | DGAT1 | Diacylglycerol O-acyltransferase homolog 1 (mouse) |
| 58525 | WIZ | Widely-interspaced zinc finger motifs |
| 147495 | APCDD1 | Adenomatosis polyposis coli down-regulated 1 |
| 10923 | PC4 | Activated RNA polymerase II transcription cofactor 4 |
| 55260 | FLJ10922 | Hypothetical protein FLJ10922 |
| 5826 | ABCD4 | ATP-binding cassette, sub-family D (ALD), member 4 |
| 80325 | ABTB1 | Ankyrin repeat and BTB (POZ) domain containing 1 |
| 54386 | TERF2IP | Telomeric repeat binding factor 2, interacting protein |
| 23245 | ASTN2 | Astrotactin 2 |
| 55750 | FLJ10842 | Hypothetical protein FLJ10842 |
| 5928 | RBBP4 | Retinoblastoma binding protein 4 |
| 84836 | MGC15429 | Hypothetical protein MGC15429 |
| 8991 | SELENBP1 | Selenium binding protein 1 |
| 5886 | RAD23A | RAD23 homolog A (S. cerevisiae) |
| 116028 | MGC24665 | Hypothetical protein MGC24665 |
| 1741 | DLG3 | Discs, large homolog 3 (neuroendocrine-dlg, Drosophila) |
| 10120 | ACTR1B | ARP1 actin-related protein 1 homolog B, centractin beta (yeast) |
| 26122 | EPC2 | Enhancer of polycomb homolog 2 (Drosophila) |
| 57157 | PHTF2 | Putative homeodomain transcription factor 2 |
| 3430 | IFI35 | Interferon-induced protein 35 |
| 1028 | CDKN1C | Cyclin-dependent kinase inhibitor 1C (p57, Kip2) |
| 10924 | SMPDL3A | Sphingomyelin phosphodiesterase, acid-like 3A |
| 9031 | BAZ1B | Bromodomain adjacent to zinc finger domain, 1B |
| 5316 | PKNOX1 | PBX/knotted 1 homeobox 1 |
| 92 | ACVR2 | Activin A receptor, type II |
| 22927 | HABP4 | Hyaluronan binding protein 4 |
| 51321 | LOC51321 | Hypothetical protein LOC51321 |
| 7957 | EPM2A | Epilepsy, progressive myoclonus type 2A, Lafora disease (laforin) |
| 143279 | HECTD2 | HECT domain containing 2 |
| 22980 | KIAA1049 | KIAA1049 protein |
| 5824 | PEX19 | Peroxisomal biogenesis factor 19 |
| 57593 | KIAA1442 | KIAA1442 protein |
| 57161 | PELI2 | Pellino homolog 2 (Drosophila) |
| 10302 | SNAPC5 | Small nuclear RNA activating complex, polypeptide 5, 19kDa |
| 9779 | TBC1D5 | TBC1 domain family, member 5 |
| 27347 | STK39 | Serine threonine kinase 39 (STE20/SPS1 homolog, yeast) |
| 4776 | NFATC4 | Nuclear factor of activated T-cells, cytoplasmic, calcineurin-dependent 4 |
| 9789 | KIAA0102 | KIAA0102 gene product |
| 10025 | THRAP5 | Thyroid hormone receptor associated protein 5 |
| 284129 | SLC26A11 | Solute carrier family 26, member 11 |
| 6320 | SCGF | Stem cell growth factor; lymphocyte secreted C-type lectin |
| 51616 | TAF9L | TAF9-like RNA polymerase II, TATA box binding protein (TBP)-associated factor, 31kDa |
| 10365 | KLF2 | Kruppel-like factor 2 (lung) |
| 6094 | ROM1 | Retinal outer segment membrane protein 1 |
| 29780 | PARVB | Parvin, beta |
| 171024 | SYNPO2 | Synaptopodin 2 |
| 3910 | LAMA4 | Laminin, alpha 4 |
| 28227 | PR48 | Protein phosphatase 2A 48 kDa regulatory subunit |
| 137209 | ZNF572 | Zinc finger protein 572 |
| 4439 | MSH5 | MutS homolog 5 (E. coli) |
| 2879 | GPX4 | Glutathione peroxidase 4 (phospholipid hydroperoxidase) |
| 6821 | SUOX | Sulfite oxidase |
| 3670 | ISL1 | ISL1 transcription factor, LIM/homeodomain, (islet-1) |
| 83986 | C16orf9 | Chromosome 16 open reading frame 9 |
| 6549 | SLC9A2 | Solute carrier family 9 (sodium/hydrogen exchanger), isoform 2 |
| 5141 | PDE4A | Phosphodiesterase 4A, cAMP-specific (phosphodiesterase E2 dunce homolog, Drosophila) |
| 6553 | SLC9A5 | Solute carrier family 9 (sodium/hydrogen exchanger), isoform 5 |
| 1027 | CDKN1B | Cyclin-dependent kinase inhibitor 1B (p27, Kip1) |
| 10130 | TXNDC7 | Thioredoxin domain containing 7 (protein disulfide isomerase) |
| 8321 | FZD1 | Frizzled homolog 1 (Drosophila) |
| 9663 | LPIN2 | Lipin 2 |
| 219771 | C10orf9 | Chromosome 10 open reading frame 9 |
| 463 | ATBF1 | AT-binding transcription factor 1 |
| 715 | C1R | Complement component 1, r subcomponent |
| 84928 | FLJ14803 | Hypothetical protein FLJ14803 |
| 965 | CD58 | CD58 antigen, (lymphocyte function-associated antigen 3) |
| 3054 | HCFC1 | Host cell factor C1 (VP16-accessory protein) |
| 2078 | ERG | V-ets erythroblastosis virus E26 oncogene like (avian) |
| 202018 | FLJ90013 | Hypothetical protein FLJ90013 |
| 23475 | QPRT | Quinolinate phosphoribosyltransferase (nicotinate-nucleotide pyrophosphorylase (carboxylating)) |
| 10449 | ACAA2 | Acetyl-Coenzyme A acyltransferase 2 (mitochondrial 3-oxoacyl-Coenzyme A thiolase) |
| 7629 | ZNF76 | Zinc finger protein 76 (expressed in testis) |
| 8913 | CACNA1G | Calcium channel, voltage-dependent, alpha 1G subunit |
| 56922 | MCCC1 | Methylcrotonoyl-Coenzyme A carboxylase 1 (alpha) |
| 6882 | TAF11 | TAF11 RNA polymerase II, TATA box binding protein (TBP)-associated factor, 28kDa |
| 89781 | HPS4 | Hermansky-Pudlak syndrome 4 |
| 84264 | HAGHL | Hydroxyacylglutathione hydrolase-like |
| 7157 | TP53 | Tumor protein p53 (Li-Fraumeni syndrome) |
| 54463 | FLJ20152 | Hypothetical protein FLJ20152 |
| 10157 | AASS | Aminoadipate-semialdehyde synthase |
| 5078 | PAX4 | Paired box gene 4 |
| 133383 | MGC33648 | Hypothetical protein MGC33648 |
| 4205 | MEF2A | MADS box transcription enhancer factor 2, polypeptide A (myocyte enhancer factor 2A) |
| 94107 | MGC14327 | Hypothetical protein MGC14327 |
| 54664 | FLJ11273 | Hypothetical protein FLJ11273 |
| 81618 | ITM2C | Integral membrane protein 2C |
| 6622 | SNCA | Synuclein, alpha (non A4 component of amyloid precursor) |
| 89894 | LOC89894 | Hypothetical protein BC000282 |
| 258010 | DKFZp313A2432 | Hypothetical protein DKFZp313A2432 |
| 11274 | USP18 | Ubiquitin specific protease 18 |
| 54537 | FAM35A | Family with sequence similarity 35, member A |
| 9736 | USP34 | Ubiquitin specific protease 34 |
| 144165 | PRICKLE1 | Prickle-like 1 (Drosophila) |
| 389782 | 0 | Hypothetical LOC389782 |
