# Supplementary material for: Generation of tumor-initiating cells by exogenous delivery of OCT4 transcription factor
Source: Breast Cancer Res. 2011 Sep 27;13(5):R94. doi: 10.1186/bcr3019 (PMC3262206; doi:10.1186/bcr3019)
Supplement: Additional file 8 — Table S5. Genes Down-regulated in OTBCs. [file bcr3019-S8.DOCX]

**Table S5. Genes down-regulated in OCT4-transduced breast cells (OTBCs)**

| **GeneID** | **Gene Symbol** | **Name** |
| --- | --- | --- |
| 1308 | COL17A1 | Collagen, type XVII, alpha 1 |
| 3855 | KRT7 | Keratin 7 |
| 374 | AREG | Amphiregulin (schwannoma-derived growth factor) |
| 3861 | KRT14 | Keratin 14 (epidermolysis bullosa simplex, Dowling-Meara, Koebner) |
| 2810 | SFN | Stratifin |
| 3853 | KRT6A | Keratin 6A |
| 6273 | S100A2 | S100 calcium binding protein A2 |
| 3868 | KRT16 | Keratin 16 (focal non-epidermolytic palmoplantar keratoderma) |
| 10653 | SPINT2 | Serine protease inhibitor, Kunitz type, 2 |
| 5349 | FXYD3 | FXYD domain containing ion transport regulator 3 |
| 810 | CALML3 | Calmodulin-like 3 |
| 57402 | S100A14 | S100 calcium binding protein A14 |
| 79098 | SARG | Specifically androgen-regulated protein |
| 286887 | KRT6E | Keratin 6E |
| 4071 | TM4SF1 | Transmembrane 4 superfamily member 1 |
| 23650 | TRIM29 | Tripartite motif-containing 29 |
| 148641 | SLC35F3 | Solute carrier family 35, member F3 |
| 3918 | LAMC2 | Laminin, gamma 2 |
| 3852 | KRT5 | Keratin 5 (epidermolysis bullosa simplex, Dowling-Meara/Kobner/Weber-Cockayne types) |
| 3675 | ITGA3 | Integrin, alpha 3 (antigen CD49C, alpha 3 subunit of VLA-3 receptor) |
| 6382 | SDC1 | Syndecan 1 |
| 3898 | LAD1 | Ladinin 1 |
| 11202 | KLK8 | Kallikrein 8 (neuropsin/ovasin) |
| 3854 | KRT6B | Keratin 6B |
| 57648 | KIAA1522 | KIAA1522 protein |
| 84951 | CTEN | C-terminal tensin-like |
| 9053 | MAP7 | Microtubule-associated protein 7 |
| 780 | DDR1 | Discoidin domain receptor family, member 1 |
| 247 | ALOX15B | Arachidonate 15-lipoxygenase, second type |
| 3728 | JUP | Junction plakoglobin |
| 5652 | PRSS8 | Protease, serine, 8 (prostasin) |
| 1830 | DSG3 | Desmoglein 3 (pemphigus vulgaris antigen) |
| 4128 | MAOA | Monoamine oxidase A |
| 1001 | CDH3 | Cadherin 3, type 1, P-cadherin (placental) |
| 79628 | KIAA1985 | KIAA1985 protein |
| 55287 | FLJ11036 | Hypothetical protein FLJ11036 |
| 3872 | KRT17 | Keratin 17 |
| 79413 | ZBED2 | Zinc finger, BED domain containing 2 |
| 23682 | RAB38 | RAB38, member RAS oncogene family |
| 5655 | KLK10 | Kallikrein 10 |
| 92359 | CRB3 | Crumbs homolog 3 (Drosophila) |
| 3664 | IRF6 | Interferon regulatory factor 6 |
| 118932 | ANKRD22 | Ankyrin repeat domain 22 |
| 244 | ANXA8 | Annexin A8 |
| 3691 | ITGB4 | Integrin, beta 4 |
| 10610 | SIAT7B | Sialyltransferase 7 ((alpha-N-acetylneuraminyl-2,3-beta-galactosyl-1,3)-N-acetyl galactosaminide alpha-2,6-sialyltransferase) B |
| 7378 | UPP1 | Uridine phosphorylase 1 |
| 57822 | TFCP2L4 | Transcription factor CP2-like 4 |
| 1515 | CTSL2 | Cathepsin L2 |
| 80157 | FLJ21511 | Hypothetical protein FLJ21511 |
| 150696 | PROM2 | Prominin 2 |
| 167153 | PAPD4 | PAP associated domain containing 4 |
| 6279 | S100A8 | S100 calcium binding protein A8 (calgranulin A) |
| 2709 | GJB5 | Gap junction protein, beta 5 (connexin 31.1) |
| 3552 | IL1A | Interleukin 1, alpha |
| 934 | CD24 | CD24 antigen (small cell lung carcinoma cluster 4 antigen) |
| 51599 | LISCH7 | Liver-specific bHLH-Zip transcription factor |
| 9982 | FGFBP1 | Fibroblast growth factor binding protein 1 |
| 64073 | C19orf33 | Chromosome 19 open reading frame 33 |
| 9048 | ARTN | Artemin |
| 2069 | EREG | Epiregulin |
| 126695 | FLJ34633 | Hypothetical protein FLJ34633 |
| 80004 | FLJ21918 | Hypothetical protein FLJ21918 |
| 3983 | ABLIM1 | Actin binding LIM protein 1 |
| 285489 | FLJ33718 | Hypothetical protein FLJ33718 |
| 2950 | GSTP1 | Glutathione S-transferase pi |
| 9938 | ARHGAP25 | Rho GTPase activating protein 25 |
| 11095 | ADAMTS8 | A disintegrin-like and metalloprotease (reprolysin type) with thrombospondin type 1 motif, 8 |
| 54751 | FBLP-1 | Filamin-binding LIM protein-1 |
| 27242 | TNFRSF21 | Tumor necrosis factor receptor superfamily, member 21 |
| 112616 | CKLFSF7 | Chemokine-like factor super family 7 |
| 113828 | LOC113828 | Hypothetical protein BC011204 |
| 3883 | KRTHA3A | Keratin, hair, acidic, 3A |
| 5268 | SERPINB5 | Serine (or cysteine) proteinase inhibitor, clade B (ovalbumin), member 5 |
| 2921 | CXCL3 | Chemokine (C-X-C motif) ligand 3 |
| 999 | CDH1 | Cadherin 1, type 1, E-cadherin (epithelial) |
| 26154 | ABCA12 | ATP-binding cassette, sub-family A (ABC1), member 12 |
| 8710 | SERPINB7 | Serine (or cysteine) proteinase inhibitor, clade B (ovalbumin), member 7 |
| 7739 | ZNF185 | Zinc finger protein 185 (LIM domain) |
| 50848 | F11R | F11 receptor |
| 57111 | RAB25 | RAB25, member RAS oncogene family |
| 10103 | TSPAN-1 | Tetraspan 1 |
| 84283 | MGC13102 | Hypothetical protein MGC13102 |
| 266977 | GPR110 | G protein-coupled receptor 110 |
| 2706 | GJB2 | Gap junction protein, beta 2, 26kDa (connexin 26) |
| 7718 | ZNF165 | Zinc finger protein 165 |
| 9414 | TJP2 | Tight junction protein 2 (zona occludens 2) |
| 2920 | CXCL2 | Chemokine (C-X-C motif) ligand 2 |
| 152028 | MGC34923 | Hypothetical protein MGC34923 |
| 1829 | DSG2 | Desmoglein 2 |
| 84069 | DKFZP434H2010 | Hypothetical protein DKFZp434H2010 |
| 3985 | LIMK2 | LIM domain kinase 2 |
| 6289 | SAA2 | Serum amyloid A2 |
| 57211 | GPR126 | G protein-coupled receptor 126 |
| 3557 | IL1RN | Interleukin 1 receptor antagonist |
| 51765 | MST4 | Mst3 and SOK1-related kinase |
| 135932 | FLJ90586 | Hypothetical protein FLJ90586 |
| 114569 | MAL2 | Mal, T-cell differentiation protein 2 |
| 3783 | KCNN4 | Potassium intermediate/small conductance calcium-activated channel, subfamily N, member 4 |
| 5724 | PTAFR | Platelet-activating factor receptor |
| 5834 | PYGB | Phosphorylase, glycogen; brain |
| 260436 | C4orf7 | Chromosome 4 open reading frame 7 |
| 5646 | PRSS3 | Protease, serine, 3 (mesotrypsin) |
| 2919 | CXCL1 | Chemokine (C-X-C motif) ligand 1 (melanoma growth stimulating activity, alpha) |
| 6692 | SPINT1 | Serine protease inhibitor, Kunitz type 1 |
| 64359 | NXN | Nucleoredoxin |
| 53905 | DUOX1 | Dual oxidase 1 |
| 131566 | DCBLD2 | Discoidin, CUB and LCCL domain containing 2 |
| 928 | CD9 | CD9 antigen (p24) |
| 2886 | GRB7 | Growth factor receptor-bound protein 7 |
| 10045 | SH2D3A | SH2 domain containing 3A |
| 347735 | TDE2L | Tumor differentially expressed 2-like |
| 5271 | SERPINB8 | Serine (or cysteine) proteinase inhibitor, clade B (ovalbumin), member 8 |
| 8076 | MFAP5 | Microfibrillar associated protein 5 |
| 284085 | FLJ40504 | Hypothetical protein FLJ40504 |
| 202915 | MGC9712 | Hypothetical protein MGC9712 |
| 5318 | PKP2 | Plakophilin 2 |
| 3875 | KRT18 | Keratin 18 |
| 127544 | IBRDC3 | IBR domain containing 3 |
| 84985 | BJ-TSA-9 | Hypothetical protein MGC14128 |
| 1645 | AKR1C1 | Aldo-keto reductase family 1, member C2 (dihydrodiol dehydrogenase 2; bile acid binding protein; 3-alpha hydroxysteroid dehydrogenase, type III) |
| 58495 | ZNF339 | Zinc finger protein 339 |
| 10205 | EVA1 | Epithelial V-like antigen 1 |
| 135250 | RAET1E | Retinoic acid early transcript 1E |
| 5774 | PTPN3 | Protein tyrosine phosphatase, non-receptor type 3 |
| 2152 | F3 | Coagulation factor III (thromboplastin, tissue factor) |
| 154810 | AMOTL1 | Angiomotin like 1 |
| 3655 | ITGA6 | Integrin, alpha 6 |
| 6280 | S100A9 | S100 calcium binding protein A9 (calgranulin B) |
| 25946 | ZNF385 | Zinc finger protein 385 |
| 6574 | SLC20A1 | Solute carrier family 20 (phosphate transporter), member 1 |
| 1832 | DSP | Desmoplakin |
| 2769 | GNA15 | Guanine nucleotide binding protein (G protein), alpha 15 (Gq class) |
| 84953 | FLJ14966 | Hypothetical protein FLJ14966 |
| 126353 | C19orf21 | Chromosome 19 open reading frame 21 |
| 1969 | EPHA2 | EPH receptor A2 |
| 27237 | ARHGEF16 | Rho guanine exchange factor (GEF) 16 |
| 3866 | KRT15 | Keratin 15 |
| 79623 | GALNT14 | UDP-N-acetyl-alpha-D-galactosamine:polypeptide N-acetylgalactosaminyltransferase 14 (GalNAc-T14) |
| 64065 | PERP | PERP, TP53 apoptosis effector |
| 894 | CCND2 | Cyclin D2 |
| 9076 | CLDN1 | Claudin 1 |
| 101 | ADAM8 | A disintegrin and metalloproteinase domain 8 |
| 164781 | FLJ25955 | Hypothetical protein FLJ25955 |
| 84817 | TXNL5 | Thioredoxin-like 5 |
| 1040 | CDS1 | CDP-diacylglycerol synthase (phosphatidate cytidylyltransferase) 1 |
| 25941 | C18orf10 | Chromosome 18 open reading frame 10 |
| 8045 | C11orf13 | Chromosome 11 open reading frame 13 |
| 219855 | SLC37A2 | Solute carrier family 37 (glycerol-3-phosphate transporter), member 2 |
| 11059 | WWP1 | WW domain containing E3 ubiquitin protein ligase 1 |
| 5317 | PKP1 | Plakophilin 1 (ectodermal dysplasia/skin fragility syndrome) |
| 558 | AXL | AXL receptor tyrosine kinase |
| 5874 | RAB27B | RAB27B, member RAS oncogene family |
| 1847 | DUSP5 | Dual specificity phosphatase 5 |
| 55765 | FLJ10901 | Hypothetical protein FLJ10901 |
| 9635 | CLCA2 | Chloride channel, calcium activated, family member 2 |
| 6699 | SPRR1B | Small proline-rich protein 1B (cornifin) |
| 84879 | FLJ14490 | Hypothetical protein FLJ14490 |
| 283635 | C14orf24 | Chromosome 14 open reading frame 24 |
| 25818 | KLK5 | Kallikrein 5 |
| 27071 | DAPP1 | Dual adaptor of phosphotyrosine and 3-phosphoinositides |
| 54101 | RIPK4 | Receptor-interacting serine-threonine kinase 4 |
| 4072 | TACSTD1 | Tumor-associated calcium signal transducer 1 |
| 4645 | MYO5B | Myosin VB |
| 259173 | ALS2CL | ALS2 C-terminal like |
| 64284 | RAB17 | RAB17, member RAS oncogene family |
| 51458 | RHCG | Rhesus blood group, C glycoprotein |
| 5597 | MAPK6 | Mitogen-activated protein kinase 6 |
| 154 | ADRB2 | Adrenergic, beta-2-, receptor, surface |
| 10480 | GA17 | Dendritic cell protein |
| 10620 | ARID3B | AT rich interactive domain 3B (BRIGHT- like) |
| 25816 | TNFAIP8 | Tumor necrosis factor, alpha-induced protein 8 |
| 3038 | HAS3 | Hyaluronan synthase 3 |
| 4319 | MMP10 | Matrix metalloproteinase 10 (stromelysin 2) |
| 2832 | GPR8 | G protein-coupled receptor 8 |
| 54894 | FLJ20315 | Hypothetical protein FLJ20315 |
| 10123 | ARL7 | ADP-ribosylation factor-like 7 |
| 79767 | ELMO3 | Engulfment and cell motility 3 (ced-12 homolog, C. elegans) |
| 23348 | DOCK9 | Dedicator of cytokinesis 9 |
| 10342 | TFG | TRK-fused gene |
| 1606 | DGKA | Diacylglycerol kinase, alpha 80kDa |
| 57707 | KIAA1609 | KIAA1609 protein |
| 90102 | PHLDB2 | Pleckstrin homology-like domain, family B, member 2 |
| 3885 | KRTHA4 | Keratin, hair, acidic, 4 |
| 29775 | CARD10 | Caspase recruitment domain family, member 10 |
| 64768 | C9orf12 | Chromosome 9 open reading frame 12 |
| 201799 | FLJ32028 | Hypothetical protein FLJ32028 |
| 2034 | EPAS1 | Endothelial PAS domain protein 1 |
| 51635 | DHRS7 | Dehydrogenase/reductase (SDR family) member 7 |
| 6590 | SLPI | Secretory leukocyte protease inhibitor (antileukoproteinase) |
| 4831 | NME2 | Non-metastatic cells 2, protein (NM23B) expressed in |
| 6288 | SAA1 | Serum amyloid A1 |
| 23002 | DAAM1 | Dishevelled associated activator of morphogenesis 1 |
| 54941 | RNF125 | Ring finger protein 125 |
| 55057 | AIM1L | Absent in melanoma 1-like |
| 84002 | B3GNT5 | UDP-GlcNAc:betaGal beta-1,3-N-acetylglucosaminyltransferase 5 |
| 5603 | MAPK13 | Mitogen-activated protein kinase 13 |
| 8407 | TAGLN2 | Transgelin 2 |
| 284252 | KCTD1 | Potassium channel tetramerisation domain containing 1 |
| 5355 | PLP2 | Proteolipid protein 2 (colonic epithelium-enriched) |
| 132 | ADK | Adenosine kinase |
| 831 | CAST | Calpastatin |
| 397 | ARHGDIB | Rho GDP dissociation inhibitor (GDI) beta |
| 1288 | COL4A6 | Collagen, type IV, alpha 6 |
| 55806 | HR | Hairless homolog (mouse) |
| 79781 | IQCA | IQ motif containing with AAA domain |
| 2150 | F2RL1 | Coagulation factor II (thrombin) receptor-like 1 |
| 29984 | RHOD | Ras homolog gene family, member D |
| 4170 | MCL1 | Myeloid cell leukemia sequence 1 (BCL2-related) |
| 3713 | IVL | Involucrin |
| 8942 | KYNU | Kynureninase (L-kynurenine hydrolase) |
| 3914 | LAMB3 | Laminin, beta 3 |
| 440702 | 0 | LOC440702 |
| 56169 | MLZE | Melanoma-derived leucine zipper, extra-nuclear factor |
| 80271 | ITPKC | Inositol 1,4,5-trisphosphate 3-kinase C |
| 4642 | MYO1D | Myosin ID |
| 5792 | PTPRF | Protein tyrosine phosphatase, receptor type, F |
| 3310 | HSPA6 | Heat shock 70kDa protein 6 (HSP70B') |
| 5898 | RALA | V-ral simian leukemia viral oncogene homolog A (ras related) |
| 55214 | LEPREL1 | Leprecan-like 1 |
| 93099 | ZD52F10 | Dermokine |
| 56940 | DUSP22 | Dual specificity phosphatase 22 |
| 26030 | PLEKHG3 | Pleckstrin homology domain containing, family G (with RhoGef domain) member 3 |
| 2137 | EXTL3 | Exostoses (multiple)-like 3 |
| 27076 | C4.4A | GPI-anchored metastasis-associated protein homolog |
| 892 | CCNC | Cyclin C |
| 286077 | FLJ46072 | FLJ46072 protein |
| 3673 | ITGA2 | Integrin, alpha 2 (CD49B, alpha 2 subunit of VLA-2 receptor) |
| 83481 | EPPK1 | Similar to epiplakin |
| 646 | BNC1 | Basonuclin 1 |
| 9289 | GPR56 | G protein-coupled receptor 56 |
| 130940 | LOC130940 | Hypothetical protein BC015395 |
| 30850 | HUMPPA | Paraneoplastic antigen |
| 51143 | DNCLI1 | Dynein, cytoplasmic, light intermediate polypeptide 1 |
| 254427 | C10orf47 | Chromosome 10 open reading frame 47 |
| 8578 | SCARF1 | Scavenger receptor class F, member 1 |
| 10098 | TM4SF9 | Transmembrane 4 superfamily member 9 |
| 3267 | HRB | HIV-1 Rev binding protein |
| 56262 | LRRC8 | Leucine rich repeat containing 8 |
| 1573 | CYP2J2 | Cytochrome P450, family 2, subfamily J, polypeptide 2 |
| 8797 | TNFRSF10A | Tumor necrosis factor receptor superfamily, member 10a |
| 84280 | GMRP-1 | K+ channel tetramerization protein |
| 441024 | MGC72244 | Similar to Bifunctional methylenetetrahydrofolate dehydrogenase/cyclohydrolase, mitochondrial precursor |
| 163732 | CITED4 | Cbp/p300-interacting transactivator, with Glu/Asp-rich carboxy-terminal domain, 4 |
| 6274 | S100A3 | S100 calcium binding protein A3 |
| 404203 | SPINK6 | Serine protease inhibitor, Kazal type 6 |
| 55857 | C20orf19 | Chromosome 20 open reading frame 19 |
| 5621 | PRNP | Prion protein (p27-30) (Creutzfeld-Jakob disease, Gerstmann-Strausler-Scheinker syndrome, fatal familial insomnia) |
| 26207 | PITPNC1 | Phosphatidylinositol transfer protein, cytoplasmic 1 |
| 129642 | OACT2 | O-acyltransferase (membrane bound) domain containing 2 |
| 22822 | PHLDA1 | Pleckstrin homology-like domain, family A, member 1 |
| 201562 | PTPLB | Protein tyrosine phosphatase-like (proline instead of catalytic arginine), member b |
| 6768 | ST14 | Suppression of tumorigenicity 14 (colon carcinoma, matriptase, epithin) |
| 5805 | PTS | 6-pyruvoyltetrahydropterin synthase |
| 483 | ATP1B3 | ATPase, Na+/K+ transporting, beta 3 polypeptide |
| 182 | JAG1 | Jagged 1 (Alagille syndrome) |
| 51719 | CAB39 | Calcium binding protein 39 |
| 7476 | WNT7A | Wingless-type MMTV integration site family, member 7A |
| 89796 | NAV1 | Neuron navigator 1 |
| 1849 | DUSP7 | Dual specificity phosphatase 7 |
| 338785 | KRT6L | Keratin 6L |
| 130340 | AP1S3 | Adaptor-related protein complex 1, sigma 3 subunit |
| 51186 | WBP5 | WW domain binding protein 5 |
| 55901 | THSD1 | Thrombospondin, type I, domain containing 1 |
| 55040 | EPN3 | Epsin 3 |
| 114757 | CYGB | Cytoglobin |
| 29094 | HSPC159 | HSPC159 protein |
| 3909 | LAMA3 | Laminin, alpha 3 |
| 10974 | C10orf116 | Chromosome 10 open reading frame 116 |
| 5791 | PTPRE | Protein tyrosine phosphatase, receptor type, E |
| 54429 | TAS2R5 | Taste receptor, type 2, member 5 |
| 23550 | PSD4 | Pleckstrin and Sec7 domain containing 4 |
| 23624 | CBLC | Cas-Br-M (murine) ecotropic retroviral transforming sequence c |
| 1012 | CDH13 | Cadherin 13, H-cadherin (heart) |
| 7326 | UBE2G1 | Ubiquitin-conjugating enzyme E2G 1 (UBC7 homolog, C. elegans) |
| 153769 | SH3RF2 | SH3 domain containing ring finger 2 |
| 10678 | B3GNT1 | UDP-GlcNAc:betaGal beta-1,3-N-acetylglucosaminyltransferase 1 |
| 9208 | LRRFIP1 | Leucine rich repeat (in FLII) interacting protein 1 |
| 54845 | FLJ20171 | Hypothetical protein FLJ20171 |
| 163259 | FLJ37099 | FLJ37099 protein |
| 10190 | TXNDC9 | Thioredoxin domain containing 9 |
| 5570 | PKIB | Protein kinase (cAMP-dependent, catalytic) inhibitor beta |
| 10458 | BAIAP2 | BAI1-associated protein 2 |
| 8190 | MIA | Melanoma inhibitory activity |
| 51062 | SPG3A | Spastic paraplegia 3A (autosomal dominant) |
| 1839 | DTR | Diphtheria toxin receptor (heparin-binding epidermal growth factor-like growth factor) |
| 8843 | GPR109B | G protein-coupled receptor 109B |
| 87 | ACTN1 | Actinin, alpha 1 |
| 5339 | PLEC1 | Plectin 1, intermediate filament binding protein 500kDa |
| 26499 | PLEK2 | Pleckstrin 2 |
| 4168 | MCF2 | MCF.2 cell line derived transforming sequence |
| 25800 | SLC39A6 | Solute carrier family 39 (zinc transporter), member 6 |
| 54453 | RIN2 | Ras and Rab interactor 2 |
| 1826 | DSCAM | Down syndrome cell adhesion molecule |
| 2729 | GCLC | Glutamate-cysteine ligase, catalytic subunit |
| 81839 | VANGL1 | Vang-like 1 (van gogh, Drosophila) |
| 8766 | RAB11A | RAB11A, member RAS oncogene family |
| 26232 | FBXO2 | F-box protein 2 |
| 54209 | TREM2 | Triggering receptor expressed on myeloid cells 2 |
| 8796 | SCEL | Sciellin |
| 6303 | SAT | Spermidine/spermine N1-acetyltransferase |
| 4242 | MFNG | Manic fringe homolog (Drosophila) |
| 2591 | GALNT3 | UDP-N-acetyl-alpha-D-galactosamine:polypeptide N-acetylgalactosaminyltransferase 3 (GalNAc-T3) |
| 3710 | ITPR3 | Inositol 1,4,5-triphosphate receptor, type 3 |
| 121512 | FGD4 | FYVE, RhoGEF and PH domain containing 4 |
| 121506 | FLJ32115 | Hypothetical protein FLJ32115 |
| 5939 | RBMS2 | RNA binding motif, single stranded interacting protein 2 |
| 26136 | TES | Testis derived transcript (3 LIM domains) |
| 113452 | BCLP | Beta-casein-like protein |
| 6307 | SC4MOL | Sterol-C4-methyl oxidase-like |
| 9019 | MPZL1 | Myelin protein zero-like 1 |
| 4493 | MT1E | Metallothionein 1E (functional) |
| 81704 | DOCK8 | Dedicator of cytokinesis 8 |
| 10818 | FRS2 | Fibroblast growth factor receptor substrate 2 |
| 966 | CD59 | CD59 antigen p18-20 (antigen identified by monoclonal antibodies 16.3A5, EJ16, EJ30, EL32 and G344) |
| 6835 | SURF2 | Surfeit 2 |
| 7850 | IL1R2 | Interleukin 1 receptor, type II |
| 10331 | B3GNT3 | UDP-GlcNAc:betaGal beta-1,3-N-acetylglucosaminyltransferase 3 |
| 3269 | HRH1 | Histamine receptor H1 |
| 222171 | LOC222171 | Hypothetical protein LOC222171 |
| 1948 | EFNB2 | Ephrin-B2 |
| 84679 | SLC9A7 | Solute carrier family 9 (sodium/hydrogen exchanger), isoform 7 |
| 7078 | TIMP3 | Tissue inhibitor of metalloproteinase 3 (Sorsby fundus dystrophy, pseudoinflammatory) |
| 84830 | C6orf105 | Chromosome 6 open reading frame 105 |
| 28966 | SNX24 | Sorting nexing 24 |
| 5639 | PRRG2 | Proline rich Gla (G-carboxyglutamic acid) 2 |
| 23541 | SEC14L2 | SEC14-like 2 (S. cerevisiae) |
| 7263 | TST | Thiosulfate sulfurtransferase (rhodanese) |
| 4496 | MT1H | Metallothionein 1H |
| 51222 | ZNF219 | Zinc finger protein 219 |
| 132014 | IL17RE | Interleukin 17 receptor E |
| 399687 | MYO18A | TGFB1-induced anti-apoptotic factor 1 |
| 8986 | RPS6KA4 | Ribosomal protein S6 kinase, 90kDa, polypeptide 4 |
| 29115 | HCNGP | Transcriptional regulator protein |
| 1021 | CDK6 | Cyclin-dependent kinase 6 |
| 6733 | SRPK2 | SFRS protein kinase 2 |
| 56606 | SLC2A9 | Solute carrier family 2 (facilitated glucose transporter), member 9 |
| 11221 | DUSP10 | Dual specificity phosphatase 10 |
| 56271 | BEXL1 | Brain expressed X-linked-like 1 |
| 10743 | RAI1 | Smith-Magenis syndrome chromosome region, candidate 6 |
| 55359 | STYK1 | Protein kinase STYK1 |
| 25855 | BRMS1 | Breast cancer metastasis suppressor 1 |
| 81579 | PLA2G12A | Phospholipase A2, group XIIA |
| 1594 | CYP27B1 | Cytochrome P450, family 27, subfamily B, polypeptide 1 |
| 4500 | MT1L | Metallothionein 1L |
| 7051 | TGM1 | Transglutaminase 1 (K polypeptide epidermal type I, protein-glutamine-gamma-glutamyltransferase) |
| 7170 | TPM3 | Tropomyosin 3 |
| 64129 | LCN7 | Lipocalin 7 |
| 1906 | EDN1 | Endothelin 1 |
| 1512 | CTSH | Cathepsin H |
| 9454 | HOMER3 | Homer homolog 3 (Drosophila) |
| 7105 | TM4SF6 | Transmembrane 4 superfamily member 6 |
| 1366 | CLDN7 | Claudin 7 |
| 54952 | SECP43 | TRNA selenocysteine associated protein |
| 5836 | PYGL | Phosphorylase, glycogen; liver (Hers disease, glycogen storage disease type VI) |
| 634 | CEACAM1 | Carcinoembryonic antigen-related cell adhesion molecule 1 (biliary glycoprotein) |
| 79864 | FLJ23554 | Hypothetical protein FLJ23554 |
| 6954 | TCP11 | T-complex 11 (mouse) |
| 54733 | SLC35F2 | Solute carrier family 35, member F2 |
| 166929 | MGC26963 | Hypothetical protein MGC26963 |
| 4188 | MDFI | MyoD family inhibitor |
| 10971 | YWHAQ | Tyrosine 3-monooxygenase/tryptophan 5-monooxygenase activation protein, theta polypeptide |
| 10892 | MALT1 | Mucosa associated lymphoid tissue lymphoma translocation gene 1 |
| 8664 | EIF3S7 | Eukaryotic translation initiation factor 3, subunit 7 zeta, 66/67kDa |
| 50649 | ARHGEF4 | Rho guanine nucleotide exchange factor (GEF) 4 |
| 2877 | GPX2 | Glutathione peroxidase 2 (gastrointestinal) |
| 4070 | TACSTD2 | Tumor-associated calcium signal transducer 2 |
| 126969 | MGC45474 | Hypothetical protein MGC45474 |
| 79631 | EFTUD1 | Elongation factor Tu GTP binding domain containing 1 |
| 55861 | C20orf35 | Chromosome 20 open reading frame 35 |
| 6233 | RPS27A | Ribosomal protein S27a |
| 374907 | B3GALT7 | UDP-Gal:betaGal beta 1,3-galactosyltransferase polypeptide 7 |
| 389337 | FLJ41603 | FLJ41603 protein |
| 8895 | CPNE3 | Copine III |
| 22824 | HSPA4L | Heat shock 70kDa protein 4-like |
| 3291 | HSD11B2 | Hydroxysteroid (11-beta) dehydrogenase 2 |
| 11313 | LYPLA2 | Lysophospholipase II |
| 93517 | HSPC105 | NAD(P) dependent steroid dehydrogenase-like |
| 128218 | MGC17299 | Hypothetical protein MGC17299 |
| 56649 | TMPRSS4 | Transmembrane protease, serine 4 |
| 9788 | MTSS1 | Metastasis suppressor 1 |
| 57085 | AGTRAP | Angiotensin II receptor-associated protein |
| 10053 | AP1M2 | Adaptor-related protein complex 1, mu 2 subunit |
| 50805 | IRX4 | Iroquois homeobox protein 4 |
| 79584 | FLJ12684 | Hypothetical protein FLJ12684 |
| 1942 | EFNA1 | Ephrin-A1 |
| 8848 | TGFB1I4 | Transforming growth factor beta 1 induced transcript 4 |
| 5066 | PAM | Peptidylglycine alpha-amidating monooxygenase |
| 2621 | GAS6 | Growth arrest-specific 6 |
| 84522 | GL009 | Hypothetical protein GL009 |
| 79817 | MOBKL2B | MOB1, Mps One Binder kinase activator-like 2B (yeast) |
| 57549 | IGSF9 | Immunoglobulin superfamily, member 9 |
| 147968 | CAPN12 | Calpain 12 |
| 9848 | MFAP3L | Microfibrillar-associated protein 3-like |
| 4053 | LTBP2 | Latent transforming growth factor beta binding protein 2 |
| 262 | AMD1 | Adenosylmethionine decarboxylase 1 |
| 57132 | CHMP1.5 | CHMP1.5 protein |
| 5055 | SERPINB2 | Serine (or cysteine) proteinase inhibitor, clade B (ovalbumin), member 2 |
| 954 | ENTPD2 | Ectonucleoside triphosphate diphosphohydrolase 2 |
| 143384 | C10orf46 | Chromosome 10 open reading frame 46 |
| 56905 | DKFZP434H132 | DKFZP434H132 protein |
| 4501 | MT1X | Metallothionein 1X |
| 79861 | TUBAL3 | Tubulin, alpha-like 3 |
| 6698 | SPRR1A | Small proline-rich protein 1A |
| 55605 | KIF21A | Kinesin family member 21A |
| 394 | ARHGAP5 | Rho GTPase activating protein 5 |
| 388115 | FLJ43339 | FLJ43339 protein |
| 115817 | DHRS1 | Dehydrogenase/reductase (SDR family) member 1 |
| 23286 | KIBRA | KIBRA protein |
| 5373 | PMM2 | Phosphomannomutase 2 |
| 79693 | FLJ23476 | Ischemia/reperfusion inducible protein |
| 84187 | FLJ22679 | Hypothetical protein FLJ22679 |
| 27075 | TM4SF13 | Transmembrane 4 superfamily member 13 |
| 57099 | AVEN | Apoptosis, caspase activation inhibitor |
| 3887 | KRTHB1 | Keratin, hair, basic, 1 |
| 1014 | CDH16 | Cadherin 16, KSP-cadherin |
| 11322 | EVER1 | Epidermodysplasia verruciformis 1 |
| 23075 | SWAP70 | SWAP-70 protein |
| 11137 | PWP1 | Nuclear phosphoprotein similar to S. cerevisiae PWP1 |
| 85377 | MICAL-L1 | MICAL-like 1 |
| 5645 | PRSS2 | Protease, serine, 2 (trypsin 2) |
| 79669 | FLJ23186 | Hypothetical protein FLJ23186 |
| 55700 | FLJ10350 | Hypothetical protein FLJ10350 |
| 760 | CA2 | Carbonic anhydrase II |
| 23149 | FCHO1 | FCH domain only 1 |
| 1825 | DSC3 | Desmocollin 3 |
| 54 | ACP5 | Acid phosphatase 5, tartrate resistant |
| 83987 | CCDC8 | Coiled-coil domain containing 8 |
| 1846 | DUSP4 | Dual specificity phosphatase 4 |
| 5671 | PSG3 | Pregnancy specific beta-1-glycoprotein 3 |
| 4495 | MT1G | Metallothionein 1G |
| 153562 | MARVELD2 | MARVEL domain containing 2 |
| 64866 | CDCP1 | CUB domain-containing protein 1 |
| 8091 | HMGA2 | High mobility group AT-hook 2 |
| 81551 | STMN4 | Stathmin-like 4 |
| 55117 | SLC6A15 | Solute carrier family 6 (neurotransmitter transporter), member 15 |
| 8609 | KLF7 | Kruppel-like factor 7 (ubiquitous) |
| 1316 | KLF6 | Kruppel-like factor 6 |
| 3280 | HES1 | Hairy and enhancer of split 1, (Drosophila) |
| 8446 | DUSP11 | Dual specificity phosphatase 11 (RNA/RNP complex 1-interacting) |
| 6282 | S100A11 | S100 calcium binding protein A11 (calgizzarin) |
| 3692 | ITGB4BP | Integrin beta 4 binding protein |
| 55686 | FLJ10116 | Whn-dependent transcript 2 |
| 5017 | OVOL1 | Ovo-like 1(Drosophila) |
| 5743 | PTGS2 | Prostaglandin-endoperoxide synthase 2 (prostaglandin G/H synthase and cyclooxygenase) |
| 9424 | KCNK6 | Potassium channel, subfamily K, member 6 |
| 5315 | PKM2 | Pyruvate kinase, muscle |
| 445328 | FLJ43692 | ARHGEF5-like |
| 3163 | HMOX2 | Heme oxygenase (decycling) 2 |
| 57146 | LOC57146 | Promethin |
| 51474 | EPLIN | Epithelial protein lost in neoplasm beta |
| 2125 | EVPL | Envoplakin |
| 29095 | ORMDL2 | ORM1-like 2 (S. cerevisiae) |
| 7168 | TPM1 | Tropomyosin 1 (alpha) |
| 5130 | PCYT1A | Phosphate cytidylyltransferase 1, choline, alpha isoform |
| 10848 | PPP1R13L | Protein phosphatase 1, regulatory (inhibitor) subunit 13 like |
| 23406 | COTL1 | Coactosin-like 1 (Dictyostelium) |
| 6508 | SLC4A3 | Solute carrier family 4, anion exchanger, member 3 |
| 2531 | FVT1 | Follicular lymphoma variant translocation 1 |
| 5708 | PSMD2 | Proteasome (prosome, macropain) 26S subunit, non-ATPase, 2 |
| 55907 | CMAS | Cytidine monophosphate N-acetylneuraminic acid synthetase |
| 23592 | LEMD3 | LEM domain containing 3 |
| 6575 | SLC20A2 | Solute carrier family 20 (phosphate transporter), member 2 |
| 23767 | FLRT3 | Fibronectin leucine rich transmembrane protein 3 |
| 1956 | EGFR | Epidermal growth factor receptor (erythroblastic leukemia viral (v-erb-b) oncogene homolog, avian) |
| 7692 | ZNF133 | Zinc finger protein 133 (clone pHZ-13) |
| 150094 | SNF1LK | SNF1-like kinase |
| 203427 | LOC203427 | Similar to solute carrier family 25 , member 16 |
| 3624 | INHBA | Inhibin, beta A (activin A, activin AB alpha polypeptide) |
| 9141 | PDCD5 | Programmed cell death 5 |
| 1307 | COL16A1 | Collagen, type XVI, alpha 1 |
| 4494 | MT1F | Metallothionein 1F (functional) |
| 91862 | MARVELD3 | MARVEL domain containing 3 |
| 51072 | C2orf4 | Chromosome 2 open reading frame 4 |
| 688 | KLF5 | Kruppel-like factor 5 (intestinal) |
| 29763 | PACSIN3 | Protein kinase C and casein kinase substrate in neurons 3 |
| 639 | PRDM1 | PR domain containing 1, with ZNF domain |
| 285381 | ZCSL2 | Zinc finger, CSL domain containing 2 |
| 23474 | ETHE1 | Ethylmalonic encephalopathy 1 |
| 1947 | EFNB1 | Ephrin-B1 |
| 54187 | NANS | N-acetylneuraminic acid synthase (sialic acid synthase) |
| 79183 | C20orf121 | Chromosome 20 open reading frame 121 |
| 55132 | FLJ10378 | FLJ10378 protein |
| 7037 | TFRC | Transferrin receptor (p90, CD71) |
| 5877 | RABIF | RAB interacting factor |
| 9718 | ECE2 | Endothelin converting enzyme 2 |
| 153830 | FLJ31951 | Hypothetical protein FLJ31951 |
| 23779 | ARHGAP8 | Rho GTPase activating protein 8 |
| 22899 | ARHGEF15 | Rho guanine nucleotide exchange factor (GEF) 15 |
| 79710 | ZCWCC2 | Zinc finger, CW type with coiled-coil domain 2 |
| 285672 | FLJ36754 | Hypothetical protein FLJ36754 |
| 6926 | TBX3 | T-box 3 (ulnar mammary syndrome) |
| 140823 | C20orf52 | Chromosome 20 open reading frame 52 |
| 121551 | BTBD11 | BTB (POZ) domain containing 11 |
| 9842 | PLEKHM1 | Pleckstrin homology domain containing, family M (with RUN domain) member 1 |
| 5744 | PTHLH | Parathyroid hormone-like hormone |
| 10435 | CDC42EP2 | CDC42 effector protein (Rho GTPase binding) 2 |
| 824 | CAPN2 | Calpain 2, (m/II) large subunit |
| 54918 | CKLFSF6 | Chemokine-like factor super family 6 |
| 4502 | MT2A | Metallothionein 2A |
| 157638 | NSE2 | Breast cancer membrane protein 101 |
| 65989 | EGFL9 | EGF-like-domain, multiple 9 |
| 161835 | FSIP1 | Fibrous sheath interacting protein 1 |
| 23607 | CD2AP | CD2-associated protein |
| 80176 | SSB1 | SPRY domain-containing SOCS box protein SSB-1 |
| 2888 | GRB14 | Growth factor receptor-bound protein 14 |
| 54869 | EPS8L1 | EPS8-like 1 |
| 6525 | SMTN | Smoothelin |
| 79148 | MMP28 | Matrix metalloproteinase 28 |
| 91749 | KIAA1919 | KIAA1919 |
| 9209 | LRRFIP2 | Leucine rich repeat (in FLII) interacting protein 2 |
| 29978 | UBQLN2 | Ubiquilin 2 |
| 7464 | CORO2A | Coronin, actin binding protein, 2A |
| 124739 | USP43 | Ubiquitin specific protease 43 |
| 59345 | GNB4 | Guanine nucleotide binding protein (G protein), beta polypeptide 4 |
| 4860 | NP | Nucleoside phosphorylase |
| 3936 | LCP1 | Lymphocyte cytosolic protein 1 (L-plastin) |
| 117195 | MRGX3 | G protein-coupled receptor MRGX3 |
| 84617 | TUBB6 | Tubulin, beta 6 |
| 9263 | STK17A | Serine/threonine kinase 17a (apoptosis-inducing) |
| 84958 | SYTL1 | Synaptotagmin-like 1 |
| 90060 | JM11 | JM11 protein |
| 5364 | PLXNB1 | Plexin B1 |
| 9245 | GCNT3 | Glucosaminyl (N-acetyl) transferase 3, mucin type |
| 53836 | GPR87 | G protein-coupled receptor 87 |
| 5588 | PRKCQ | Protein kinase C, theta |
| 1718 | DHCR24 | 24-dehydrocholesterol reductase |
| 9536 | PTGES | Prostaglandin E synthase |
| 5328 | PLAU | Plasminogen activator, urokinase |
| 23085 | RAB6IP2 | RAB6 interacting protein 2 |
| 113444 | LOC113444 | Hypothetical protein BC011880 |
| 9322 | TRIP10 | Thyroid hormone receptor interactor 10 |
| 6478 | SIAH2 | Seven in absentia homolog 2 (Drosophila) |
| 400745 | LOC400745 | Hypothetical gene supported by AK124869 |
| 92002 | MGC29729 | Hypothetical protein MGC29729 |
| 2760 | GM2A | GM2 ganglioside activator |
| 5049 | PAFAH1B2 | Platelet-activating factor acetylhydrolase, isoform Ib, beta subunit 30kDa |
| 8644 | AKR1C3 | Aldo-keto reductase family 1, member C3 (3-alpha hydroxysteroid dehydrogenase, type II) |
| 6195 | RPS6KA1 | Ribosomal protein S6 kinase, 90kDa, polypeptide 1 |
| 144568 | FLJ25179 | Hypothetical protein FLJ25179 |
| 6785 | ELOVL4 | Elongation of very long chain fatty acids (FEN1/Elo2, SUR4/Elo3, yeast)-like 4 |
| 144406 | MGC33630 | Hypothetical protein MGC33630 |
| 7277 | TUBA1 | Tubulin, alpha 1 (testis specific) |
| 84659 | RNASE7 | Ribonuclease, RNase A family, 7 |
| 63971 | KIF13A | Kinesin family member 13A |
| 440958 | 0 | LOC440958 |
| 8877 | SPHK1 | Sphingosine kinase 1 |
| 7126 | TNFAIP1 | Tumor necrosis factor, alpha-induced protein 1 (endothelial) |
| 5473 | PPBP | Pro-platelet basic protein (chemokine (C-X-C motif) ligand 7) |
| 2039 | EPB49 | Erythrocyte membrane protein band 4.9 (dematin) |
| 6675 | UAP1 | UDP-N-acteylglucosamine pyrophosphorylase 1 |
| 1488 | CTBP2 | C-terminal binding protein 2 |
| 50863 | HNT | Neurotrimin |
| 5327 | PLAT | Plasminogen activator, tissue |
| 9525 | VPS4B | Vacuolar protein sorting 4B (yeast) |
| 10809 | STARD10 | START domain containing 10 |
| 5029 | P2RY2 | Purinergic receptor P2Y, G-protein coupled, 2 |
| 4253 | CTAGE5 | CTAGE family, member 5 |
| 135398 | C6orf141 | Chromosome 6 open reading frame 141 |
| 55890 | GPRC5C | G protein-coupled receptor, family C, group 5, member C |
| 50485 | SMARCAL1 | SWI/SNF related, matrix associated, actin dependent regulator of chromatin, subfamily a-like 1 |
| 2932 | GSK3B | Glycogen synthase kinase 3 beta |
| 1364 | CLDN4 | Claudin 4 |
| 3856 | KRT8 | Keratin 8 |
| 83548 | COG3 | Component of oligomeric golgi complex 3 |
| 5770 | PTPN1 | Protein tyrosine phosphatase, non-receptor type 1 |
| 6446 | SGK | Serum/glucocorticoid regulated kinase |
| 200150 | FLJ40773 | Hypothetical protein FLJ40773 |
| 55832 | TIP120A | TBP-interacting protein |
| 58504 | ARHGAP22 | Rho GTPase activating protein 22 |
| 8882 | ZNF259 | Zinc finger protein 259 |
| 57451 | ODZ2 | Odz, odd Oz/ten-m homolog 2 (Drosophila) |
| 84188 | MLSTD2 | Male sterility domain containing 2 |
| 92421 | Shax3 | Snf7 homologue associated with Alix 3 |
| 54508 | FLJ11235 | Hypothetical protein FLJ11235 |
| 23529 | CLC | Cardiotrophin-like cytokine |
| 7311 | UBA52 | Ubiquitin A-52 residue ribosomal protein fusion product 1 |
| 157695 | INM01 | Hypothetical protein INM01 |
| 4430 | MYO1B | Myosin IB |
| 23141 | KIAA0692 | KIAA0692 protein |
| 153090 | DAB2IP | DAB2 interacting protein |
| 195814 | RDHE2 | Epidermal retinal dehydrogenase 2 |
| 123036 | MTAC2D1 | Membrane targeting (tandem) C2 domain containing 1 |
| 253943 | YTHDF3 | YTH domain family, member 3 |
| 4638 | MYLK | Myosin, light polypeptide kinase |
| 80131 | FLJ23420 | Hypothetical protein FLJ23420 |
| 4162 | MCAM | Melanoma cell adhesion molecule |
| 29841 | TFCP2L2 | Transcription factor CP2-like 2 |
| 4627 | MYH9 | Myosin, heavy polypeptide 9, non-muscle |
| 7347 | UCHL3 | Ubiquitin carboxyl-terminal esterase L3 (ubiquitin thiolesterase) |
| 2113 | ETS1 | V-ets erythroblastosis virus E26 oncogene homolog 1 (avian) |
| 56971 | CEAL1 | Carcinoembryonic antigen-like 1 |
| 83608 | C18orf21 | Chromosome 18 open reading frame 21 |
| 84337 | MGC4549 | Hypothetical protein MGC4549 |
| 157697 | LOC157697 | Hypothetical protein LOC157697 |
| 5875 | RABGGTA | Rab geranylgeranyltransferase, alpha subunit |
| 5604 | MAP2K1 | Mitogen-activated protein kinase kinase 1 |
| 79666 | PLEKHF2 | Pleckstrin homology domain containing, family F (with FYVE domain) member 2 |
| 56672 | C11orf17 | Chromosome 11 open reading frame 17 |
| 7984 | ARHGEF5 | Rho guanine nucleotide exchange factor (GEF) 5 |
| 7328 | UBE2H | Ubiquitin-conjugating enzyme E2H (UBC8 homolog, yeast) |
| 27250 | PDCD4 | Programmed cell death 4 (neoplastic transformation inhibitor) |
| 84290 | CAPNS2 | Calpain, small subunit 2 |
| 2625 | GATA3 | GATA binding protein 3 |
| 7071 | KLF10 | Kruppel-like factor 10 |
| 84837 | C14orf128 | Chromosome 14 open reading frame 128 |
| 7020 | TFAP2A | Transcription factor AP-2 alpha (activating enhancer binding protein 2 alpha) |
| 1591 | CYP24A1 | Cytochrome P450, family 24, subfamily A, polypeptide 1 |
| 9516 | LITAF | Lipopolysaccharide-induced TNF factor |
| 2872 | MKNK2 | MAP kinase interacting serine/threonine kinase 2 |
| 2665 | GDI2 | GDP dissociation inhibitor 2 |
| 1212 | CLTB | Clathrin, light polypeptide (Lcb) |
| 345667 | LOC345667 | Similar to ADAMTS-10 precursor (A disintegrin and metalloproteinase with thrombospondin motifs 10) (ADAM-TS 10) (ADAM-TS10) |
| 3553 | IL1B | Interleukin 1, beta |
| 5891 | RAGE | Renal tumor antigen |
| 10116 | FEM1B | Fem-1 homolog b (C. elegans) |
| 64844 | AXOT | Axotrophin |
| 25825 | BACE2 | Beta-site APP-cleaving enzyme 2 |
| 9069 | CLDN12 | Claudin 12 |
| 9448 | MAP4K4 | Mitogen-activated protein kinase kinase kinase kinase 4 |
| 57458 | KIAA1145 | KIAA1145 protein |
| 4643 | MYO1E | Myosin IE |
| 55505 | NOLA3 | Nucleolar protein family A, member 3 (H/ACA small nucleolar RNPs) |
| 51382 | ATP6V1D | ATPase, H+ transporting, lysosomal 34kDa, V1 subunit D |
| 143 | PARP4 | Poly (ADP-ribose) polymerase family, member 4 |
| 10776 | ARPP-19 | Cyclic AMP phosphoprotein, 19 kD |
| 333926 | PPP2CZ | Protein phosphatase 2a, catalytic subunit, zeta isoform |
| 8718 | TNFRSF25 | Tumor necrosis factor receptor superfamily, member 25 |
| 51538 | PS1D | Putative S1 RNA binding domain protein |
| 10059 | DNM1L | Dynamin 1-like |
| 669 | BPGM | 2,3-bisphosphoglycerate mutase |
| 195 | AHNAK | AHNAK nucleoprotein (desmoyokin) |
| 55612 | C20orf42 | Chromosome 20 open reading frame 42 |
| 2004 | ELK3 | ELK3, ETS-domain protein (SRF accessory protein 2) |
| 4810 | NHS | Nance-Horan syndrome (congenital cataracts and dental anomalies) |
| 10427 | SEC24B | SEC24 related gene family, member B (S. cerevisiae) |
| 221393 | GPR115 | G protein-coupled receptor 115 |
| 51559 | TU12B1-TY | TU12B1-TY protein |
| 25824 | PRDX5 | Peroxiredoxin 5 |
| 171177 | RHOV | Ras homolog gene family, member V |
| 116154 | PHACTR3 | Phosphatase and actin regulator 3 |
| 10451 | VAV3 | Vav 3 oncogene |
| 7430 | VIL2 | Villin 2 (ezrin) |
| 51126 | NAT5 | N-acetyltransferase 5 (ARD1 homolog, S. cerevisiae) |
| 57050 | SAS10 | Disrupter of silencing 10 |
| 10550 | ARL6IP5 | ADP-ribosylation-like factor 6 interacting protein 5 |
| 9683 | N4BP1 | Nedd4 binding protein 1 |
| 956 | ENTPD3 | Ectonucleoside triphosphate diphosphohydrolase 3 |
| 10890 | RAB10 | RAB10, member RAS oncogene family |
| 1580 | CYP4B1 | Cytochrome P450, family 4, subfamily B, polypeptide 1 |
| 151009 | FLJ38359 | Hypothetical protein FLJ38359 |
| 197259 | FLJ34389 | Hypothetical protein FLJ34389 |
| 5306 | PITPNA | Phosphatidylinositol transfer protein, alpha |
| 162417 | NAGS | N-acetylglutamate synthase |
| 7867 | MAPKAPK3 | Mitogen-activated protein kinase-activated protein kinase 3 |
| 79729 | FLJ22938 | Hypothetical protein FLJ22938 |
| 7533 | YWHAH | Chromosome 22 open reading frame 24 |
| 50862 | RNF141 | Ring finger protein 141 |
| 6337 | SCNN1A | Sodium channel, nonvoltage-gated 1 alpha |
| 79977 | TFCP2L3 | Transcription factor CP2-like 3 |
| 27315 | FRAG1 | FGF receptor activating protein 1 |
| 57823 | SLAMF7 | SLAM family member 7 |
| 124411 | LOC124411 | Hypothetical protein LOC124411 |
| 55034 | MOCOS | Molybdenum cofactor sulfurase |
| 92086 | GGTLA4 | Gamma-glutamyltransferase-like activity 4 |
| 79768 | C15orf29 | Chromosome 15 open reading frame 29 |
| 4233 | MET | Met proto-oncogene (hepatocyte growth factor receptor) |
| 8634 | RTCD1 | RNA terminal phosphate cyclase domain 1 |
| 359948 | IRF2BP2 | Interferon regulatory factor 2 binding protein 2 |
| 55906 | KIAA1166 | KIAA1166 |
| 162394 | MGC19764 | Hypothetical protein MGC19764 |
| 55190 | NUDT11 | Nudix (nucleoside diphosphate linked moiety X)-type motif 11 |
| 578 | BAK1 | BCL2-antagonist/killer 1 |
| 8743 | TNFSF10 | Tumor necrosis factor (ligand) superfamily, member 10 |
| 54436 | SH3TC1 | SH3 domain and tetratricopeptide repeats 1 |
| 27300 | ZNF544 | Zinc finger protein 544 |
| 1647 | GADD45A | Growth arrest and DNA-damage-inducible, alpha |
| 64419 | FLJ22405 | Hypothetical protein FLJ22405 |
| 11067 | C10orf10 | Chromosome 10 open reading frame 10 |
| 9827 | KIAA0258 | KIAA0258 |
| 6002 | RGS12 | Regulator of G-protein signalling 12 |
| 56683 | C21orf59 | Chromosome 21 open reading frame 59 |
| 115572 | FAM46B | Family with sequence similarity 46, member B |
| 5917 | RARS | Arginyl-tRNA synthetase |
| 4891 | SLC11A2 | Solute carrier family 11 (proton-coupled divalent metal ion transporters), member 2 |
| 340719 | NANOS1 | Nanos homolog 1 (Drosophila) |
| 9404 | LPXN | Leupaxin |
| 201158 | LOC201158 | Similar to CGI-148 protein |
| 146512 | FLJ30679 | Hypothetical protein FLJ30679 |
| 140885 | PTPNS1 | Protein tyrosine phosphatase, non-receptor type substrate 1 |
| 27122 | DKK3 | Dickkopf homolog 3 (Xenopus laevis) |
| 54873 | PALMD | Palmdelphin |
| 11035 | RIPK3 | Receptor-interacting serine-threonine kinase 3 |
| 1159 | CKMT1 | Creatine kinase, mitochondrial 1 (ubiquitous) |
| 3939 | LDHA | Lactate dehydrogenase A |
| 3775 | KCNK1 | Potassium channel, subfamily K, member 1 |
| 3955 | LFNG | Lunatic fringe homolog (Drosophila) |
| 56938 | ARNTL2 | Aryl hydrocarbon receptor nuclear translocator-like 2 |
| 115825 | WDFY2 | WD repeat and FYVE domain containing 2 |
| 4909 | NTF5 | Neurotrophin 5 (neurotrophin 4/5) |
| 9645 | MICAL2 | Flavoprotein oxidoreductase MICAL2 |
| 440462 | 0 | Hypothetical gene supported by AK000477 |
| 2131 | EXT1 | Exostoses (multiple) 1 |
| 7976 | FZD3 | Frizzled homolog 3 (Drosophila) |
| 79912 | FLJ22028 | Hypothetical protein FLJ22028 |
| 59338 | PLEKHA1 | Pleckstrin homology domain containing, family A (phosphoinositide binding specific) member 1 |
| 7039 | TGFA | Transforming growth factor, alpha |
| 84333 | RNF159 | Ring finger protein (C3HC4 type) 159 |
| 9022 | CLIC3 | Chloride intracellular channel 3 |
| 51768 | TM7SF3 | Transmembrane 7 superfamily member 3 |
| 284114 | FLJ36878 | Hypothetical protein FLJ36878 |
| 10519 | CIB1 | Calcium and integrin binding 1 (calmyrin) |
| 54964 | FLJ20519 | Hypothetical protein FLJ20519 |
| 26872 | STEAP | Six transmembrane epithelial antigen of the prostate |
| 92912 | LOC92912 | Hypothetical protein LOC92912 |
| 5590 | PRKCZ | Protein kinase C, zeta |
| 84230 | FAD158 | Factor for adipocyte differentiation 158 |
| 4688 | NCF2 | Neutrophil cytosolic factor 2 (65kDa, chronic granulomatous disease, autosomal 2) |
| 25979 | DKFZp566O084 | DKFZP566O084 protein |
| 10188 | ACK1 | Activated Cdc42-associated kinase 1 |
| 64127 | CARD15 | Caspase recruitment domain family, member 15 |
| 60370 | AVPI1 | Arginine vasopressin-induced 1 |
| 6364 | CCL20 | Chemokine (C-C motif) ligand 20 |
| 3576 | IL8 | Interleukin 8 |
| 51361 | HOOK1 | Hook homolog 1 (Drosophila) |
| 55737 | VPS35 | Vacuolar protein sorting 35 (yeast) |
| 5464 | PP | Pyrophosphatase (inorganic) |
| 1475 | CSTA | Cystatin A (stefin A) |
| 90133 | LOC90133 | Hypothetical protein LOC90133 |
| 140576 | S100A16 | S100 calcium binding protein A16 |
| 2317 | FLNB | Filamin B, beta (actin binding protein 278) |
| 5966 | REL | V-rel reticuloendotheliosis viral oncogene homolog (avian) |
| 9997 | SCO2 | SCO cytochrome oxidase deficient homolog 2 (yeast) |
| 5092 | PCBD | 6-pyruvoyl-tetrahydropterin synthase/dimerization cofactor of hepatocyte nuclear factor 1 alpha (TCF1) |
| 51024 | TTC11 | Tetratricopeptide repeat domain 11 |
| 822 | CAPG | Capping protein (actin filament), gelsolin-like |
| 8482 | SEMA7A | Sema domain, immunoglobulin domain (Ig), and GPI membrane anchor, (semaphorin) 7A |
| 6397 | SEC14L1 | SEC14-like 1 (S. cerevisiae) |
| 54972 | HSPA5BP1 | Heat shock 70kDa protein 5 (glucose-regulated protein, 78kDa) binding protein 1 |
| 9413 | C9orf61 | Chromosome 9 open reading frame 61 |
| 6318 | SERPINB4 | Serine (or cysteine) proteinase inhibitor, clade B (ovalbumin), member 4 |
| 6584 | SLC22A5 | Solute carrier family 22 (organic cation transporter), member 5 |
| 51144 | HSD17B12 | Hydroxysteroid (17-beta) dehydrogenase 12 |
| 286343 | C9orf150 | Chromosome 9 open reading frame 150 |
| 79818 | ZNF552 | Zinc finger protein 552 |
| 55653 | BCAS4 | Breast carcinoma amplified sequence 4 |
| 859 | CAV3 | Caveolin 3 |
| 85439 | STN2 | Stonin 2 |
| 6242 | RTKN | Rhotekin |
| 343 | AQP8 | Aquaporin 8 |
| 4664 | NAB1 | NGFI-A binding protein 1 (EGR1 binding protein 1) |
| 6696 | SPP1 | Secreted phosphoprotein 1 (osteopontin, bone sialoprotein I, early T-lymphocyte activation 1) |
| 51442 | VGLL1 | Vestigial like 1 (Drosophila) |
| 10434 | LYPLA1 | Lysophospholipase I |
| 4301 | MLLT4 | Myeloid/lymphoid or mixed-lineage leukemia (trithorax homolog, Drosophila); translocated to, 4 |
| 10999 | SLC27A4 | Solute carrier family 27 (fatty acid transporter), member 4 |
| 1075 | CTSC | Cathepsin C |
| 84222 | DKFZp434N035 | Hypothetical protein DKFZp434N035 |
| 30001 | ERO1L | ERO1-like (S. cerevisiae) |
| 79642 | FLJ23548 | Hypothetical protein FLJ23548 |
| 10318 | TNIP1 | TNFAIP3 interacting protein 1 |
| 5097 | PCDH1 | Protocadherin 1 (cadherin-like 1) |
| 9620 | CELSR1 | Cadherin, EGF LAG seven-pass G-type receptor 1 (flamingo homolog, Drosophila) |
| 7097 | TLR2 | Toll-like receptor 2 |
| 78990 | OTUB2 | OTU domain, ubiquitin aldehyde binding 2 |
| 79679 | B7-H4 | Immune costimulatory protein B7-H4 |
| 10938 | EHD1 | EH-domain containing 1 |
| 27111 | SDCBP2 | Syndecan binding protein (syntenin) 2 |
| 8481 | OFD1 | Oral-facial-digital syndrome 1 |
| 8291 | DYSF | Dysferlin, limb girdle muscular dystrophy 2B (autosomal recessive) |
| 3459 | IFNGR1 | Interferon gamma receptor 1 |
| 23230 | VPS13A | Vacuolar protein sorting 13A (yeast) |
| 11012 | KLK11 | Kallikrein 11 |
| 3963 | LGALS7 | Lectin, galactoside-binding, soluble, 7 (galectin 7) |
| 55966 | SHREW1 | Transmembrane protein SHREW1 |
| 10557 | RPP38 | Ribonuclease P/MRP 38kDa subunit |
| 23022 | KIAA0992 | Palladin |
| 2041 | EPHA1 | EPH receptor A1 |
| 3976 | LIF | Leukemia inhibitory factor (cholinergic differentiation factor) |
| 9617 | MTRF1 | Mitochondrial translational release factor 1 |
| 5048 | PAFAH1B1 | Platelet-activating factor acetylhydrolase, isoform Ib, alpha subunit 45kDa |
| 5293 | PIK3CD | Phosphoinositide-3-kinase, catalytic, delta polypeptide |
| 5074 | PAWR | PRKC, apoptosis, WT1, regulator |
| 79956 | KIAA1815 | KIAA1815 |
| 51330 | TNFRSF12A | Tumor necrosis factor receptor superfamily, member 12A |
| 442780 | 0 | Hypothetical gene supported by BC043153; NM_178561 |
| 84641 | FLJ14753 | Hypothetical protein FLJ14753 |
| 51100 | SH3GLB1 | SH3-domain GRB2-like endophilin B1 |
| 80823 | BHLHB9 | Basic helix-loop-helix domain containing, class B, 9 |
| 6278 | S100A7 | S100 calcium binding protein A7 (psoriasin 1) |
| 56998 | CTNNBIP1 | Catenin, beta interacting protein 1 |
| 63970 | P53AIP1 | P53-regulated apoptosis-inducing protein 1 |
| 166824 | RASSF6 | Ras association (RalGDS/AF-6) domain family 6 |
| 79865 | TREML2 | Triggering receptor expressed on myeloid cells-like 2 |
| 23150 | FRMD4B | FERM domain containing 4B |
| 389119 | LOC389119 | Similar to RIKEN cDNA 6530418L21 |
| 4534 | MTM1 | Myotubularin 1 |
| 10282 | BET1 | BET1 homolog (S. cerevisiae) |
| 25923 | DKFZP564J0863 | DKFZP564J0863 protein |
| 6317 | SERPINB3 | Serine (or cysteine) proteinase inhibitor, clade B (ovalbumin), member 3 |
| 301 | ANXA1 | Annexin A1 |
| 6524 | SLC5A2 | Solute carrier family 5 (sodium/glucose cotransporter), member 2 |
| 55328 | C10orf59 | Chromosome 10 open reading frame 59 |
| 7849 | PAX8 | Paired box gene 8 |
| 282809 | TUWD12 | UDP-N-acetyl-alpha-D-galactosamine:polypeptide N-acetylgalactosaminyltransferase 4 (GalNAc-T4) |
| 55362 | C6orf110 | Chromosome 6 open reading frame 110 |
| 224 | ALDH3A2 | Aldehyde dehydrogenase 3 family, member A2 |
| 2983 | GUCY1B3 | Guanylate cyclase 1, soluble, beta 3 |
| 147040 | KCTD11 | Potassium channel tetramerisation domain containing 11 |
| 58530 | LY6G6D | Chromosome 6 open reading frame 21 |
| 11127 | KIF3A | Kinesin family member 3A |
| 388720 | 0 | Similar to bA92K2.2 (similar to ubiquitin) |
| 221302 | C6orf113 | Chromosome 6 open reading frame 113 |
| 219404 | MGC9850 | Polymerase (RNA) I polypeptide D, 16kDa |
| 10539 | TXNL2 | Thioredoxin-like 2 |
| 55760 | DHX32 | DEAH (Asp-Glu-Ala-His) box polypeptide 32 |
| 51104 | C9orf77 | Chromosome 9 open reading frame 77 |
| 11187 | PKP3 | Plakophilin 3 |
| 58516 | C12orf14 | Chromosome 12 open reading frame 14 |
| 9124 | PDLIM1 | PDZ and LIM domain 1 (elfin) |
| 84962 | JUB | Jub, ajuba homolog (Xenopus laevis) |
| 148304 | FLJ25078 | Hypothetical protein FLJ25078 |
| 1824 | DSC2 | Desmocollin 2 |
| 26494 | OR8G1P | Olfactory receptor, family 8, subfamily G, member 1 |
| 64928 | MRPL14 | Mitochondrial ribosomal protein L14 |
| 79719 | FLJ11506 | Hypothetical protein FLJ11506 |
| 64332 | NFKBIZ | Nuclear factor of kappa light polypeptide gene enhancer in B-cells inhibitor, zeta |
| 8601 | RGS20 | Regulator of G-protein signalling 20 |
| 122704 | MRPL52 | Mitochondrial ribosomal protein L52 |
| 9468 | PCYT1B | Phosphate cytidylyltransferase 1, choline, beta isoform |
| 4478 | MSN | Moesin |
| 137994 | LETM2 | Leucine zipper-EF-hand containing transmembrane protein 2 |
| 29062 | HSPC049 | HSPC049 protein |
| 88745 | C6orf153 | Chromosome 6 open reading frame 153 |
| 4504 | MT3 | Metallothionein 3 (growth inhibitory factor (neurotrophic)) |
| 2149 | F2R | Coagulation factor II (thrombin) receptor |
| 7278 | TUBA2 | Tubulin, alpha 2 |
| 2533 | FYB | FYN binding protein (FYB-120/130) |
| 9766 | KIAA0247 | KIAA0247 |
| 81577 | MGC11335 | Hypothetical protein MGC11335 |
| 8635 | RNASET2 | Ribonuclease T2 |
| 79739 | FLJ23033 | Hypothetical protein FLJ23033 |
| 7534 | YWHAZ | Tyrosine 3-monooxygenase/tryptophan 5-monooxygenase activation protein, zeta polypeptide |
| 23219 | FBXO28 | F-box protein 28 |
| 1436 | CSF1R | Colony stimulating factor 1 receptor, formerly McDonough feline sarcoma viral (v-fms) oncogene homolog |
| 7322 | UBE2D2 | Ubiquitin-conjugating enzyme E2D 2 (UBC4/5 homolog, yeast) |
| 53827 | FXYD5 | FXYD domain containing ion transport regulator 5 |
| 3958 | LGALS3 | Lectin, galactoside-binding, soluble, 3 (galectin 3) |
| 960 | CD44 | CD44 antigen (homing function and Indian blood group system) |
| 25906 | DKFZP564M082 | DKFZP564M082 protein |
| 998 | CDC42 | Cell division cycle 42 (GTP binding protein, 25kDa) |
| 4705 | NDUFA10 | NADH dehydrogenase (ubiquinone) 1 alpha subcomplex, 10, 42kDa |
| 9510 | ADAMTS1 | A disintegrin-like and metalloprotease (reprolysin type) with thrombospondin type 1 motif, 1 |
| 80149 | FLJ23231 | Hypothetical protein FLJ23231 |
| 10049 | DNAJB6 | DnaJ (Hsp40) homolog, subfamily B, member 6 |
| 6714 | SRC | V-src sarcoma (Schmidt-Ruppin A-2) viral oncogene homolog (avian) |
| 222484 | LNX2 | Ligand of numb-protein X 2 |
| 149647 | FLJ32796 | Hypothetical protein FLJ32796 |
| 10552 | ARPC1A | Actin related protein 2/3 complex, subunit 1A, 41kDa |
| 6385 | SDC4 | Syndecan 4 (amphiglycan, ryudocan) |
| 25874 | DKFZP564B167 | DKFZP564B167 protein |
| 27342 | RABGEF1 | RAB guanine nucleotide exchange factor (GEF) 1 |
| 10279 | PRSS16 | Protease, serine, 16 (thymus) |
| 126820 | NYD-SP29 | Testis development protein NYD-SP29 |
| 9227 | LRAT | Lecithin retinol acyltransferase (phosphatidylcholine--retinol O-acyltransferase) |
| 83861 | RSHL2 | Radial spokehead-like 2 |
| 3694 | ITGB6 | Integrin, beta 6 |
| 3949 | LDLR | Low density lipoprotein receptor (familial hypercholesterolemia) |
| 79083 | MLPH | Melanophilin |
| 10197 | PSME3 | Proteasome (prosome, macropain) activator subunit 3 (PA28 gamma; Ki) |
| 57103 | C12orf5 | Chromosome 12 open reading frame 5 |
| 9168 | TMSB10 | Thymosin, beta 10 |
| 114884 | OSBPL10 | Oxysterol binding protein-like 10 |
| 7348 | UPK1B | Uroplakin 1B |
| 151242 | PPP1R1C | Protein phosphatase 1, regulatory (inhibitor) subunit 1C |
| 192111 | MGC5352 | Hypothetical protein MGC5352 |
| 79139 | DERL1 | Der1-like domain family, member 1 |
| 55276 | PGM2 | Phosphoglucomutase 2 |
| 113763 | C7orf29 | Chromosome 7 open reading frame 29 |
| 114907 | FBXO32 | F-box protein 32 |
| 23400 | HSA9947 | Putative ATPase |
| 6277 | S100A6 | S100 calcium binding protein A6 (calcyclin) |
| 10577 | NPC2 | Niemann-Pick disease, type C2 |
| 197131 | UBR1 | Ubiquitin protein ligase E3 component n-recognin 1 |
| 59082 | ICEBERG | ICEBERG caspase-1 inhibitor |
| 9047 | SH2D2A | SH2 domain protein 2A |
| 23760 | PITPNB | Phosphatidylinositol transfer protein, beta |
| 79647 | FLJ12666 | Hypothetical protein FLJ12666 |
| 60673 | FLJ11773 | Hypothetical protein FLJ11773 |
| 8751 | ADAM15 | A disintegrin and metalloproteinase domain 15 (metargidin) |
| 54763 | ROPN1 | Ropporin, rhophilin associated protein 1 |
| 55971 | LOC55971 | Insulin receptor tyrosine kinase substrate |
| 3363 | HTR7 | 5-hydroxytryptamine (serotonin) receptor 7 (adenylate cyclase-coupled) |
| 284348 | FLJ30469 | Hypothetical protein FLJ30469 |
| 4690 | NCK1 | NCK adaptor protein 1 |
| 9452 | ITM2A | Integral membrane protein 2A |
| 3364 | HUS1 | HUS1 checkpoint homolog (S. pombe) |
| 146174 | LOC146174 | Hypothetical protein LOC146174 |
| 10978 | HEAB | ATP/GTP-binding protein |
| 54503 | ZDHHC13 | Zinc finger, DHHC domain containing 13 |
| 85480 | TSLP | Thymic stromal lymphopoietin |
| 2523 | FUT1 | Fucosyltransferase 1 (galactoside 2-alpha-L-fucosyltransferase) |
| 10179 | RBM7 | RNA binding motif protein 7 |
| 90865 | C9orf26 | Chromosome 9 open reading frame 26 (NF-HEV) |
| 201176 | ARHGAP27 | Rho GTPase activating protein 27 |
| 653145 | ANXA8L1 | Annexin A8-like 1 |
| 23403 | FBXO46 | F-box protein 46 |
| 4133 | MAP2 | Microtubule-associated protein 2 |
| 5245 | PHB | Prohibitin |
| 54935 | DUSP23 | Dual specificity phosphatase 23 |
| 839 | CASP6 | Caspase 6, apoptosis-related cysteine protease |
| 55239 | FLJ10826 | Hypothetical protein FLJ10826 |
| 8673 | VAMP8 | Vesicle-associated membrane protein 8 (endobrevin) |
| 11056 | DDX52 | DEAD (Asp-Glu-Ala-Asp) box polypeptide 52 |
| 7360 | UGP2 | UDP-glucose pyrophosphorylase 2 |
| 79084 | MEP50 | MEP50 protein |
| 9045 | RPL14 | Ribosomal protein L14 |
| 221035 | C10orf74 | Chromosome 10 open reading frame 74 |
| 83857 | ARG99 | ARG99 protein |
| 54602 | NDFIP2 | Nedd4 family interacting protein 2 |
| 160760 | TA-PP2C | T-cell activation protein phosphatase 2C |
| 4236 | MFAP1 | Microfibrillar-associated protein 1 |
| 55266 | TMEM19 | Transmembrane protein 19 |
| 6788 | STK3 | Serine/threonine kinase 3 (STE20 homolog, yeast) |
| 23593 | HEBP2 | Heme binding protein 2 |
| 54933 | RHBDL2 | Rhomboid, veinlet-like 2 (Drosophila) |
| 257364 | MGC32065 | Hypothetical protein MGC32065 |
| 55833 | UBAP2 | Ubiquitin associated protein 2 |
| 54809 | SAMD9 | Sterile alpha motif domain containing 9 |
| 9531 | BAG3 | BCL2-associated athanogene 3 |
| 5292 | PIM1 | Pim-1 oncogene |
| 11261 | CHP | Calcium binding protein P22 |
| 3400 | ID4 | Inhibitor of DNA binding 4, dominant negative helix-loop-helix protein |
| 399694 | RaLP | Rai-like protein |
| 54998 | AKIP | Aurora-A kinase interacting protein |
| 4499 | MT1K | Metallothionein 1K |
| 11010 | GLIPR1 | GLI pathogenesis-related 1 (glioma) |
| 10959 | RNP24 | Coated vesicle membrane protein |
| 134265 | FLJ36748 | Hypothetical protein FLJ36748 |
| 146223 | CKLFSF4 | Chemokine-like factor super family 4 |
| 55727 | BTBD7 | BTB (POZ) domain containing 7 |
| 84918 | LRP11 | Low density lipoprotein receptor-related protein 11 |
| 4794 | NFKBIE | Nuclear factor of kappa light polypeptide gene enhancer in B-cells inhibitor, epsilon |
| 25914 | RTTN | Rotatin |
| 338321 | NALP9 | NACHT, leucine rich repeat and PYD containing 9 |
| 80237 | ELL3 | Elongation factor RNA polymerase II-like 3 |
| 92714 | ARRDC1 | Arrestin domain containing 1 |
| 285761 | DCBLD1 | Discoidin, CUB and LCCL domain containing 1 |
| 11332 | BACH | Brain acyl-CoA hydrolase |
| 4490 | MT1B | Metallothionein 1B (functional) |
| 220441 | RNF152 | Ring finger protein 152 |
| 10466 | COG5 | Component of oligomeric golgi complex 5 |
| 54947 | FLJ20481 | Hypothetical protein FLJ20481 |
| 5868 | RAB5A | RAB5A, member RAS oncogene family |
| 88455 | ANKRD13 | Ankyrin repeat domain 13 |
| 50999 | CGI-100 | CGI-100 protein |
| 118881 | COMTD1 | Catechol-O-methyltransferase domain containing 1 |
| 10200 | MPHOSPH6 | M-phase phosphoprotein 6 |
| 93492 | TPTE2 | Transmembrane phosphoinositide 3-phosphatase and tensin homolog 2 |
| 83543 | C9orf58 | Chromosome 9 open reading frame 58 |
| 54839 | FLJ20156 | Hypothetical protein FLJ20156 |
| 8323 | FZD6 | Frizzled homolog 6 (Drosophila) |
| 127845 | FLJ42654 | FLJ42654 protein |
| 219 | ALDH1B1 | Aldehyde dehydrogenase 1 family, member B1 |
| 9919 | KIAA0310 | KIAA0310 |
| 2525 | FUT3 | Fucosyltransferase 3 (galactoside 3(4)-L-fucosyltransferase, Lewis blood group included) |
| 7096 | TLR1 | Toll-like receptor 1 |
| 346389 | 7A5 | Putative binding protein 7a5 |
| 5899 | RALB | V-ral simian leukemia viral oncogene homolog B (ras related; GTP binding protein) |
| 5500 | PPP1CB | Protein phosphatase 1, catalytic subunit, beta isoform |
| 51657 | MK-STYX | Map kinase phosphatase-like protein MK-STYX |
| 8553 | BHLHB2 | Basic helix-loop-helix domain containing, class B, 2 |
| 56937 | TMEPAI | Transmembrane, prostate androgen induced RNA |
| 8417 | STX7 | Syntaxin 7 |
| 140735 | Dlc2 | Dynein light chain 2 |
| 22906 | OIP106 | OGT(O-Glc-NAc transferase)-interacting protein 106 KDa |
| 2806 | GOT2 | Glutamic-oxaloacetic transaminase 2, mitochondrial (aspartate aminotransferase 2) |
| 4851 | NOTCH1 | Notch homolog 1, translocation-associated (Drosophila) |
| 10755 | RGS19IP1 | Regulator of G-protein signalling 19 interacting protein 1 |
| 22943 | DKK1 | Dickkopf homolog 1 (Xenopus laevis) |
| 3770 | KCNJ14 | Potassium inwardly-rectifying channel, subfamily J, member 14 |
| 30846 | EHD2 | EH-domain containing 2 |
| 3371 | TNC | Tenascin C (hexabrachion) |
| 254531 | LOC254531 | PLSC domain containing protein |
| 11179 | ZNF277 | Zinc finger protein (C2H2 type) 277 |
| 25843 | PREI3 | Preimplantation protein 3 |
| 114294 | LACTB | Lactamase, beta |
| 23387 | KIAA0999 | KIAA0999 protein |
| 6804 | STX1A | Syntaxin 1A (brain) |
| 8826 | IQGAP1 | IQ motif containing GTPase activating protein 1 |
| 64411 | ARAP3 | ARF-GAP, RHO-GAP, ankyrin repeat and plekstrin homology domains-containing protein 3 |
| 59084 | ENPP5 | Ectonucleotide pyrophosphatase/phosphodiesterase 5 (putative function) |
| 10397 | NDRG1 | N-myc downstream regulated gene 1 |
| 6281 | S100A10 | S100 calcium binding protein A10 (annexin II ligand, calpactin I, light polypeptide (p11)) |
| 3570 | IL6R | Interleukin 6 receptor |
| 10135 | PBEF1 | Pre-B-cell colony enhancing factor 1 |
| 80352 | RNF39 | Ring finger protein 39 |
| 219738 | C10orf35 | Chromosome 10 open reading frame 35 |
| 7178 | TPT1 | Tumor protein, translationally-controlled 1 |
| 126308 | MOBKL2A | MOB1, Mps One Binder kinase activator-like 2A (yeast) |
| 5818 | PVRL1 | Poliovirus receptor-related 1 (herpesvirus entry mediator C; nectin) |
| 3577 | IL8RA | Interleukin 8 receptor, alpha |
| 9732 | DOCK4 | Dedicator of cytokinesis 4 |
| 9925 | ZBTB5 | Zinc finger and BTB domain containing 5 |
| 7323 | UBE2D3 | Ubiquitin-conjugating enzyme E2D 3 (UBC4/5 homolog, yeast) |
| 955 | ENTPD6 | Ectonucleoside triphosphate diphosphohydrolase 6 (putative function) |
| 5663 | PSEN1 | Presenilin 1 (Alzheimer disease 3) |
| 2773 | GNAI3 | Guanine nucleotide binding protein (G protein), alpha inhibiting activity polypeptide 3 |
| 3857 | KRT9 | Keratin 9 (epidermolytic palmoplantar keratoderma) |
| 81559 | TRIM11 | Tripartite motif-containing 11 |
| 7871 | SLMAP | Sarcolemma associated protein |
| 2787 | GNG5 | Guanine nucleotide binding protein (G protein), gamma 5 |
| 127534 | GJB4 | Gap junction protein, beta 4 (connexin 30.3) |
| 29101 | HSPC182 | HSPC182 protein |
| 3600 | IL15 | Interleukin 15 |
| 3981 | LIG4 | Ligase IV, DNA, ATP-dependent |
| 55669 | MFN1 | Mitofusin 1 |
| 302 | ANXA2 | Annexin A2 |
| 6742 | SSBP1 | Single-stranded DNA binding protein 1 |
| 29957 | SLC25A24 | Solute carrier family 25 (mitochondrial carrier; phosphate carrier), member 24 |
| 6947 | TCN1 | Transcobalamin I (vitamin B12 binding protein, R binder family) |
| 81607 | PVRL4 | Poliovirus receptor-related 4 |
| 3755 | KCNG1 | Potassium voltage-gated channel, subfamily G, member 1 |
| 8851 | CDK5R1 | Cyclin-dependent kinase 5, regulatory subunit 1 (p35) |
| 130399 | ACVR1C | Activin A receptor, type IC |
| 1432 | MAPK14 | Mitogen-activated protein kinase 14 |
| 9211 | LGI1 | Leucine-rich, glioma inactivated 1 |
| 140739 | NCE2 | NEDD8-conjugating enzyme |
| 9182 | PAMCI | Peptidylglycine alpha-amidating monooxygenase COOH-terminal interactor |
| 51406 | NOL7 | Nucleolar protein 7, 27kDa |
| 64283 | RGNEF | Rho-guanine nucleotide exchange factor |
| 121053 | MGC40397 | Hypothetical protein MGC40397 |
| 644 | BLVRA | Biliverdin reductase A |
| 6139 | RPL17 | Ribosomal protein L17 |
| 3315 | HSPB1 | Heat shock 27kDa protein 1 |
| 8754 | ADAM9 | A disintegrin and metalloproteinase domain 9 (meltrin gamma) |
| 10473 | HMGN4 | High mobility group nucleosomal binding domain 4 |
| 7325 | UBE2E2 | Ubiquitin-conjugating enzyme E2E 2 (UBC4/5 homolog, yeast) |
| 11342 | RNF13 | Ring finger protein 13 |
| 9991 | ROD1 | ROD1 regulator of differentiation 1 (S. pombe) |
| 51451 | LCMT1 | Leucine carboxyl methyltransferase 1 |
| 978 | CDA | Cytidine deaminase |
| 26128 | KIAA1279 | KIAA1279 |
| 23271 | KIAA1078 | KIAA1078 protein |
| 4318 | MMP9 | Matrix metalloproteinase 9 (gelatinase B, 92kDa gelatinase, 92kDa type IV collagenase) |
| 4486 | MST1R | Macrophage stimulating 1 receptor (c-met-related tyrosine kinase) |
| 147463 | ANKRD29 | Ankyrin repeat domain 29 |
| 7128 | TNFAIP3 | Tumor necrosis factor, alpha-induced protein 3 |
| 1936 | EEF1D | Eukaryotic translation elongation factor 1 delta (guanine nucleotide exchange protein) |
| 4179 | MCP | Membrane cofactor protein (CD46, trophoblast-lymphocyte cross-reactive antigen) |
| 6134 | RPL10 | Ribosomal protein L10 |
| 84313 | MGC10540 | Hypothetical protein MGC10540 |
| 10487 | CAP1 | CAP, adenylate cyclase-associated protein 1 (yeast) |
| 55283 | MCOLN3 | Mucolipin 3 |
| 3726 | JUNB | Jun B proto-oncogene |
| 718 | C3 | Complement component 3 |
| 50618 | ITSN2 | Intersectin 2 |
| 10158 | MAP17 | Membrane-associated protein 17 |
| 58472 | SQRDL | Sulfide quinone reductase-like (yeast) |
| 5205 | ATP8B1 | ATPase, Class I, type 8B, member 1 |
| 63027 | C6orf85 | Chromosome 6 open reading frame 85 |
| 3892 | KRTHB6 | Keratin, hair, basic, 6 (monilethrix) |
| 2224 | FDPS | Farnesyl diphosphate synthase (farnesyl pyrophosphate synthetase, dimethylallyltranstransferase, geranyltranstransferase) |
| 83640 | C6orf119 | Chromosome 6 open reading frame 119 |
| 153129 | FLJ90709 | Hypothetical protein FLJ90709 |
| 10644 | IMP-2 | IGF-II mRNA-binding protein 2 |
| 51629 | CGI-69 | CGI-69 protein |
| 11321 | XAB1 | XPA binding protein 1 |
| 84978 | MGC14161 | Hypothetical protein MGC14161 |
| 10096 | ACTR3 | ARP3 actin-related protein 3 homolog (yeast) |
| 9412 | SURB7 | SRB7 suppressor of RNA polymerase B homolog (yeast) |
| 7136 | TNNI2 | Troponin I, skeletal, fast |
| 1437 | CSF2 | Colony stimulating factor 2 (granulocyte-macrophage) |
| 23259 | DDHD2 | DDHD domain containing 2 |
| 10803 | CCR9 | Chemokine (C-C motif) receptor 9 |
| 2582 | GALE | UDP-galactose-4-epimerase |
| 10904 | BLCAP | Bladder cancer associated protein |
| 80097 | FLJ14346 | Hypothetical protein FLJ14346 |
| 10787 | NCKAP1 | NCK-associated protein 1 |
| 5362 | PLXNA2 | Plexin A2 |
| 144501 | LOC144501 | Hypothetical protein LOC144501 |
| 4507 | MTAP | Methylthioadenosine phosphorylase |
| 3052 | HCCS | Holocytochrome c synthase (cytochrome c heme-lyase) |
| 6520 | SLC3A2 | Solute carrier family 3 (activators of dibasic and neutral amino acid transport), member 2 |
| 4140 | MARK3 | MAP/microtubule affinity-regulating kinase 3 |
| 92400 | RBM18 | RNA binding motif protein 18 |
| 64834 | ELOVL1 | Elongation of very long chain fatty acids (FEN1/Elo2, SUR4/Elo3, yeast)-like 1 |
| 51249 | LOC51249 | Hypothetical protein LOC51249 |
| 93273 | LEMD1 | LEM domain containing 1 |
| 1318 | SLC31A2 | Solute carrier family 31 (copper transporters), member 2 |
| 56300 | IL1F9 | Interleukin 1 family, member 9 |
| 83607 | MGC4268 | Hypothetical protein MGC4268 |
| 57162 | PELI1 | Pellino homolog 1 (Drosophila) |
| 2313 | FLI1 | Friend leukemia virus integration 1 |
| 96610 | LOC96610 | Hypothetical protein similar to KIAA0187 gene product |
| 1739 | DLG1 | Discs, large homolog 1 (Drosophila) |
| 112464 | PRKCDBP | Protein kinase C, delta binding protein |
| 59347 | FKSG2 | Apoptosis inhibitor |
| 3423 | IDS | Iduronate 2-sulfatase (Hunter syndrome) |
| 7264 | TSTA3 | Tissue specific transplantation antigen P35B |
| 55731 | FLJ10700 | Hypothetical protein FLJ10700 |
| 2717 | GLA | Galactosidase, alpha |
| 9547 | CXCL14 | Chemokine (C-X-C motif) ligand 14 |
| 57619 | ShrmL | Shroom-related protein |
| 113235 | MGC9564 | Similar to RIKEN cDNA 1110002C08 gene |
| 92689 | LOC92689 | Hypothetical protein BC001096 |
| 51271 | UBAP1 | Ubiquitin associated protein 1 |
| 28952 | CXorf37 | Chromosome X open reading frame 37 |
| 51493 | HSPC117 | Hypothetical protein HSPC117 |
| 65124 | C2orf26 | Chromosome 2 open reading frame 26 |
| 56990 | CDC42SE2 | CDC42 small effector 2 |
| 1843 | DUSP1 | Dual specificity phosphatase 1 |
| 11021 | RAB35 | RAB35, member RAS oncogene family |
| 125476 | C18orf37 | Chromosome 18 open reading frame 37 |
| 6416 | MAP2K4 | Mitogen-activated protein kinase kinase 4 |
| 84684 | INSM2 | Insulinoma-associated 2 |
| 28514 | DLL1 | Delta-like 1 (Drosophila) |
| 55144 | LRRC5 | Leucine rich repeat containing 5 |
| 11006 | LILRB4 | Leukocyte immunoglobulin-like receptor, subfamily B (with TM and ITIM domains), member 4 |
| 23250 | ATP11A | ATPase, Class VI, type 11A |
| 149421 | FLJ32206 | Hypothetical protein FLJ32206 |
| 80223 | RAB11FIP1 | RAB11 family interacting protein 1 (class I) |
| 51714 | SELT | Selenoprotein T |
| 113878 | DTX2 | Deltex homolog 2 (Drosophila) |
| 80124 | VCIP135 | Valosin-containing protein (p97)/p47 complex-interacting protein p135 |
| 10637 | LEFTY1 | Left-right determination factor 1 |
| 6652 | SORD | Sorbitol dehydrogenase |
| 8372 | HYAL3 | Hyaluronoglucosaminidase 3 |
| 55969 | C20orf24 | Chromosome 20 open reading frame 24 |
| 147179 | WIRE | WIRE protein |
| 54407 | SLC38A2 | Solute carrier family 38, member 2 |
| 64221 | ROBO3 | Roundabout, axon guidance receptor, homolog 3 (Drosophila) |
| 5525 | PPP2R5A | Protein phosphatase 2, regulatory subunit B (B56), alpha isoform |
| 5277 | PIGA | Phosphatidylinositol glycan, class A (paroxysmal nocturnal hemoglobinuria) |
| 2961 | GTF2E2 | General transcription factor IIE, polypeptide 2, beta 34kDa |
| 1500 | CTNND1 | Catenin (cadherin-associated protein), delta 1 |
| 51167 | NCB5OR | NADPH cytochrome B5 oxidoreductase |
| 4000 | LMNA | Lamin A/C |
| 8870 | IER3 | Immediate early response 3 |
| 1265 | CNN2 | Calponin 2 |
| 280664 | WFDC10B | WAP four-disulfide core domain 10B |
| 8649 | MAP2K1IP1 | Mitogen-activated protein kinase kinase 1 interacting protein 1 |
| 29842 | TFCP2L1 | Transcription factor CP2-like 1 |
| 154091 | SLC2A12 | Solute carrier family 2 (facilitated glucose transporter), member 12 |
